# Metabolites for monitoring symptoms and predicting remission in patients with depression who received electroconvulsive therapy: a pilot study

**DOI:** 10.1038/s41598-023-40498-7

**Published:** 2023-08-14

**Authors:** Takahito Uchida, Yuki Sugiura, Eiji Sugiyama, Rae Maeda, Kenji F. Tanaka, Makoto Suematsu, Masaru Mimura, Hiroyuki Uchida

**Affiliations:** 1 Department of Neuropsychiatry, Keio University School of Medicine, 35 Shinanomachi, Shinjuku-ku, Tokyo, 160-8582 Japan; 2 Department of Psychiatry, Melbourne Neuropsychiatry Centre, The University of Melbourne, Melbourne, Australia; 3 Department of Biochemistry and Integrative Medical Biology, Keio University School of Medicine, Tokyo, Japan; 4 Multiomics Platform, Center for Cancer Immunotherapy and Immunobiology, Kyoto University Graduate School of Medicine, Kyoto, Japan; 5 Department of Analytical and Bioanalytical Chemistry, School of Pharmaceutical Sciences, University of Shizuoka, Shizuoka, Japan; 6 Division of Brain Sciences, Institute for Advanced Medical Research, Keio University School of Medicine, Tokyo, Japan

**Keywords:** Mass spectrometry, Metabolomics, Depression, Biomarkers

## Abstract

The lack of biomarkers to monitor and predict the efficacy of electroconvulsive therapy (ECT) has hindered its optimal use. To establish metabolomic markers for monitoring and predicting the treatment efficacy of ECT, we comprehensively evaluated metabolite levels in patients with major depressive disorder (MDD) by performing targeted and non-targeted metabolomic analyses using plasma samples before and after the first, third, and final ECT sessions, and 3–7 days after the final session. We compared the plasma metabolomes of age- and sex-matched healthy controls (HCs). Thirteen hospitalized patients with MDD and their corresponding HCs were included in this study. We observed that patients with MDD exhibited lower levels of amino acids, including gamma-aminobutyric acid (GABA), and metabolites involved in tryptophan metabolism and the kynurenine pathway, and higher levels of cortisol at baseline. Furthermore, we investigated the relationship between metabolite levels and depression severity across seven measurement timepoints along with one correlation analysis and found that amino acids, including GABA and tryptophan catabolites, were significantly correlated with the severity of depression. Despite the exploratory nature of this study due to the limited sample size necessitating further validation, our findings suggest that the blood metabolic profile has potential as a biomarker for ECT.

## Introduction

### Biomarkers that monitor the efficacy of and predict response to ECT are needed

Depression is a serious psychiatric illness for which numerous treatment options are typically prescribed. However, only 33% of patients with major depressive disorder (MDD) achieve remission with the initial course of antidepressant drugs, which decreases to 13% with the fourth course^1^. For patients with treatment-resistant depression (TRD), who do not respond to antidepressant treatment, several treatment guidelines recommend electroconvulsive therapy (ECT), given its superior effectiveness compared to that of antidepressant treatment^2^. However, approximately half of patients with TRD fail to achieve remission, even with ECT. Additionally, ECT poses the risks of postictal delirium and autobiographical memory loss, causing hesitation among patients and physicians to use this treatment option, resulting in unsuccessful treatment. Prolonged depressive episodes impair social functioning and reduce responsiveness to antidepressant treatments, including ECT^3^. Therefore, there is an urgent need for methods that can monitor efficacy and predict ECT responses. However, to date, the prediction of the response to ECT has been primarily investigated based on clinical or demographic characteristics, and no biological predictors have yet been identified.

### Systemic biochemical changes induced by ECT

Post-ECT biochemical changes are diverse. The serum kynurenic acid/quinolinic acid level ratio, which was significantly lower in patients with MDD than in controls at baseline, significantly increased after three ECT sessions^4^. Similarly, the serum kynurenic acid/l-tryptophan (Trp) level ratio was negatively associated with depressive symptoms in patients with depression who received ECT^5^. Hypothalamic–pituitary–adrenal (HPA) axis hyperactivation at baseline has been reported to predict subsequent ECT efficacy^6^. Additionally, other biochemical changes, including in neurotrophic factors, the immune system, and monoamine neurotransmitters and neurogenesis, have been reported after ECT^7^.

Thus, ECT causes systemic changes in multiple pathways, and a comprehensive assessment may best evaluate ECT-induced biochemical changes. “Omics” approaches have recently garnered widespread attention as a comprehensive method of measuring biological materials. Metabolomics, which involves the profiling of small-molecule metabolites, is advantageous because it measures both genomic and environmental variables. Metabolomic analysis of urine, blood, and cerebrospinal fluid in patients with depression has revealed abnormalities in mitochondrial/energy metabolism, neural integrity, and signaling/neurotransmission^8^. However, this approach has not yet been extensively utilized to evaluate the response to ECT. Here, we conducted a longitudinal, targeted, and non-targeted metabolomic study to identify metabolites that could predict remission following ECT in patients with depression through multiple metabolomic assessments immediately before and after the ECT sessions. In this study, we aimed to identify baseline characteristics of metabolite levels in patients with MDD and healthy controls (HCs). Additionally, we sought to identify specific blood metabolites that could be used to monitor the effects of ECT. Moreover, as part of our exploratory analysis, we aimed to identify potential blood metabolites that could predict remission after ECT by examining the changes in metabolite levels during the first ECT session.

## Results

### Demographic and clinical characteristics

This study included 13 hospitalized patients with MDD, and age- and sex-matched healthy individuals. All subjects were free of malignancies or inflammatory diseases that required treatment, and none were taking anti-inflammatory medications, including non-steroidal anti-inflammatory drugs. All patients diagnosed with MDD were taking antidepressants when they underwent ECT for depressive symptoms. ECT was generally effective, and the mean (± standard deviation) total Montgomery–Asberg Depression Rating Scale (MADRS) score decreased from 32.4 ± 11.7 at baseline (T1) to 9.8 ± 11.5 after the final ECT session (T6), and the total Quick Inventory of Depressive Symptomatology (QIDS) score decreased from 16.6 ± 6.4 at baseline (T1) to 5.7 ± 3.3 after the final ECT session (T6). Nine patients (mean age 55.6 ± 17.8 years, four males) achieved remission, while the other four patients (mean age 60.0 ± 12.0 years, three males) did not. Within the remission group, one patient achieved remission after the fourth ECT session, leading to the cessation of subsequent ECT sessions due to amnesic symptoms. Therefore, only the data collected from T1 to T4 were used for the analysis of this individual. Four patients were discharged before the final assessment (T7). As shown in Table 1, the remission group had significantly lower QIDS scores at baseline and lower MADRS total scores after the final ECT session. Other demographic and clinical characteristics did not differ significantly between the remission and non-remission groups.

**Table 1:** Demographic and Clinical Characteristics of Included Participants and Controls.

Characteristics	Controls (n = 13)	Remission (n = 9)	Non-remission (n = 4)	Remission versus non-remission *p* value
Age, years	53.7 ± 14.4	55.6 ± 17.8	60.0 ± 12.0	0.66
Male sex, n	7 (53.8)	4 (44.4)	3 (75.0)	0.56
Education, years	14.5 ± 1.8	14.2 ± 1.9	15.0 ± 2.0	0.51
Age at MDD onset, years	n/a	47.9 ± 20.6	45.0 ± 12.9	0.80
Family history of MDD, n	3 (23.1)	3 (33.3)	1 (25.0)	0.99
Current smoking, n	2 (15.4)	1 (11.1)	0 (0)	0.99
Current drinking, n	5 (38.5)	0 (0)	2 (50.0)	0.08
Previous episode, n	n/a	2.2 ± 2.6	1.8 ± 1.3	0.74
Previous admission, n	n/a	1.6 ± 2.6^a^	1.0 ± 1.2	0.70
Present episode, months	n/a	10.1 ± 7.9	13.8 ± 11.3	0.51
Psychotic features, n	n/a	1 (11.1)	2 (50.0)	0.20
Melancholic features, n	n/a	5 (55.6)	3 (75.0)	0.99
Catatonic features, n	n/a	0 (0)	1 (25.0)	0.31
Antidepressants, n	0 (0)	9 (100)	4 (100)	n/a
Antipsychotics, n	0 (0)	4 (44.4)	3 (75.0)	0.56
Hypnotics, n	0 (0)	5 (55.6)	4 (100)	0.23
MADRS total score				
Before ECT	n/a	31.2 ± 11.1	33.3 ± 13.6	0.78
After the third ECT	n/a	22.3 ± 16.7	32.0 ± 19.8	0.38
After the final ECT	n/a	4.6 ± 3.0^a^	20.0 ± 16.0	0.02
QIDS total score				
Before ECT	n/a	13.4 ± 5.4^a^	21.8 ± 3.3	0.02
After the third ECT	n/a	11.9 ± 7.8	11.0 ± 3.6^a^	0.86
After the final ECT	n/a	4.6 ± 2.9^a^	8.3 ± 2.6	0.06
ECT parameters				
First ECT charge (mC)	n/a	173.4 ± 80.1	145.2 ± 24.2	0.51
Last ECT charge (mC)	n/a	330.6 ± 147.7^a^	428.4 ± 120.0	0.27
ECT sessions, n	n/a	8.6 ± 2.7	10.3 ± 1.3	0.26

Values are shown as mean ± S.D. or n (%).

^a^One missing value.

ECT, Electroconvulsive Therapy; MADRS, Montgomery-Asberg Depression Rating Scale; MDD, Major Depressive Disorder; Quick Inventory of Depressive Symptomatology Self-Reported version; QIDS.

### Baseline characteristics of plasma metabolite levels of patients with MDD and HCs

**Figure 1:**
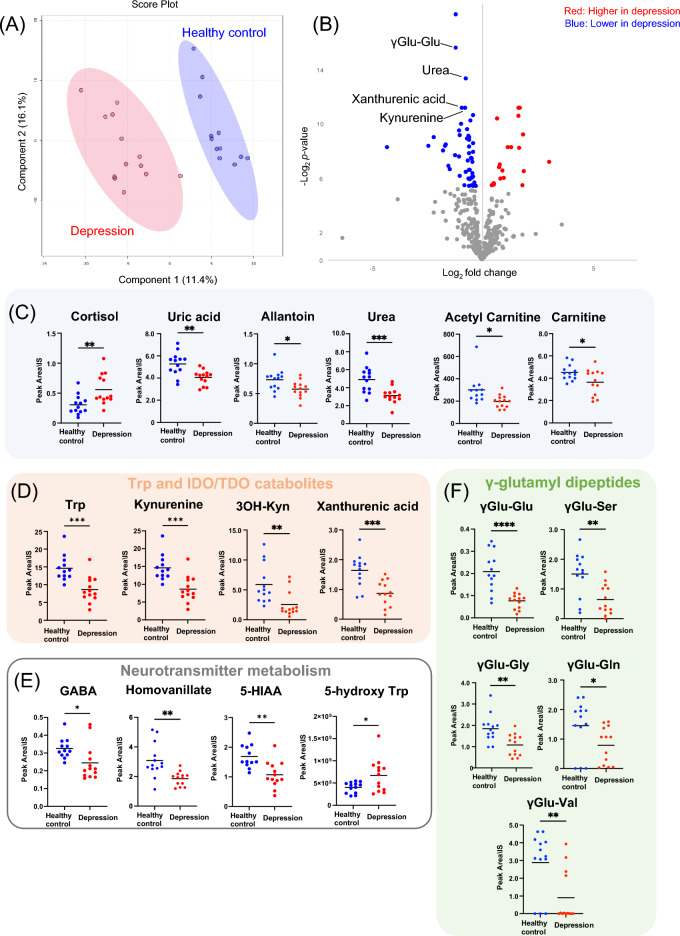
Plasma metabolite levels of MDD and HCs at baseline. PLS-DA score plot showing that patients with MDD and HCs formed different clusters (**A**). PLS-DA cross-validation details and variable importance in projection (VIP) scores for Fig. 1A were shown in Fig. S1 and Table S1, respectively. In addition, VIP scores for Fig. 1A were shown in Table S2. Volcano plot comparing the levels of metabolites in patients with MDD and HCs at the baseline. The blue and red dots represent significantly (*p* < 0.05) downregulated and upregulated metabolites in patients with MDD (**B**). Metabolites showed statistical differences in levels between patients with MDD and HCs at the baseline (**C**–**F**). Abbreviations: GABA, gamma-aminobutyric acid; HC, healthy control; IDO, indoleamine 2,3-dioxygenase; MDD, major depressive disorder; PLS-DA, partial least-squares discriminant analysis; TDO, tryptophan 2,3-dyoxygenase; Trp, tryptophan; γGlu-Glu, gamma glutamyl-glutamic acid; γGlu-Ser, gamma glutamylserine; γGlu-Gly, gamma glutamylglycine; γGlu-Gln, gamma glutamylglutamine; γGlu-Val, gamma glutamylvaline; 3OH Kyn, 3-hydrokynurenine; 5-HIAA, 5-hydroxyindoleacetic acid. *p < 0.05, **p < 0.01, ***p < 0.001.

Partial least-squares discriminant analysis (PLS-DA), which included the levels of 357 plasma metabolites obtained from targeted and non-targeted metabolome analysis as variables, revealed that patients with MDD and HCs formed distinct clusters (Fig. 1A, Fig. S1, Table S1, and Table S2). Furthermore, volcano plot analysis revealed that γ-glutamyl dipeptide (γGlu-Glu), identified in the non-targeted analysis, was the most significantly different metabolite in patients compared with that of HCs (Fig. 1B and Table S3). The hierarchical clustering analysis (HCA) of these metabolites showed higher levels of cortisol, choline, N-acetyl aspartate, ketone bodies, and inorganic phosphates, and lower levels of carnitine derivatives, purine metabolites, Trp and kynurenine (Kyn) pathway catabolites, γGlu-Glu, urea, carnitines, and gamma-aminobutyric acid (GABA) in patients (Fig. S2). A comparison of the levels of each metabolite identified by volcano plot analysis and HCA revealed significantly higher cortisol levels in patients with MDD. In contrast, the levels of purine metabolites, carnitine derivatives, metabolites involved in Trp metabolism and the Kyn pathway, GABA, metabolites involved in neurotransmitter metabolism, and γGlu-Glu were significantly lower in patients with MDD (Fig. 1C–F).

### Correlations between plasma metabolite levels and depressive symptoms during ECT

**Figure 2:**
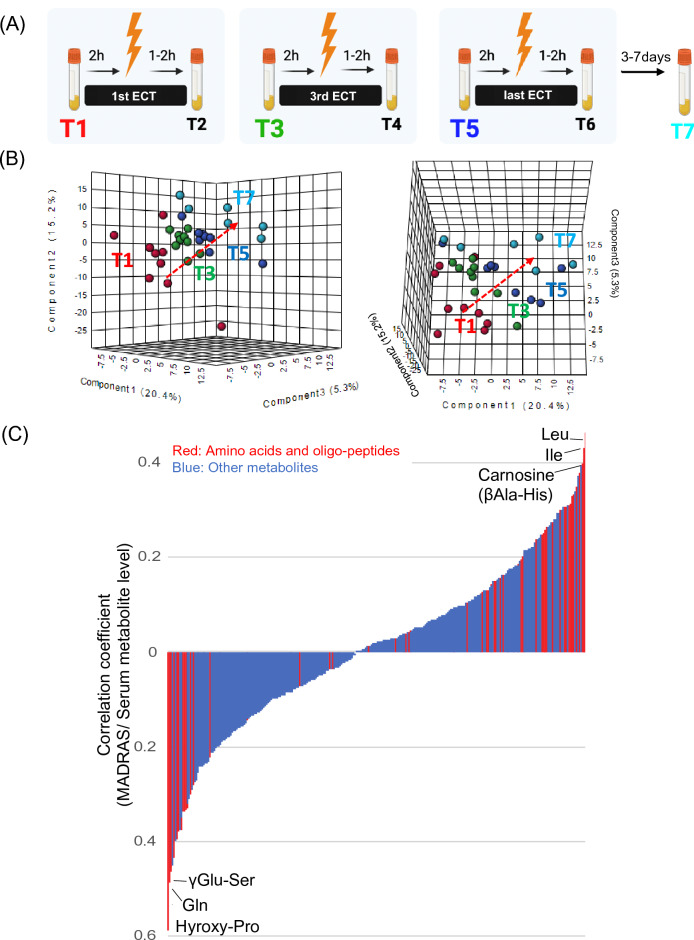
Correlations between plasma metabolite levels and depressive symptoms during ECT. Longitudinal change in plasma metabolite over three pre-ECT session time points (T1, T3, and T5) and for 3 days after the final ECT session (T7) were assessed (**A**). PLS-DA score plot showing the gradual change in levels of metabolites during the course of ECT in patients with MDD (**B**). VIP scores for Fig. 2B were shown in Table S4. The bar chart indicating Pearson’s correlation between the level of each metabolite and the MADRS score, with the amino acids, amino acid derivatives, and oligopeptides were highlighted in red (**C**). Abbreviations: ECT, electroconvulsive therapy; MDD, major depressive disorder; Gln, glutamine; Leu, leucine; MADRS, Montgomery–Asberg Depression Rating Scale; Ile, isoleucine; PLS-DA, partial least-squares discriminant analysis; βAla-His, beta alanylhistidine; γGlu-Ser, gamma glutamylserine.

PLS-DA of the 357 metabolites obtained from targeted and non-targeted metabolome analyses during ECT (T1, T3, T5, and T7) in patients with MDD (Fig. 2A) showed that the plasma metabolome gradually changed over the course of ECT (Fig. 2B and Table S4).

We then investigated the correlation between metabolite levels and depression severity across four measurement time points, along with a correlation analysis, to identify specific metabolites that could act as companion markers for ECT efficacy. (Fig. S3). The results showed that several amino acids and their derivatives, including GABA, oligopeptides, and kynurenine pathway metabolites, were significantly correlated with depression severity (Fig. 2C, Tables S5 and S6). The metabolites that showed baseline differences between patients with MDD and HCs, such as Trp catabolites, GABA, and γGlu-Glu, also showed significant correlations with depression severity during ECT (Fig. S4).

### Changes in plasma metabolites following the first ECT session as predictors of subsequent remission: an exploratory analysis

**Figure 3:**
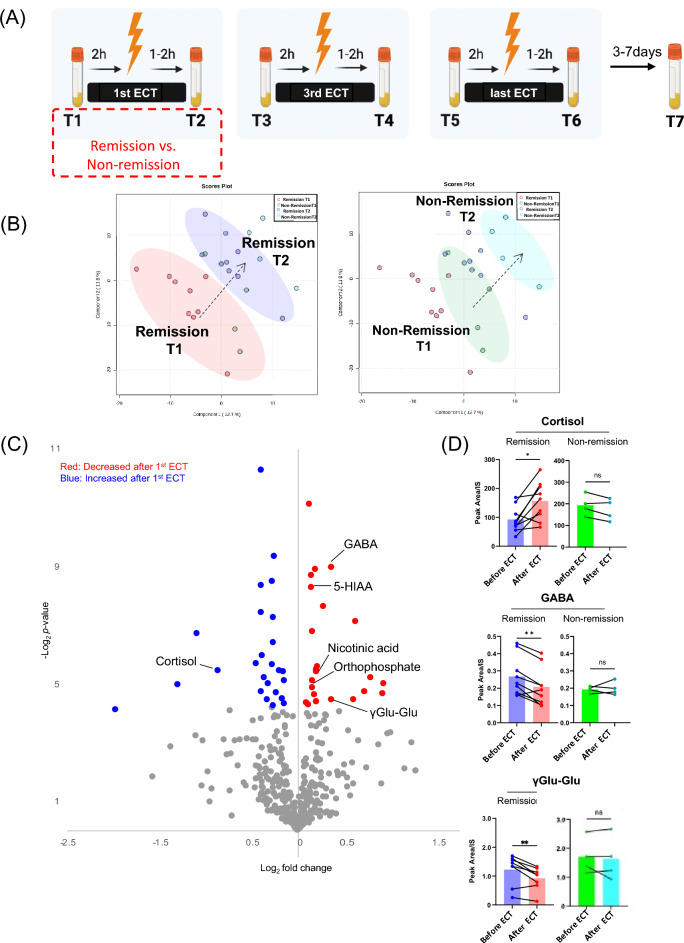
Changes in plasma metabolites levels by the first ECT session in remission and non-remission groups. Plasma metabolites fluctuation by the first ECT session were assessed in the remission and the non-remission groups (**A**). PLS-DA score plot showing the change of levels of metabolites between T1 and T2 (**B** left). PLS-DA score plot also showed that the remission and the non-remission groups formed different clusters (**B** right). VIP scores for Fig. 3B were shown in Table S7. Volcano plot comparing the levels of metabolites between T1 and T2 in the remission group. The blue and red dots represent significantly (*p* < 0.05) downregulated and upregulated metabolites (**C**). The changes in levels of some metabolites levels significantly changed by the first ECT in the remission group, whereas not in the non-remission group (**D**). Abbreviations: ECT, electroconvulsive therapy; GABA, gamma-aminobutyric acid; γGlu-Glu, gamma glutamyl-glutamic acid. *p < 0.05, **p < 0.01.

PLS-DA, which included the levels of 357 plasma metabolites obtained from targeted and non-targeted metabolome analyses before and after the first ECT session (T1 and T2) in patients with MDD (Fig. 3A and Table S7), showed a marked change in the levels of metabolites before and after the first ECT session in both the remission (n = 9) and non-remission groups (n = 4) (Fig. 3B, left). Furthermore, the cluster migration distance, which indicates the extent of metabolomic shift, appeared to be greater in the remission group than in the non-remission group (Fig. 3B, right). Volcano plot analysis identified metabolites that exhibited significant changes in the first ECT session in the remission group and revealed that the levels of metabolites, that is, amino acids, their catabolites, and derivatives, significantly decreased, while the levels of cortisol significantly increased (Fig. 3C, Table S8). Changes in the levels of some metabolites, such as cortisol, GABA, γ-glutamyl glutamate, **5**-hydroxyindoleacetic acid (5-HIAA), nicotinic acid, and orthophosphate, were not significant in the non-remission group (Fig. 3D and Fig. S5).

## Discussion

To the best of our knowledge, this is the first study to evaluate the cross-sectional differences in metabolite levels in patients with MDD who underwent ECT, as well as their longitudinal changes, using targeted and non-targeted metabolomics analysis. Comprehensive metabolomic analysis enabled the identification of metabolites that have not been previously reported as biomarkers of MDD. Metabolomics is a sensitive measurement technique that is affected by various factors, including diet and physical activity. In the present study, because ECT was performed on patients with MDD in an inpatient setting, diet, physical activity, and fasting were controlled under hospital supervision, which contributed to eliminating these confounding factors. In particular, the comparison of metabolite levels before and after ECT sessions allowed for an analysis in which various confounding factors were excluded, thereby capturing the biological changes that occur with ECT. First, this study assessed the cross-sectional differences in plasma metabolite profiles between patients and controls and found that metabolites such as cortisol, urea, uric acid, and kynurenine pathway metabolites differed between the patient and control groups. These results are consistent with those of previous studies^9–11^, and confirm the accuracy of the methodology used in this study. Moreover, we detected significant differences in metabolites that were not included in standard compound libraries used in conventional targeted metabolome analysis, such as amino acids and γGlu-Glu, because we applied non-targeted metabolomic analysis in the present study. Next, we evaluated the longitudinal changes in plasma metabolite levels during ECT and found that metabolites including several amino acids, GABA, oligopeptides, and kynurenine pathway metabolites were significantly correlated with depression severity. This suggests that these metabolites can serve as markers for monitoring ECT efficacy during treatment. Finally, an exploratory analysis was conducted to examine the fluctuations in metabolite levels before and after the first ECT session with the aim of predicting subsequent remission following ECT. The levels of some metabolites such as GABA, cortisol, and 5-HIAA were significantly altered by the first ECT session only in the remission group.

The baseline cortisol level in the patients was higher than that in HCs, suggesting increased activity of the HPA axis. Increased HPA activity and high levels of cortisol are well-documented characteristics of MDD and are expected to reflect the interaction between genes and the environment^12^. The high cortisol levels in the patient group in this study may have resulted from genetic variants possibly carried by the patients or primary/secondary stressful environments triggered by MDD. Furthermore, preliminary observations revealed a two-fold change in cortisol levels before and after the initial ECT session in the remission group, whereas the non-remission group exhibited minimal alterations. The findings of the present study suggest that activation of the HPA axis is required by ECT to achieve the intended therapeutic effect. In other words, a blunted cortisol stress response may be associated with treatment resistance to ECT, just as the blunted cortisol response to psychological stress in MDD^13^. Patients with high cortisol levels may benefit from improving their cortisol rhythm^12^, and manipulation of cortisol levels using dehydroepiandrosterone has shown some success as an antidepressant treatment^14^. Therefore, improving cortisol levels prior to the initiation of ECT may enhance the therapeutic efficacy of ECT.

The current study also demonstrated lower levels of Trp, 5-HIAA, and kynurenine pathway metabolites (including Kyn, 3OH-Kyn, and xanthurenic acid) in patients with MDD than in HCs, which is partly consistent with the results of a meta-analysis in patients with depression^15^. Trp is metabolized to Kyn, 3OH-Kyn, and 3-hydroxyantharanilic acid by tryptophan 2,3-dyoxygenase (TDO) and indoleamine 2,3-dioxygenase. Given that TDO is activated by cortisol^16,17^, the low levels of Trp and kynurenine pathway metabolites in patients with MDD reported in the present study could be explained by TDO activation and consequent increases in kynurenine pathway metabolism caused by elevated cortisol levels. Moreover, longitudinal observations of metabolites in patients with MDD during ECT revealed a positive correlation between the serotonin metabolite 5-HIAA and depressive symptoms. In other words, milder depressive symptoms corresponded to lower 5-HIAA levels. Changes in 5-HIAA with ECT have been reported in the literature; however, the findings have been inconsistent, with lower levels in serum within 2 h of initial ECT compared with those at baseline^18^, higher levels in CSF within 1 week after ECT compared with those at baseline^19^, and no significant change in serum levels during ECT^5^. Possible factors affecting inconsistent results between studies may include the timing of sample collection, the specimens used for each study (i.e., plasma, serum, and CSF), and the history of drug use. Changes in serotonin and 5-HIAA levels caused by antidepressant treatment may be complex, such as an acute drop and subsequent surge. Further studies using multiple assessments during treatment with a variety of specimens are needed.

The current study demonstrated lower baseline levels of GABA in patients with MDD than in HCs and a positive correlation between GABA levels and depression severity in patients with MDD during ECT. The effect of ECT on GABA in the brain is unclear, with one study showing an increase^20^ while two other studies failed to replicate this result^21,22^. Indeed, GABA levels appear to fluctuate dynamically at different time points after ECT. A prospective observational study during ECT reported a significant increase in GABA levels 10 min after the first ECT session^23^ and a significant decrease 2 h later^24^. Although the role of GABA in MDD is not fully understood, GABA levels have the potential to be a trait marker as well as a state marker for MDD during ECT, and dynamic ECT-induced changes in GABA levels may be associated with the therapeutic mechanisms of ECT.

The current study has several limitations. First, considering the small sample size, some metabolite associations may have been overlooked because of type 2 errors. In particular, regarding the comparison of metabolite levels between the remission and non-remission groups, this remains an exploratory analysis, and the findings should be interpreted with caution and confirmed in a larger sample size. Second, as this study was exploratory, we did not perform corrections for multiple tests. Last, peripheral blood is a convenient source of biomarkers for clinical applications; however, further investigation of the respective metabolic pathways may be necessary to determine the extent to which peripheral blood reflects changes in the central nervous system.

## Conclusion

In the present study, we conducted targeted and non-targeted metabolomic analyses to comprehensively evaluate a large number of metabolites. We found that plasma levels of neurotransmitters and their degradation products varied throughout the course of ECT. Specifically, GABA and 5-HIAA can act as companion markers of the ECT response. Moreover, baseline cortisol levels may provide insight into the pathophysiology of MDD. Implementing such metabolome measurement systems could assist physicians in selecting effective treatment strategies, and metabolic markers could be useful for monitoring abnormal brain function and subsequent recovery in patients with MDD.

## Methods

### Participants

The authors declare that all procedures contributing to this work comply with the ethical standards of the relevant national and institutional committees on human experimentation and the Declaration of Helsinki of 1975, as revised in 2008. All procedures involving human participants/patients were approved by the Institutional Review Board of Keio University School of Medicine (Approval No. 20160074). Before participation, written informed consent was obtained from all patients or their proxies and from all HCs participants after providing them with detailed protocol information. Patient competence for consent was evaluated using the MacArthur Competence Assessment Tool for Clinical Research^25^.

Inclusion criteria were age ≥ 20 years; fulfillment of the diagnostic criteria for MDD according to the Diagnostic and Statistical Manual of Mental Disorders, Fifth Edition (DSM-5),^26^ as determined by at least two certified psychiatrists; scheduled for ECT at Keio University Hospital between October 2016 and March 2020; and lack of response to at least two antidepressant medications at therapeutic doses indicated in their packet inserts^27^ or presentation with serious psychiatric conditions that required immediate treatment. Exclusion criteria were the presence of any serious physical illness, history of ECT within the past 6 months, diagnosis of alcohol or substance use disorder according to the DSM-5^26^ within the past 6 months, history of head trauma with significant neurological sequelae, and history of vagal reflex on blood sampling.

An equal number of age- and sex-matched healthy individuals were recruited as HCs. These participants did not meet any criteria for psychiatric disorders according to the DSM-5 and had no history of psychiatric disorders.

### ECT procedure

ECT was performed as previously described^28^. Briefly, patients were treated with bitemporal ECT twice or thrice weekly using a brief pulse (0.5 ms) square-wave ECT device (Thymatron System IV device; Somatics, Inc., Lake Bluff, IL, USA). Treatments were continued until no further improvement was observed in the last two sessions by the psychiatrist in charge, based on clinical observations. Sodium thiopental (3–5 mg/kg), propofol (1 mg/kg), or sevoflurane was used for general anesthesia, and succinylcholine (0.5–1.0 mg/kg) was used to induce muscle relaxation during ECT.

### Specimen collection

Venous blood samples were collected in EDTA-2 K vacuum tubes (Terumo, Tokyo, Japan) before and after the first (T1 and T2, respectively), third (T3 and T4, respectively), and final ECT sessions (T5 and T6, respectively) and 3–7 days after the final ECT session (T7) (Fig. S6A). Blood sampling before the ECT session was performed between 8 and 10 am, within 2 h of the ECT session, in a fasting state. Post-ECT blood sampling was conducted 1–2 h after the ECT session in the fasting state. Blood sampling was avoided within 1 h of the ECT session to reduce the potential anesthetic drug effects. Blood sampling from the HCs was performed between 8 and 10 am in a fasting state. Within 1 h of blood sampling, plasma was separated by centrifugation at 1200× *g* for 10 min at 4 °C using a swing-type centrifugation rotor (Kokusan H-19R, Japan). Plasma was transferred to a microtube and stored at − 80 °C.

### Clinical assessments

Depressive symptoms were assessed using the MADRS^29^ and the Japanese translation of the 16-item QIDS Self-Reported version^30,31^ before the first ECT session (T1), after the third ECT session (T4), after the final ECT session (T6), and 3–7 days after the final ECT session (T7). Remission was defined as a MADRS total score of ≤ 9 after the final ECT session. Additionally, the following information was collected: age; sex; educational history; age at MDD onset; family history of MDD; current smoking status; current drinking status; number of previous depressive episodes; number of previous psychiatric admissions, duration of the present episode; presence of psychotic, melancholic, and catatonic features according to the DSM-5 criteria for depressive disorders^26^; drugs administered at the time of the first ECT; charge used in the first and last ECT sessions; and number of ECT sessions conducted.

### Comprehensive targeted/non-targeted metabolomic analysis of plasma for biomarker discovery

The unique feature of this study was the simultaneous targeted and non-targeted analyses of metabolites in the plasma using two types of chromatography/mass spectrometry. Targeted liquid chromatography–mass spectroscopy (LC–MS/MS) and ion chromatography–MS (IC-MS) were performed on metabolites registered in the lab compound library by comparing the measured data in plasma with reference standards (System-1 and System-2 in Fig. S6B, respectively). Fourier-transform mass spectroscopy was also used for non-targeted analysis, that is, the detection of compounds not registered in the laboratory compound library (System-2 in Fig. S6B). The metabolites identified by chemical formula analysis (exact mass and isotope pattern matching) and structural analysis (MS/MS) were validated by comparison with commercially available reference standard compounds. For reference standards that were unavailable, we provided the compositional formula and name of the candidate compound. Finally, 217 components were identified and validated by targeted analysis, and an additional 140 components were used in subsequent analyses as metabolites that met compositional matching or structural identification criteria by both compositional matching and MS/MS (Fig. S6C). The detailed procedures of the metabolomic analysis are described in the Supplementary Material.

### Statistical analyses

#### Demographic variables

Differences in variables between patients with MDD who achieved remission after ECT (remission group) and those who did not (non-remission group) were determined using independent t-tests for continuous variables and Fisher’s exact test for categorical variables, with a significance level of 0.05.

#### Plasma metabolite levels of MDD and HCs at baseline

PLS-DA was used to provide an overview of the differences in the levels of plasma metabolites obtained from the metabolomic analysis between patients with MDD and HCs. Volcano plot analysis was performed to identify metabolites with significant differences in levels between patients with MDD and HCs. In addition, the levels of metabolites identified by volcano plot analysis were compared between patients with MDD and HCs using Student’s t-test. HCA was performed to extract and list the metabolite cluster characteristics of the two groups.

#### Correlations between plasma metabolite levels and depressive symptoms during ECT

PLS-DA was applied to provide an overview of the longitudinal change in the plasma metabolome over three pre-ECT session time points (T1, T3, and T5) and 3 days after the final ECT session (T7). Subsequently, for each measured metabolite, we performed a single correlation analysis incorporating metabolite levels at T1, T3, T5, and T7 and the corresponding severity of depression, as indicated by the QIDS and MADRS total scores.

#### Plasma metabolite fluctuation by the first ECT session predicts subsequent remission after ECT

PLS-DA was conducted to provide an overview of the differences in metabolite levels between T1 and T2. We then performed a volcano plot analysis to identify metabolites with significant changes in levels in the remission group. Moreover, changes in their levels were compared using Student’s t-test before and after the first ECT session between the remission and non-remission groups.

### Supplementary Information

Supplementary Information.

## Data Availability

The datasets used and/or analyzed during the current study are not publicly available in accordance with the provisions of the ethics committee but are available from the corresponding author upon reasonable request.

## References

[1] Rush AJ (2006). Acute and longer-term outcomes in depressed outpatients requiring one or several treatment steps: a STAR*D report. Am. J. Psychiatry.

[2] UK Ect Review Group (2003). Efficacy and safety of electroconvulsive therapy in depressive disorders: A systematic review and meta-analysis. The Lancet.

[3] Tunvirachaisakul C (2018). Predictors of treatment outcome in depression in later life: A systematic review and meta-analysis. J. Affect Disord..

[4] Schwieler L (2016). Electroconvulsive therapy suppresses the neurotoxic branch of the kynurenine pathway in treatment-resistant depressed patients. J. Neuroinflam..

[5] Guloksuz S (2015). The impact of electroconvulsive therapy on the tryptophan-kynurenine metabolic pathway. Brain Behav. Immun..

[6] Vukadin M, Birkenhager TK, Wierdsma AI, Groenland TH, van den Broek WW (2011). Post-dexamethasone cortisol as a predictor for the efficacy of electroconvulsive therapy in depressed inpatients. J. Psychiatr. Res..

[7] Singh A, Kar SK (2017). How electroconvulsive therapy works?: Understanding the neurobiological mechanisms. Clin. Psychopharmacol. Neurosci..

[8] MacDonald K (2019). Biomarkers for major depressive and bipolar disorders using metabolomics: A systematic review. Am. J. Med Genet. B Neuropsychiatr. Genet..

[9] Stetler C, Miller GE (2011). Depression and hypothalamic-pituitary-adrenal activation: A quantitative summary of four decades of research. Psychosom. Med..

[10] Chen JX (2020). Association of serum uric acid levels with suicide risk in female patients with major depressive disorder: A comparative cross-sectional study. BMC Psychiatry.

[11] Savitz J (2020). The kynurenine pathway: a finger in every pie. Mol. Psychiatry.

[12] Herbert J (2013). Cortisol and depression: Three questions for psychiatry. Psychol. Med..

[13] Burke HM, Davis MC, Otte C, Mohr DC (2005). Depression and cortisol responses to psychological stress: A meta-analysis. Psychoneuroendocrinology.

[14] Rabkin JG, McElhiney MC, Rabkin R, McGrath PJ, Ferrando SJ (2006). Placebo-controlled trial of dehydroepiandrosterone (DHEA) for treatment of nonmajor depression in patients with HIV/AIDS. Am. J. Psychiatry.

[15] Ogyu K (2018). Kynurenine pathway in depression: A systematic review and meta-analysis. Neurosci. Biobehav. Rev..

[16] Fukuda K (2014). Etiological classification of depression based on the enzymes of tryptophan metabolism. BMC Psychiatry.

[17] Nagao M, Nakamura T, Ichihara A (1986). Developmental control of gene expression of tryptophan 2,3-dioxygenase in neonatal rat liver. Biochim. Biophys. Acta.

[18] Hofmann P (1996). 5-Hydroxy-indolacetic-acid (5-HIAA) serum levels in depressive patients and ECT. J. Psychiatry Res..

[19] Nikisch G, Mathé AA (2008). CSF monoamine metabolites and neuropeptides in depressed patients before and after electroconvulsive therapy. Eur. Psychiatry.

[20] Sanacora G (2003). Increased cortical GABA concentrations in depressed patients receiving ECT. Am. J. Psychiatry.

[21] Knudsen MK, Near J, Blicher AB, Videbech P, Blicher JU (2019). Magnetic resonance (MR) spectroscopic measurement of γ-aminobutyric acid (GABA) in major depression before and after electroconvulsive therapy. Acta Neuropsychiatry.

[22] Erchinger VJ (2020). Anterior cingulate gamma-aminobutyric acid concentrations and electroconvulsive therapy. Brain Behav..

[23] Esel E (2008). The effects of electroconvulsive therapy on GABAergic function in major depressive patients. J. ECT.

[24] Palmio J (2005). Changes in plasma amino acids after electroconvulsive therapy of depressed patients. Psychiatry Res..

[25] Appelbaum, P. S. & Grisso, T. MacArthur competence assessment tool for clinical research (MacCAT-CR). in *Professional Resource Press/Professional Resource Exchange* (2001).

[26] American Psychiatric Association (2013). Diagnostic and Statistical Manual of Mental Disorders.

[27] McIntyre RS (2014). Treatment-resistant depression: Definitions, review of the evidence, and algorithmic approach. J. Affect. Disord..

[28] Nakajima K (2022). Individual prediction of remission based on clinical features following electroconvulsive therapy: A machine learning approach. J. Clin. Psychiatry.

[29] Montgomery SA, Asberg M (1979). A new depression scale designed to be sensitive to change. Br. J. Psychiatry J. Mental Sci..

[30] Rush AJ (2003). The 16-Item Quick Inventory of Depressive Symptomatology (QIDS), clinician rating (QIDS-C), and self-report (QIDS-SR): A psychometric evaluation in patients with chronic major depression. Biol. Psychiatry.

[31] Fujisawa D (2010). Cross-cultural adaptation of the Quick inventory of depressive symptomatology, self-report (QIDS-SR-J). Jpn. J. Stress Sci..

