# Supplementary material for: Metabolites for monitoring symptoms and predicting remission in patients with depression who received electroconvulsive therapy: a pilot study
Source: Sci Rep. 2023 Aug 14;13:13218. doi: 10.1038/s41598-023-40498-7 (PMC10425446; doi:10.1038/s41598-023-40498-7)
Supplement: Supplementary file 1 — Supplementary Information. [file 41598_2023_40498_MOESM1_ESM.docx]

**Supplementary materials**

**Metabolites for monitoring symptoms and predicting remission in patients with depression who received electroconvulsive therapy: a pilot study**

| **Table of contents** | **Page no.** |
| --- | --- |

**Supplementary Methods**

| Targeted metabolomics |  | 3 – 5 |
| --- | --- | --- |
| Non-targeted metabolomics |  | 6 |

**Supplementary Tables**

| Table S1 | PLS-DA cross-validation details for Fig. 1A | 7 |
| --- | --- | --- |
| Table S2 | VIP scores for each metabolite calculated for Fig. 1A | 8-19 |
| Table S3 | Metabolites significantly different between patients with depression and HCs at the baseline | 20 |
| Table S4 | VIP scores for each metabolite calculated for Fig. 2B | 21-32 |
| Table S5 | The list of metabolites showed significant positive correlations with MADRS score | 33 |
| Table S6 | The list of metabolites showed significant negative correlations with MADRS score | 34 |
| Table S7 | VIP scores for each metabolite calculated Fig. 3B | 35-46 |
| Table S8 | Metabolites showed significant change by the first ECT in the remission group | 47 |

**Supplementary Tables**

| Figure S1 | PLS-DA cross-validation details for Fig. 1A | 48 |
| --- | --- | --- |
| Figure S2 | HCA to classify the metabolites with increased or decreased plasma levels in patients with MDD compared with in HCs at baseline | 49 |
| Figure S3 | Change in MADRS or QIDS scores during ECT for each patient | 50 |
| Figure S4 | Scatter plots of metabolite showing significant correlations with MADRS and/or QIDS | 51-52 |
| Figure S5 | The metabolites showed significant changes in their levels only in the remission group | 53 |
| Figure S6 | Flow of blood sampling and metabolome measurement | 54-55 |

**Supplementary Methods**

**Targeted metabolomics**

Metabolite extraction from plasma samples for metabolomic analysis was performed as previously described.^1^ Frozen plasma (50 µL) was mixed with methanol (500 µL) and internal standard compounds, followed by the addition of equal volumes of chloroform and a 0.4-volume of ultrapure water (LC/MS grade, Wako, Tokyo, Japan). The suspension was then centrifuged at 2,800 *g* for 15 min at 4°C. After centrifugation, the aqueous phase was ultrafiltered using ultrafiltration tubes (Ultrafree MC-PLHCC; Human Metabolome Technologies, Boston, MA, USA). The filtrate was concentrated using a vacuum concentrator (SpeedVac, Thermo Fisher Scientific, Waltham, MA, USA). The concentrated filtrate was dissolved in 50 μL ultrapure water and used for ion chromatography-Fourier transform mass spectrometry (IC-FTMS) and liquid chromatography-tandem mass spectrometry (LC-MS/MS) analyses. Internal standards were added to tissues prior to extraction. The internal standard for anionic metabolites, 2-morpholinoethanesulfonic acid, was used as the ideal standard because it is not present in tissues. Loss of endogenous metabolites during sample preparation was corrected by calculating the recovery (%) of each sample measurement. Plasma samples were pretreated once and assayed serially. Compounds with mainly positively and negatively charged functional groups were separated using two types of chromatography, IC and LC (PFP columns), respectively, with high resolution.

*IC-FTMS for anionic metabolites*: For metabolomic analysis focused on central glucose metabolic pathways, namely, glycolysis, the tricarboxylic acid cycle, and the pentose phosphate pathway, anionic metabolites were measured using an orbitrap-type mass spectrometer (Q-Exactive focus, Thermo Fisher Scientific) connected to a high-performance IC system (ICS-5000+, Thermo Fisher Scientific), which enabled highly selective and sensitive metabolite quantification due to the IC separation and Fourier transform mass spectrometry principle. The ion chromatograph was equipped with an anion electrolytic suppressor (Thermo Scientific Dionex AERS 500) to convert the potassium hydroxide gradient into pure water before the sample entered the mass spectrometer. Separation was performed using a Thermo Scientific Dionex IonPac AS11-HC, 4-μm particle size column. The IC flow rate was 0.25 mL/min, with post-column supplementation with a 0.18-mL/min makeup flow of MeOH. The potassium hydroxide gradient conditions for IC separation were as follows: 1–100 mM (0–40 min), 100 mM (40–50 min), and 1 mM (50.1–60 min) at a column temperature of 30°C. The Q-Exactive focus mass spectrometer was operated in ESI negative mode for all detections. Full mass scanning (*m/z* 70–900) was performed at a resolution of 70,000. The automatic gain control (AGC) target was set at 3 × 10^6^ ions, and the maximum ion injection time was 100 ms. The source ionization parameters were optimized at a 3-kV spray voltage, transfer temperature of 320°C; S-Lens level of 50, heater temperature of 300°C; sheath gas at 36, and Aux gas at 10.

*LC-FTMS for cationic metabolites*: For non-targeted analysis, the mass spectrometer (Q-Exactive focus, Thermo Fisher Scientific) was connected to a high-performance liquid chromatograph (Ultimate3000 system, Thermo Fisher Scientific). The column and separation conditions were the same as those used for the targeted amino acid measurements. The Q-Exactive focus mass spectrometer was operated in ESI positive mode for all detections. A full mass scan (*m/z* 50−900), followed by three rapid data-dependent MS/MS analyses, was performed at a resolution of 70,000. The AGC target was set at 3 × 10^6^ ions, and the maximum ion injection time (IT) was 100 ms. Source ionization parameters were optimized at the same settings as for anionic metabolites.

*LC-MS/MS for cationic metabolites*: The cationic metabolite (amino acids) levels in patient plasma were quantified using liquid chromatography-tandem mass spectrometry (LC-MS/MS). A triple-quadrupole mass spectrometer equipped with an ESI ion source (LCMS-8040, Shimadzu Corporation, Kyoto, Japan) was used in positive and negative ESI and multiple reaction monitoring (MRM) modes. Samples were resolved on a Discovery HS F5-3 column (2.1 mm ID × 150 mm L, 3-μm particle, Sigma-Aldrich, St Louis, MO, USA) using a step-gradient with mobile phase A (0.1% formate) and mobile phase B (0.1% acetonitrile) at ratios of 100:0 (0–5 min), 75:25 (5–11 min), 65:35 (11–15 min), 5:95 (15–20 min), and 100:0 (20–25 min), at a flow rate of 0.25 mL/min and a column temperature of 40°C. The MRM conditions for each amino acid have been described previously.^2^

1. Miyajima M, Zhang B, Sugiura Y, Sonomura K, Guerrini MM, Tsutsui Y, et al. Metabolic shift induced by systemic activation of T cells in PD-1-deficient mice perturbs brain monoamines and emotional behavior. Nat Immunol. 2017;**18**(12):1342-52.

2. Oka M, Hashimoto K, Yamaguchi Y, Saitoh SI, Sugiura Y, Motoi Y, et al. Arl8b is required for lysosomal degradation of maternal proteins in the visceral yolk sac endoderm of mouse embryos. J Cell Sci. 2017;**130**(20):3568-77.

**Non-targeted metabolome analysis**

Compound Discoverer 3.2 (Thermo Fisher Scientific) was used for the non-targeted metabolomics workflow, as described by Zhou et al.^1^ This software first aligned the total ion chromatograms of the different samples along the retention time-course. Then, features detected with an intensity ≥100,000 and a signal-to-noise ratio >5 in each dataset were extracted and merged into components. The resulting compounds were identified by both (i) formula prediction, based on accurate m/z values and isotope peak patterns, and (ii) MS/MS structural validation. Moreover, formula-predicted signals were assigned to candidate compounds by a database search (Chemspider database; http://www.chemspider.com/). For molecules for which reference compounds were available for purchase, retention time matches were checked after acquisition. Those for which references were not available were described by compositional formula and the name of the candidate compound was listed alongside.

1. Zhou Z. Non-target impurity profiling of marketplace Cetirizine using high-resolution mass spectrometry and multivariate data analysis. Rapid Commun Mass Spectrom. 2016;**30**(17):1941-50.

**Supplementary table S1. PLS-DA cross-validation details for Fig. 1A**

| Measure | 1 comps | 2 comps | 3 comps | 4 comps | 5 comps |
| --- | --- | --- | --- | --- | --- |
| Accuracy | 0.86 | 0.91 | 0.92 | 0.92 | 0.92 |
| R2 | 0.84843 | 0.92431 | 0.97377 | 0.99256 | 0.99871 |
| Q2 | 0.58946 | 0.69571 | 0.69975 | 0.72179 | 0.72248 |

Supplementary table S2. VIP scores for each metabolite calculated for Fig. 1A

|  | Comp. 1 | Comp. 2 | Comp. 3 | Comp. 4 | Comp. 5 | Comp. 6 | Comp. 7 | Comp. 8 |
| --- | --- | --- | --- | --- | --- | --- | --- | --- |
| (7S_8S)-DiHODE [C18 H32 O4] | 2.41 | 2.31 | 2.25 | 2.23 | 2.22 | 2.22 | 2.22 | 2.22 |
| gamma-Glu-Glu | 2.32 | 2.22 | 2.17 | 2.16 | 2.16 | 2.15 | 2.15 | 2.15 |
| L-Norleucine [C6 H13 N O2] | 2.30 | 2.20 | 2.15 | 2.13 | 2.12 | 2.12 | 2.12 | 2.12 |
| KKK [C18 H38 N6 O4] | 2.21 | 2.13 | 2.08 | 2.07 | 2.07 | 2.07 | 2.07 | 2.07 |
| Thymidine | 2.09 | 2.04 | 1.99 | 1.97 | 1.96 | 1.96 | 1.96 | 1.96 |
| n-heptanoic acid [C7 H14 O2] | 2.07 | 1.98 | 1.95 | 1.93 | 1.93 | 1.93 | 1.93 | 1.93 |
| Xanthurenic acid | 2.04 | 1.96 | 1.92 | 1.90 | 1.90 | 1.89 | 1.89 | 1.89 |
| gamma-Glu-Trp | 2.03 | 2.03 | 1.98 | 1.97 | 1.96 | 1.96 | 1.96 | 1.96 |
| Adenine | 1.99 | 1.91 | 1.87 | 1.85 | 1.85 | 1.85 | 1.85 | 1.85 |
| Butylbutyrate [C8 H16 O2] | 1.97 | 1.89 | 1.84 | 1.83 | 1.82 | 1.82 | 1.82 | 1.82 |
| Theobromine [C7 H8 N4 O2] | 1.95 | 1.88 | 1.83 | 1.82 | 1.81 | 1.81 | 1.81 | 1.81 |
| Kynurenine | 1.95 | 1.90 | 1.87 | 1.85 | 1.84 | 1.84 | 1.84 | 1.84 |
| L-Norepinephrine | 1.93 | 1.85 | 1.81 | 1.79 | 1.79 | 1.79 | 1.79 | 1.79 |
| Hexadecanamide [C16 H33 N O] | 1.91 | 1.83 | 1.78 | 1.77 | 1.77 | 1.77 | 1.77 | 1.77 |
| N-Acetylaspartate | 1.90 | 1.84 | 1.80 | 1.78 | 1.77 | 1.77 | 1.77 | 1.77 |
| Cortisol | 1.87 | 1.80 | 1.76 | 1.74 | 1.74 | 1.74 | 1.74 | 1.74 |
| Cytosine | 1.85 | 1.85 | 1.81 | 1.79 | 1.79 | 1.79 | 1.79 | 1.79 |
| 4-Methyl-2-oxopentanoate | 1.85 | 1.80 | 1.79 | 1.78 | 1.77 | 1.77 | 1.77 | 1.77 |
| Pyrophosphate | 1.85 | 1.80 | 1.76 | 1.75 | 1.75 | 1.75 | 1.74 | 1.74 |
| Glucose 1-Phosphate | 1.84 | 1.78 | 1.78 | 1.79 | 1.79 | 1.78 | 1.78 | 1.78 |
| 2,3-Dihydroxybenzoate [C7 H6 O4] | 1.82 | 1.74 | 1.71 | 1.69 | 1.69 | 1.69 | 1.69 | 1.69 |
| Paraxanthine [C7 H8 N4 O2] | 1.82 | 1.74 | 1.71 | 1.69 | 1.69 | 1.69 | 1.69 | 1.69 |
| Ile | 1.80 | 1.73 | 1.68 | 1.67 | 1.66 | 1.66 | 1.66 | 1.66 |
| Alanine | 1.79 | 1.72 | 1.68 | 1.66 | 1.66 | 1.66 | 1.66 | 1.66 |
| Decanamide [C10 H21 N O] | 1.79 | 1.72 | 1.68 | 1.66 | 1.66 | 1.66 | 1.66 | 1.66 |
| 4-Hydroxyphenyllactic acid [C9 H10 O4] | 1.77 | 1.72 | 1.68 | 1.66 | 1.66 | 1.66 | 1.66 | 1.66 |
| Gentisic acid [C7 H6 O4] | 1.77 | 1.70 | 1.68 | 1.67 | 1.66 | 1.66 | 1.66 | 1.66 |
| Di-hexose | 1.77 | 1.74 | 1.70 | 1.68 | 1.68 | 1.68 | 1.68 | 1.68 |
| Stearamide [C18 H37 N O] | 1.74 | 1.67 | 1.63 | 1.62 | 1.62 | 1.62 | 1.62 | 1.62 |
| Fatty acid (C6:0) [C6 H12 O2] | 1.74 | 1.66 | 1.63 | 1.61 | 1.61 | 1.61 | 1.61 | 1.61 |
| 5α-Dihydrotestosterone [C19 H30 O2] | 1.74 | 1.68 | 1.67 | 1.65 | 1.65 | 1.65 | 1.65 | 1.65 |
| 5-Hydroxyindole-3-acetic acid | 1.73 | 1.69 | 1.67 | 1.65 | 1.65 | 1.65 | 1.65 | 1.65 |
| Urea | 1.69 | 1.66 | 1.71 | 1.69 | 1.69 | 1.69 | 1.69 | 1.69 |
| Tryp | 1.67 | 1.64 | 1.60 | 1.59 | 1.58 | 1.58 | 1.58 | 1.58 |
| cAMP | 1.67 | 1.65 | 1.61 | 1.60 | 1.60 | 1.59 | 1.59 | 1.59 |
| N-Acetylneuraminic_acid | 1.66 | 1.62 | 1.59 | 1.58 | 1.57 | 1.57 | 1.57 | 1.57 |
| Palmitoleic acid [C16 H30 O2] | 1.66 | 1.64 | 1.62 | 1.61 | 1.60 | 1.60 | 1.60 | 1.60 |
| Quinaldic acid | 1.66 | 1.66 | 1.62 | 1.62 | 1.61 | 1.61 | 1.61 | 1.61 |
| 3-Hydroxy-DL-kynurenine | 1.63 | 1.61 | 1.57 | 1.56 | 1.56 | 1.56 | 1.56 | 1.56 |
| N3,N4-Dimethyl-L-arginine [C8 H18 N4 O2] | 1.61 | 1.58 | 1.54 | 1.53 | 1.52 | 1.52 | 1.52 | 1.52 |
| 2-Oxobutyrate | 1.59 | 1.53 | 1.51 | 1.49 | 1.49 | 1.49 | 1.49 | 1.49 |
| N6-Me-Adenosine [C11 H15 N5 O4] | 1.57 | 1.54 | 1.52 | 1.51 | 1.51 | 1.51 | 1.51 | 1.51 |
| His | 1.55 | 1.50 | 1.47 | 1.46 | 1.45 | 1.45 | 1.45 | 1.45 |
| Phe | 1.54 | 1.51 | 1.47 | 1.46 | 1.46 | 1.46 | 1.46 | 1.46 |
| beta-Hydroxybutyrate | 1.54 | 1.48 | 1.46 | 1.45 | 1.45 | 1.44 | 1.44 | 1.44 |
| 2-Hydroxyisovaleric acid [C5 H10 O3] | 1.53 | 1.48 | 1.47 | 1.45 | 1.45 | 1.45 | 1.45 | 1.45 |
| GT [C6 H12 N2 O4] | 1.53 | 1.49 | 1.46 | 1.46 | 1.46 | 1.46 | 1.46 | 1.46 |
| Octylphosphonic acid [C8 H19 O3 P] | 1.51 | 1.45 | 1.47 | 1.46 | 1.45 | 1.45 | 1.45 | 1.45 |
| gamma-Glu-Arg | 1.51 | 1.45 | 1.46 | 1.45 | 1.45 | 1.45 | 1.45 | 1.45 |
| LKKK [C24 H49 N7 O5] | 1.51 | 1.49 | 1.45 | 1.44 | 1.44 | 1.43 | 1.43 | 1.43 |
| ADP-ribose | 1.50 | 1.46 | 1.43 | 1.42 | 1.43 | 1.43 | 1.43 | 1.43 |
| Succinate | 1.49 | 1.43 | 1.40 | 1.38 | 1.38 | 1.38 | 1.38 | 1.38 |
| Dimethylglycine | 1.48 | 1.42 | 1.41 | 1.39 | 1.39 | 1.39 | 1.39 | 1.39 |
| gamma-Glu-Phe | 1.48 | 1.44 | 1.43 | 1.42 | 1.42 | 1.42 | 1.42 | 1.42 |
| Val | 1.48 | 1.45 | 1.41 | 1.40 | 1.40 | 1.40 | 1.40 | 1.40 |
| GTKK [C18 H36 N6 O6] | 1.48 | 1.43 | 1.41 | 1.39 | 1.39 | 1.39 | 1.39 | 1.39 |
| Uric_acid | 1.45 | 1.42 | 1.39 | 1.38 | 1.37 | 1.37 | 1.37 | 1.37 |
| Hydroxyproline | 1.44 | 1.40 | 1.37 | 1.35 | 1.35 | 1.35 | 1.35 | 1.35 |
| Pyroglutamic acid | 1.43 | 1.37 | 1.34 | 1.33 | 1.33 | 1.33 | 1.33 | 1.33 |
| Uracil | 1.42 | 1.36 | 1.39 | 1.38 | 1.38 | 1.38 | 1.38 | 1.38 |
| Ophthalmic Acid | 1.42 | 1.37 | 1.35 | 1.34 | 1.33 | 1.33 | 1.33 | 1.33 |
| Fumarate | 1.42 | 1.40 | 1.37 | 1.36 | 1.36 | 1.36 | 1.36 | 1.36 |
| Aica ribonucleotide | 1.41 | 1.36 | 1.35 | 1.33 | 1.33 | 1.33 | 1.33 | 1.33 |
| Azelaic acid [C9 H16 O4] | 1.39 | 1.40 | 1.36 | 1.35 | 1.35 | 1.35 | 1.35 | 1.35 |
| Tyr | 1.38 | 1.35 | 1.33 | 1.33 | 1.33 | 1.33 | 1.33 | 1.33 |
| gamma-Glu-His | 1.37 | 1.31 | 1.28 | 1.27 | 1.27 | 1.27 | 1.27 | 1.27 |
| Glycerol 2-phosphate | 1.37 | 1.31 | 1.30 | 1.29 | 1.29 | 1.29 | 1.29 | 1.29 |
| beta-HMB (Hydroxy-methylbutyrate) | 1.36 | 1.35 | 1.34 | 1.33 | 1.33 | 1.33 | 1.33 | 1.33 |
| 2-Aminonicotinic acid [C6 H6 N2 O2] | 1.36 | 1.33 | 1.31 | 1.31 | 1.31 | 1.31 | 1.31 | 1.31 |
| Pyruvate | 1.36 | 1.36 | 1.33 | 1.32 | 1.32 | 1.32 | 1.32 | 1.32 |
| Cystathionine | 1.34 | 1.30 | 1.27 | 1.26 | 1.26 | 1.26 | 1.26 | 1.26 |
| Asp | 1.32 | 1.30 | 1.28 | 1.27 | 1.26 | 1.26 | 1.26 | 1.26 |
| Creatinine | 1.32 | 1.26 | 1.25 | 1.24 | 1.23 | 1.23 | 1.23 | 1.23 |
| Ornithine | 1.31 | 1.27 | 1.23 | 1.22 | 1.22 | 1.22 | 1.22 | 1.22 |
| Nicotinamide_[Negative] | 1.30 | 1.25 | 1.21 | 1.20 | 1.20 | 1.20 | 1.20 | 1.20 |
| Pyridoxine | 1.30 | 1.25 | 1.23 | 1.23 | 1.23 | 1.23 | 1.23 | 1.23 |
| Choline | 1.28 | 1.23 | 1.23 | 1.22 | 1.22 | 1.22 | 1.22 | 1.22 |
| Acetyl-glycine [C4 H7 N O3] | 1.28 | 1.23 | 1.22 | 1.21 | 1.21 | 1.21 | 1.21 | 1.21 |
| gamma-Glu-Val | 1.27 | 1.23 | 1.23 | 1.23 | 1.22 | 1.22 | 1.22 | 1.22 |
| Riboflavin-5'-phosphate | 1.27 | 1.33 | 1.31 | 1.29 | 1.29 | 1.29 | 1.29 | 1.29 |
| Ethyl palmitoleate [C18 H34 O2] | 1.27 | 1.33 | 1.30 | 1.29 | 1.29 | 1.29 | 1.29 | 1.29 |
| Fructose 6-Phosphate | 1.26 | 1.22 | 1.21 | 1.20 | 1.20 | 1.20 | 1.20 | 1.20 |
| Adenosine | 1.26 | 1.21 | 1.18 | 1.18 | 1.20 | 1.20 | 1.20 | 1.20 |
| 2-Succinyl cysteine | 1.25 | 1.25 | 1.24 | 1.23 | 1.22 | 1.22 | 1.22 | 1.22 |
| Cystine | 1.24 | 1.22 | 1.20 | 1.18 | 1.18 | 1.18 | 1.18 | 1.18 |
| Norepinephrine | 1.24 | 1.19 | 1.23 | 1.22 | 1.22 | 1.22 | 1.21 | 1.21 |
| Dihydrouracil | 1.22 | 1.17 | 1.21 | 1.20 | 1.20 | 1.20 | 1.20 | 1.20 |
| 3-Indoleacetic acid | 1.20 | 1.20 | 1.18 | 1.18 | 1.18 | 1.18 | 1.18 | 1.18 |
| Acetyl carnitine | 1.19 | 1.18 | 1.15 | 1.14 | 1.14 | 1.14 | 1.14 | 1.14 |
| Sarcosine | 1.18 | 1.18 | 1.18 | 1.17 | 1.17 | 1.17 | 1.17 | 1.17 |
| gamma-Glu-Cys | 1.17 | 1.19 | 1.16 | 1.16 | 1.16 | 1.16 | 1.16 | 1.16 |
| Malate | 1.17 | 1.16 | 1.15 | 1.14 | 1.14 | 1.14 | 1.14 | 1.14 |
| Thiosulfate | 1.17 | 1.12 | 1.09 | 1.08 | 1.08 | 1.08 | 1.08 | 1.08 |
| Creatine_phosphate | 1.17 | 1.17 | 1.15 | 1.14 | 1.14 | 1.14 | 1.14 | 1.14 |
| gamma-Glu-Ser | 1.17 | 1.13 | 1.12 | 1.11 | 1.12 | 1.12 | 1.12 | 1.12 |
| Ascorbic acid 2-sulfate [tentative] | 1.16 | 1.16 | 1.13 | 1.12 | 1.12 | 1.12 | 1.12 | 1.12 |
| Carnitine | 1.15 | 1.10 | 1.14 | 1.13 | 1.13 | 1.12 | 1.12 | 1.12 |
| gamma-Glu-Gln | 1.13 | 1.14 | 1.14 | 1.13 | 1.13 | 1.13 | 1.13 | 1.13 |
| keto-isocaproic acid | 1.13 | 1.12 | 1.10 | 1.09 | 1.09 | 1.08 | 1.09 | 1.09 |
| Gly | 1.13 | 1.15 | 1.12 | 1.12 | 1.11 | 1.11 | 1.11 | 1.11 |
| Uridine | 1.12 | 1.08 | 1.06 | 1.05 | 1.05 | 1.05 | 1.05 | 1.05 |
| gamma-Glu-Gly | 1.11 | 1.07 | 1.14 | 1.16 | 1.15 | 1.15 | 1.15 | 1.15 |
| 9-Oxo-ODE [C18 H30 O3] | 1.10 | 1.05 | 1.03 | 1.06 | 1.06 | 1.06 | 1.06 | 1.06 |
| Hypotaurine | 1.09 | 1.08 | 1.09 | 1.08 | 1.09 | 1.09 | 1.09 | 1.09 |
| tetranor-12(R)-HETE [C16 H26 O3] | 1.09 | 1.13 | 1.10 | 1.12 | 1.11 | 1.11 | 1.11 | 1.11 |
| Dihydroorotate | 1.08 | 1.09 | 1.16 | 1.22 | 1.22 | 1.22 | 1.22 | 1.22 |
| Itaconic acid | 1.07 | 1.08 | 1.06 | 1.09 | 1.09 | 1.09 | 1.09 | 1.09 |
| Tartaric acid [C4 H6 O6] | 1.05 | 1.06 | 1.04 | 1.03 | 1.03 | 1.03 | 1.03 | 1.03 |
| 4-Hydroxybenzoic acid [C7 H6 O3] | 1.05 | 1.09 | 1.17 | 1.16 | 1.16 | 1.16 | 1.16 | 1.16 |
| 2-Keto-4-methylthiobutyric_acid | 1.05 | 1.01 | 1.09 | 1.08 | 1.08 | 1.08 | 1.08 | 1.08 |
| Hexanoylcarnitine [C13 H25 N O4] | 1.05 | 1.04 | 1.02 | 1.06 | 1.05 | 1.05 | 1.05 | 1.05 |
| Eicosapentaenoic acid [C20 H30 O2] | 1.03 | 1.04 | 1.04 | 1.04 | 1.03 | 1.03 | 1.03 | 1.03 |
| S-Adenosylmethionine | 1.03 | 1.00 | 1.04 | 1.03 | 1.03 | 1.03 | 1.03 | 1.03 |
| Carnosine | 1.03 | 1.04 | 1.02 | 1.02 | 1.02 | 1.02 | 1.02 | 1.02 |
| Norophthalmic acid | 1.02 | 1.08 | 1.06 | 1.06 | 1.06 | 1.06 | 1.06 | 1.06 |
| D-Glyceric_acid | 1.02 | 1.07 | 1.05 | 1.04 | 1.04 | 1.04 | 1.04 | 1.04 |
| 2-Isopropylmalic acid [C7 H12 O5] | 1.02 | 0.98 | 0.98 | 0.97 | 0.97 | 0.97 | 0.97 | 0.97 |
| Glucose 6-Phosphate | 1.02 | 1.02 | 0.99 | 1.01 | 1.02 | 1.02 | 1.02 | 1.02 |
| 5-Keto-gluconate [C6 H10 O7] | 1.01 | 1.07 | 1.04 | 1.03 | 1.03 | 1.03 | 1.03 | 1.03 |
| 4-Aminobutyric acid | 1.00 | 0.96 | 0.98 | 0.97 | 0.97 | 0.97 | 0.97 | 0.97 |
| Shikimic acid [C7 H10 O5] | 1.00 | 1.03 | 1.02 | 1.04 | 1.04 | 1.04 | 1.04 | 1.04 |
| 2-Hydroxyhippuric acid [C9 H9 N O4] | 0.99 | 1.11 | 1.09 | 1.09 | 1.09 | 1.09 | 1.09 | 1.09 |
| Dehydroepiandrosterone (DHEA) [C19 H28 O2] | 0.99 | 0.95 | 0.93 | 0.94 | 0.95 | 0.95 | 0.95 | 0.95 |
| Adenylsuccinic acid | 0.98 | 0.95 | 0.93 | 0.92 | 0.92 | 0.92 | 0.92 | 0.92 |
| Lactate | 0.98 | 1.02 | 0.99 | 0.98 | 0.98 | 0.98 | 0.98 | 0.98 |
| formylkynurenine | 0.98 | 0.94 | 0.94 | 0.93 | 0.93 | 0.93 | 0.93 | 0.93 |
| Deoxyuridine [C9 H12 N2 O5] | 0.97 | 0.95 | 1.02 | 1.01 | 1.01 | 1.01 | 1.01 | 1.01 |
| Ribose5-phosphate + Ribulose5-phosphate | 0.97 | 0.93 | 0.92 | 0.91 | 0.92 | 0.92 | 0.92 | 0.92 |
| Benzoic acid [C7 H6 O2] | 0.97 | 0.93 | 0.94 | 0.93 | 0.93 | 0.93 | 0.93 | 0.93 |
| Homocysteic acid | 0.95 | 0.98 | 1.00 | 0.99 | 0.99 | 0.99 | 0.99 | 0.99 |
| 2-Methyl-3-Hydroxybutyric acid [C5 H10 O3] | 0.93 | 0.92 | 0.91 | 0.90 | 0.91 | 0.91 | 0.91 | 0.91 |
| 2-oxoglutarate | 0.93 | 0.90 | 0.90 | 0.89 | 0.90 | 0.90 | 0.90 | 0.90 |
| alpha-keto-butyrate | 0.93 | 0.91 | 0.89 | 0.89 | 0.89 | 0.89 | 0.89 | 0.89 |
| Sedoheptulose_7-phosphate | 0.93 | 0.99 | 0.97 | 0.98 | 0.99 | 0.99 | 0.99 | 0.99 |
| Citrate | 0.93 | 1.06 | 1.03 | 1.03 | 1.02 | 1.02 | 1.02 | 1.02 |
| ADP-ribose 2'-phosphate [C15 H24 N5 O17 P3] | 0.93 | 0.89 | 0.92 | 0.92 | 0.92 | 0.92 | 0.92 | 0.92 |
| Dmethyl-Lys | 0.92 | 0.88 | 0.91 | 0.91 | 0.91 | 0.91 | 0.91 | 0.91 |
| alpha-Keto-iso-valeric acid | 0.92 | 0.94 | 0.92 | 0.92 | 0.92 | 0.92 | 0.92 | 0.92 |
| GDP | 0.91 | 0.93 | 0.93 | 0.93 | 0.94 | 0.94 | 0.94 | 0.94 |
| 2,6-Pyridinedicarboxylic acid [C7 H5 N O4] | 0.91 | 0.91 | 0.90 | 0.89 | 0.89 | 0.89 | 0.89 | 0.89 |
| Quinolinate | 0.91 | 0.91 | 0.90 | 0.89 | 0.89 | 0.89 | 0.89 | 0.89 |
| Erythose [C4 H8 O4] | 0.91 | 0.87 | 0.85 | 0.85 | 0.85 | 0.85 | 0.85 | 0.85 |
| 2-Deoxy-glucose_6-phosphate | 0.90 | 0.88 | 0.88 | 0.87 | 0.87 | 0.87 | 0.87 | 0.87 |
| Docosapentaenoic acid [C22 H34 O2] | 0.90 | 1.06 | 1.04 | 1.05 | 1.04 | 1.04 | 1.04 | 1.04 |
| UTP | 0.90 | 0.92 | 0.91 | 0.95 | 0.95 | 0.95 | 0.95 | 0.95 |
| Homoserine | 0.89 | 0.89 | 0.93 | 0.92 | 0.92 | 0.92 | 0.92 | 0.92 |
| Thr | 0.89 | 0.89 | 0.93 | 0.92 | 0.92 | 0.92 | 0.92 | 0.92 |
| 2-Aminobutyric acid | 0.89 | 0.86 | 0.83 | 0.83 | 0.83 | 0.83 | 0.83 | 0.83 |
| CTP | 0.88 | 0.91 | 0.89 | 0.93 | 0.94 | 0.94 | 0.94 | 0.94 |
| Hippuric acid | 0.88 | 0.99 | 0.97 | 0.98 | 0.99 | 0.99 | 0.99 | 0.99 |
| 3-Methyladipic acid [C7 H12 O4] | 0.88 | 0.86 | 0.89 | 0.89 | 0.89 | 0.89 | 0.89 | 0.89 |
| Pantothenic acid | 0.88 | 0.85 | 0.84 | 0.83 | 0.83 | 0.83 | 0.83 | 0.83 |
| Glutaric acid [C5 H8 O4] | 0.87 | 0.90 | 0.88 | 0.87 | 0.88 | 0.88 | 0.88 | 0.88 |
| GTP | 0.86 | 0.88 | 0.87 | 0.91 | 0.92 | 0.92 | 0.92 | 0.92 |
| Picolinic acid | 0.86 | 0.87 | 0.86 | 0.85 | 0.85 | 0.85 | 0.85 | 0.85 |
| Nicotinic_Acid | 0.86 | 0.87 | 0.85 | 0.85 | 0.85 | 0.85 | 0.85 | 0.85 |
| cyclic FMN [C17 H19 N4 O8 P] | 0.86 | 0.82 | 0.89 | 0.88 | 0.88 | 0.88 | 0.88 | 0.88 |
| Furoic acid | 0.86 | 1.01 | 0.98 | 0.97 | 0.97 | 0.97 | 0.97 | 0.97 |
| O-Acetyl-L-serine | 0.85 | 0.88 | 0.85 | 0.85 | 0.86 | 0.86 | 0.86 | 0.86 |
| Asn | 0.85 | 0.86 | 0.89 | 0.89 | 0.88 | 0.88 | 0.88 | 0.88 |
| Gly-Gly | 0.85 | 0.86 | 0.89 | 0.89 | 0.88 | 0.88 | 0.88 | 0.88 |
| 3-Methyl-2-oxobutanoate | 0.84 | 0.84 | 0.89 | 0.89 | 0.89 | 0.89 | 0.89 | 0.89 |
| Caffeine | 0.84 | 0.86 | 0.87 | 0.88 | 0.88 | 0.88 | 0.88 | 0.88 |
| L-Cysteine_S-sulfate | 0.83 | 0.81 | 0.80 | 0.79 | 0.79 | 0.79 | 0.79 | 0.79 |
| Cys-Gly | 0.83 | 0.80 | 0.79 | 0.80 | 0.80 | 0.80 | 0.80 | 0.80 |
| Vitamine B2 | 0.83 | 0.80 | 0.81 | 0.81 | 0.82 | 0.82 | 0.82 | 0.82 |
| Gly-Leu | 0.82 | 0.83 | 0.81 | 0.82 | 0.82 | 0.82 | 0.82 | 0.82 |
| Orthophosphate | 0.81 | 0.78 | 0.76 | 0.76 | 0.75 | 0.75 | 0.76 | 0.76 |
| gamma-Glu-Tyr | 0.81 | 0.86 | 0.85 | 0.84 | 0.84 | 0.84 | 0.84 | 0.84 |
| Pimelic acid [C7 H12 O4] | 0.81 | 0.89 | 0.87 | 0.86 | 0.86 | 0.86 | 0.86 | 0.86 |
| Butyrate | 0.80 | 0.84 | 0.86 | 0.86 | 0.86 | 0.86 | 0.86 | 0.86 |
| Cysteic_acid | 0.79 | 0.77 | 0.80 | 0.82 | 0.82 | 0.82 | 0.82 | 0.82 |
| beta-Ala | 0.79 | 0.93 | 0.97 | 0.97 | 0.96 | 0.96 | 0.96 | 0.96 |
| Phenylpyruvic acid [C9 H8 O3] | 0.78 | 0.82 | 0.83 | 0.84 | 0.85 | 0.84 | 0.85 | 0.85 |
| Methyl-Lys | 0.78 | 0.78 | 0.76 | 0.76 | 0.76 | 0.76 | 0.76 | 0.76 |
| ATP | 0.78 | 0.79 | 0.80 | 0.84 | 0.85 | 0.85 | 0.85 | 0.85 |
| Oxomalonate [C3 H2 O5] | 0.77 | 0.74 | 0.79 | 0.78 | 0.78 | 0.78 | 0.78 | 0.78 |
| Guanosine | 0.76 | 0.73 | 0.75 | 0.78 | 0.78 | 0.78 | 0.78 | 0.78 |
| Fucose/Rhamnose [C6 H12 O5] | 0.76 | 0.73 | 0.73 | 0.73 | 0.73 | 0.73 | 0.73 | 0.73 |
| beta-hydroxy-iso-butyrate | 0.76 | 0.84 | 0.85 | 0.84 | 0.84 | 0.84 | 0.84 | 0.84 |
| Valproic acid/2-Ethylhexanoic acid [C8 H16 O2] | 0.75 | 0.74 | 0.72 | 0.72 | 0.71 | 0.71 | 0.71 | 0.71 |
| Citrulline | 0.75 | 0.75 | 0.88 | 0.87 | 0.87 | 0.87 | 0.87 | 0.87 |
| Choline.1 | 0.75 | 0.73 | 0.71 | 0.71 | 0.71 | 0.71 | 0.71 | 0.71 |
| Indole-3-lactic acid [C11 H11 N O3] | 0.74 | 0.74 | 0.79 | 0.82 | 0.81 | 0.81 | 0.81 | 0.81 |
| Leucinic acid [C6 H12 O3] | 0.74 | 0.72 | 0.71 | 0.70 | 0.70 | 0.70 | 0.70 | 0.70 |
| 12-Oxo phytodienoic acid [C18 H28 O3] | 0.73 | 0.78 | 0.76 | 0.77 | 0.79 | 0.79 | 0.79 | 0.79 |
| Mandelic acid [C8 H8 O3] | 0.73 | 0.88 | 0.86 | 0.90 | 0.91 | 0.91 | 0.91 | 0.91 |
| Hypoxanthine | 0.72 | 0.70 | 0.70 | 0.70 | 0.70 | 0.70 | 0.70 | 0.70 |
| Citraconic acid | 0.71 | 0.86 | 0.84 | 0.84 | 0.84 | 0.84 | 0.84 | 0.84 |
| Ferulic acid [C10 H10 O4] | 0.71 | 0.68 | 0.79 | 0.79 | 0.80 | 0.80 | 0.80 | 0.79 |
| H4P3O10 | 0.71 | 0.75 | 0.73 | 0.80 | 0.80 | 0.80 | 0.80 | 0.80 |
| L-Carnitine | 0.71 | 0.68 | 0.70 | 0.69 | 0.69 | 0.69 | 0.69 | 0.69 |
| Carbamoyl-DL-aspartic_acid | 0.71 | 0.68 | 0.70 | 0.70 | 0.70 | 0.70 | 0.70 | 0.70 |
| 3-Methyl-L-Histidine | 0.71 | 0.68 | 0.67 | 0.66 | 0.66 | 0.66 | 0.66 | 0.66 |
| Urocanic acid | 0.71 | 0.68 | 0.72 | 0.72 | 0.72 | 0.72 | 0.72 | 0.72 |
| Maltitol/Lactitol [C12 H24 O11] | 0.71 | 0.68 | 0.75 | 0.74 | 0.75 | 0.75 | 0.75 | 0.75 |
| GMP | 0.71 | 0.76 | 0.74 | 0.76 | 0.78 | 0.78 | 0.78 | 0.78 |
| 5-Phospho-alpha-D-ribose1-diphosphate [C5 H13 O14 P3] | 0.70 | 0.72 | 0.72 | 0.78 | 0.79 | 0.79 | 0.79 | 0.79 |
| QQFY [C28 H36 N6 O8] | 0.70 | 0.79 | 0.79 | 0.86 | 0.87 | 0.87 | 0.87 | 0.87 |
| Citicoline | 0.70 | 0.73 | 0.75 | 0.79 | 0.80 | 0.80 | 0.80 | 0.80 |
| 5-Phospho-α-D-ribose 1-diphosphate [C5 H13 O14 P3] | 0.70 | 0.72 | 0.72 | 0.78 | 0.79 | 0.79 | 0.79 | 0.79 |
| 3-Methyl-2-oxopentanoate | 0.70 | 0.68 | 0.70 | 0.71 | 0.72 | 0.72 | 0.72 | 0.72 |
| 5-phosphoribosyldiphosphate | 0.70 | 0.71 | 0.72 | 0.78 | 0.78 | 0.78 | 0.78 | 0.78 |
| 2-Hydroxyglutaric_Acid | 0.69 | 0.67 | 0.69 | 0.68 | 0.68 | 0.69 | 0.69 | 0.69 |
| gamma-Glu-Met | 0.69 | 0.66 | 0.73 | 0.73 | 0.73 | 0.73 | 0.73 | 0.73 |
| Erucamide [C22 H43 N O] | 0.69 | 0.81 | 0.80 | 0.80 | 0.80 | 0.80 | 0.80 | 0.80 |
| IMP | 0.68 | 0.69 | 0.68 | 0.67 | 0.69 | 0.69 | 0.69 | 0.69 |
| alpha-Keto-beta-methylvaleric acid | 0.67 | 0.72 | 0.70 | 0.76 | 0.78 | 0.78 | 0.78 | 0.78 |
| Glycolate | 0.67 | 0.64 | 0.74 | 0.73 | 0.74 | 0.74 | 0.74 | 0.74 |
| Acetylphosphate | 0.66 | 0.73 | 0.71 | 0.76 | 0.76 | 0.76 | 0.76 | 0.76 |
| 5-Hydroxyanthranilic acid | 0.66 | 0.65 | 0.75 | 0.74 | 0.74 | 0.74 | 0.74 | 0.74 |
| S-Methyl-L-cysteine | 0.65 | 0.63 | 0.69 | 0.69 | 0.69 | 0.69 | 0.69 | 0.69 |
| 4-Hydroxyphenylacetic acid [C8 H8 O3] | 0.64 | 0.68 | 0.68 | 0.70 | 0.70 | 0.70 | 0.70 | 0.70 |
| Indole-3-acetyl-L-aspartic acid [C14 H14 N2 O5] | 0.64 | 0.81 | 0.78 | 0.79 | 0.78 | 0.78 | 0.78 | 0.78 |
| Methylnicotinamide | 0.64 | 0.66 | 0.65 | 0.64 | 0.64 | 0.64 | 0.64 | 0.64 |
| HPO4 | 0.64 | 0.61 | 0.60 | 0.59 | 0.59 | 0.59 | 0.59 | 0.59 |
| 3Phospho-D-glycerate + 2-Phospho-D-glycerate | 0.63 | 0.61 | 0.70 | 0.70 | 0.70 | 0.70 | 0.70 | 0.70 |
| Dodecanedioic acid [C12 H22 O4] | 0.63 | 0.75 | 0.75 | 0.75 | 0.75 | 0.75 | 0.75 | 0.75 |
| Levulinic acid [C5 H8 O3] | 0.63 | 0.64 | 0.71 | 0.71 | 0.70 | 0.70 | 0.70 | 0.70 |
| Formyl-L-methionine [C6 H11 N O3 S] | 0.63 | 0.65 | 0.64 | 0.64 | 0.64 | 0.64 | 0.64 | 0.64 |
| Mevalonate-P | 0.62 | 0.59 | 0.61 | 0.62 | 0.62 | 0.62 | 0.62 | 0.62 |
| Docosahexaenoic acid [C22 H32 O2] | 0.61 | 0.88 | 0.87 | 0.89 | 0.89 | 0.89 | 0.89 | 0.89 |
| Acetoacetate | 0.61 | 0.70 | 0.71 | 0.71 | 0.71 | 0.71 | 0.71 | 0.71 |
| Indole-3-acetic acid [C10 H9 N O2] | 0.60 | 0.67 | 0.66 | 0.70 | 0.70 | 0.70 | 0.70 | 0.70 |
| Protocatechuic acid [C7 H6 O4] | 0.60 | 0.67 | 0.65 | 0.65 | 0.65 | 0.65 | 0.65 | 0.65 |
| Indoxyl sulfate | 0.60 | 0.70 | 0.71 | 0.70 | 0.70 | 0.70 | 0.70 | 0.70 |
| Phthalic acid [C8 H6 O4] | 0.59 | 0.58 | 0.57 | 0.57 | 0.58 | 0.58 | 0.58 | 0.58 |
| Epinephrine | 0.59 | 0.80 | 0.81 | 0.82 | 0.82 | 0.82 | 0.82 | 0.82 |
| Taurine | 0.58 | 0.57 | 0.69 | 0.74 | 0.74 | 0.74 | 0.74 | 0.74 |
| Asymmetric dimethylarginine | 0.58 | 0.57 | 0.71 | 0.71 | 0.71 | 0.71 | 0.71 | 0.71 |
| 3-hydroxy-Anthranilic acid | 0.58 | 0.66 | 0.64 | 0.65 | 0.64 | 0.64 | 0.64 | 0.64 |
| cGMP | 0.58 | 0.63 | 0.62 | 0.61 | 0.61 | 0.61 | 0.61 | 0.61 |
| Hexose | 0.57 | 0.55 | 0.71 | 0.70 | 0.70 | 0.70 | 0.70 | 0.70 |
| FAD | 0.57 | 0.57 | 0.59 | 0.59 | 0.59 | 0.59 | 0.59 | 0.59 |
| Threonic acid | 0.57 | 0.55 | 0.58 | 0.58 | 0.59 | 0.59 | 0.59 | 0.59 |
| 6-Hydroxynicotinic acid [C6 H5 N O3] | 0.56 | 0.55 | 0.58 | 0.58 | 0.58 | 0.58 | 0.58 | 0.58 |
| S-Adenosylhomocysteine | 0.56 | 0.55 | 0.63 | 0.62 | 0.62 | 0.63 | 0.63 | 0.63 |
| Vaniliylmanadelic acid [C9 H10 O5] | 0.56 | 0.61 | 0.60 | 0.59 | 0.59 | 0.59 | 0.60 | 0.60 |
| 2-Hydroxyphenylacetic acid [C8 H8 O3] | 0.56 | 0.53 | 0.54 | 0.56 | 0.56 | 0.56 | 0.56 | 0.56 |
| Adenosine 3',5'-cyclic monophosphate | 0.55 | 0.71 | 0.70 | 0.69 | 0.69 | 0.69 | 0.69 | 0.69 |
| N-Acetyl-L-glutamic_Acid | 0.54 | 0.84 | 0.82 | 0.83 | 0.83 | 0.83 | 0.83 | 0.83 |
| D-Glucopyranuronate [C6 H10 O7] | 0.54 | 0.66 | 0.83 | 0.99 | 1.01 | 1.01 | 1.01 | 1.01 |
| Isopentenyl pyrophosphate | 0.54 | 0.52 | 0.55 | 0.54 | 0.54 | 0.54 | 0.54 | 0.54 |
| UMP | 0.54 | 0.57 | 0.60 | 0.64 | 0.66 | 0.66 | 0.66 | 0.66 |
| gamma-Glu-Asp | 0.53 | 0.56 | 0.58 | 0.62 | 0.62 | 0.62 | 0.62 | 0.62 |
| gamma-Glu-Thr | 0.52 | 0.54 | 0.61 | 0.62 | 0.62 | 0.62 | 0.62 | 0.62 |
| Homovanillic acid | 0.52 | 0.69 | 0.68 | 0.67 | 0.67 | 0.67 | 0.68 | 0.68 |
| gamma-Glu-Taurine | 0.51 | 0.55 | 0.68 | 0.68 | 0.69 | 0.69 | 0.69 | 0.69 |
| Allantoin | 0.51 | 0.52 | 0.62 | 0.62 | 0.62 | 0.62 | 0.62 | 0.62 |
| Iso-citrate | 0.51 | 0.69 | 0.70 | 0.69 | 0.69 | 0.69 | 0.69 | 0.69 |
| Kynurenic acid | 0.51 | 0.74 | 0.74 | 0.74 | 0.74 | 0.74 | 0.74 | 0.74 |
| Diethanolamine [C4 H11 N O2] | 0.50 | 0.50 | 0.51 | 0.51 | 0.52 | 0.52 | 0.52 | 0.52 |
| Indole-3-propionic acid | 0.50 | 0.48 | 0.50 | 0.50 | 0.50 | 0.50 | 0.50 | 0.50 |
| Gly.1 | 0.50 | 0.58 | 0.67 | 0.66 | 0.66 | 0.66 | 0.66 | 0.66 |
| Arachidonic acid [C20 H32 O2] | 0.49 | 0.79 | 0.79 | 0.80 | 0.80 | 0.80 | 0.80 | 0.80 |
| 2-Dehydrogluconate [C6 H10 O7] | 0.49 | 0.63 | 0.62 | 0.62 | 0.62 | 0.62 | 0.62 | 0.62 |
| Fatty acid (C7:0) [C7 H14 O2] | 0.48 | 0.51 | 0.59 | 0.59 | 0.59 | 0.59 | 0.59 | 0.59 |
| Homocystine | 0.48 | 0.46 | 0.47 | 0.47 | 0.47 | 0.47 | 0.47 | 0.47 |
| 2-Methyl citrate [C7 H10 O7] | 0.47 | 0.45 | 0.44 | 0.44 | 0.45 | 0.45 | 0.45 | 0.45 |
| 2-Methylbenzoic acid [C8 H8 O2] | 0.46 | 0.45 | 0.52 | 0.52 | 0.55 | 0.56 | 0.56 | 0.56 |
| Dihydroxyacetone phosphate  +DL-Glyceraldehyde 3-phosphate | 0.46 | 0.45 | 0.49 | 0.57 | 0.57 | 0.57 | 0.57 | 0.57 |
| D-Glucuronate | 0.45 | 0.48 | 0.48 | 0.49 | 0.49 | 0.49 | 0.50 | 0.50 |
| 1,3-Bisphospho-D-glycerate [C3H8O10P2] | 0.45 | 0.48 | 0.56 | 0.59 | 0.59 | 0.59 | 0.59 | 0.59 |
| 4'-Phosphopantothenate | 0.45 | 0.81 | 0.80 | 0.80 | 0.80 | 0.80 | 0.80 | 0.80 |
| Pyridoxal-5'-phosphate | 0.45 | 0.43 | 0.45 | 0.45 | 0.45 | 0.45 | 0.45 | 0.45 |
| Serotonin | 0.44 | 0.45 | 0.44 | 0.55 | 0.57 | 0.57 | 0.57 | 0.57 |
| Sulfite (HSO3) | 0.44 | 0.43 | 0.43 | 0.45 | 0.45 | 0.45 | 0.45 | 0.45 |
| Orotic_acid | 0.44 | 0.46 | 0.45 | 0.45 | 0.46 | 0.46 | 0.46 | 0.46 |
| gamma-Glu-Ala | 0.43 | 0.56 | 0.65 | 0.64 | 0.64 | 0.64 | 0.64 | 0.64 |
| Docosahexaenoic acid ethyl ester [C24 H36 O2] | 0.42 | 0.66 | 0.65 | 0.66 | 0.66 | 0.66 | 0.66 | 0.66 |
| Asp-Asp [probable] | 0.41 | 0.42 | 0.54 | 0.55 | 0.55 | 0.55 | 0.55 | 0.55 |
| Gln | 0.40 | 0.56 | 0.59 | 0.60 | 0.60 | 0.60 | 0.60 | 0.60 |
| Thymine | 0.40 | 0.42 | 0.41 | 0.41 | 0.41 | 0.41 | 0.41 | 0.41 |
| Symmetric dimethylarginine | 0.40 | 0.54 | 0.57 | 0.57 | 0.57 | 0.57 | 0.57 | 0.57 |
| Cys | 0.40 | 0.41 | 0.40 | 0.42 | 0.43 | 0.43 | 0.43 | 0.43 |
| Glutathione_oxidized form | 0.38 | 0.49 | 0.49 | 0.55 | 0.55 | 0.55 | 0.55 | 0.55 |
| Terephthalic acid [C8 H6 O4] | 0.38 | 0.36 | 0.40 | 0.40 | 0.40 | 0.40 | 0.40 | 0.40 |
| Gly-Pro | 0.37 | 0.36 | 0.43 | 0.43 | 0.44 | 0.44 | 0.44 | 0.44 |
| Docosanamide [C22 H45 N O] | 0.36 | 0.42 | 0.42 | 0.49 | 0.49 | 0.49 | 0.49 | 0.49 |
| Ser | 0.36 | 0.40 | 0.52 | 0.52 | 0.52 | 0.52 | 0.52 | 0.52 |
| L-Cysteinesulfinic acid | 0.35 | 0.37 | 0.40 | 0.48 | 0.48 | 0.48 | 0.48 | 0.48 |
| 2-C-Methyl-D-erythritol 4-phosphate [C5H13O7P] | 0.35 | 0.34 | 0.35 | 0.37 | 0.37 | 0.37 | 0.37 | 0.37 |
| Lys | 0.33 | 0.51 | 0.52 | 0.52 | 0.52 | 0.53 | 0.53 | 0.53 |
| Asp-Glu-Ser [probable] | 0.32 | 0.43 | 0.53 | 0.55 | 0.55 | 0.55 | 0.55 | 0.55 |
| alpha-HMB (Hydroxy-methylbutyrate) | 0.32 | 0.44 | 0.44 | 0.44 | 0.45 | 0.45 | 0.45 | 0.45 |
| Ala-Ala | 0.32 | 0.37 | 0.49 | 0.52 | 0.51 | 0.51 | 0.52 | 0.52 |
| Pyridine | 0.31 | 0.67 | 0.65 | 0.69 | 0.70 | 0.70 | 0.70 | 0.70 |
| p-Toluenesulfonic acid [C7 H8 O3 S] | 0.31 | 0.44 | 0.51 | 0.53 | 0.53 | 0.53 | 0.53 | 0.53 |
| UDP | 0.30 | 0.35 | 0.36 | 0.36 | 0.41 | 0.41 | 0.41 | 0.41 |
| ADP | 0.30 | 0.35 | 0.50 | 0.53 | 0.54 | 0.54 | 0.54 | 0.54 |
| Glutamic acid | 0.30 | 0.78 | 0.85 | 0.84 | 0.84 | 0.84 | 0.84 | 0.84 |
| L-Ergothioneine [C9 H15 N3 O2 S] | 0.29 | 0.43 | 0.42 | 0.42 | 0.42 | 0.42 | 0.42 | 0.42 |
| Formyl-Lyr | 0.29 | 0.28 | 0.54 | 0.54 | 0.54 | 0.54 | 0.54 | 0.54 |
| Mevalonate | 0.29 | 0.31 | 0.41 | 0.42 | 0.41 | 0.42 | 0.42 | 0.42 |
| Sinapinic acid [C11 H12 O5] | 0.29 | 0.47 | 0.50 | 0.54 | 0.54 | 0.54 | 0.54 | 0.54 |
| Gluconic_acid | 0.29 | 0.27 | 0.50 | 0.50 | 0.50 | 0.50 | 0.50 | 0.50 |
| 4-Hydroxyphenylpyruvic acid | 0.28 | 0.27 | 0.31 | 0.33 | 0.33 | 0.33 | 0.33 | 0.33 |
| Cystine.1 | 0.28 | 0.34 | 0.49 | 0.49 | 0.49 | 0.49 | 0.49 | 0.49 |
| O-Phosphoserine [C3 H8 N O6 P] | 0.28 | 0.34 | 0.44 | 0.47 | 0.47 | 0.47 | 0.47 | 0.47 |
| 3-Hydroxyphenylacetic acid [C8 H8 O3] | 0.27 | 0.28 | 0.45 | 0.45 | 0.47 | 0.47 | 0.47 | 0.47 |
| Oxaloglutarate [C7 H8 O7] | 0.27 | 0.31 | 0.44 | 0.44 | 0.44 | 0.44 | 0.44 | 0.44 |
| Geranyl diphosphate | 0.27 | 0.27 | 0.46 | 0.45 | 0.45 | 0.45 | 0.45 | 0.45 |
| Glutathione_reduced form | 0.26 | 0.40 | 0.61 | 0.63 | 0.65 | 0.65 | 0.65 | 0.65 |
| D-Glucarate [C6 H10 O8] | 0.26 | 0.25 | 0.35 | 0.36 | 0.36 | 0.36 | 0.36 | 0.36 |
| Homocysteine | 0.26 | 0.25 | 0.29 | 0.29 | 0.30 | 0.30 | 0.30 | 0.30 |
| glyceraldehyde 3-phosphate/Dihidroxyacetophosphate | 0.26 | 0.30 | 0.43 | 0.43 | 0.44 | 0.44 | 0.44 | 0.44 |
| Proline | 0.26 | 0.37 | 0.51 | 0.54 | 0.55 | 0.55 | 0.55 | 0.55 |
| Arg | 0.25 | 0.28 | 0.32 | 0.32 | 0.34 | 0.34 | 0.34 | 0.34 |
| 2,3-Diphosphoglycerate | 0.25 | 0.35 | 0.42 | 0.47 | 0.47 | 0.47 | 0.47 | 0.47 |
| 3-Hydroxymethylglutaric acid [C6 H10 O5] | 0.24 | 0.58 | 0.60 | 0.59 | 0.59 | 0.59 | 0.59 | 0.59 |
| gamma-Glu-Abu | 0.23 | 0.24 | 0.29 | 0.30 | 0.31 | 0.31 | 0.31 | 0.31 |
| N-α-Acetyl-L-methionine_sulfoxide | 0.23 | 0.31 | 0.40 | 0.43 | 0.43 | 0.43 | 0.43 | 0.43 |
| Creatine | 0.23 | 0.29 | 0.28 | 0.35 | 0.34 | 0.35 | 0.35 | 0.35 |
| Oxoproline | 0.22 | 0.41 | 0.51 | 0.52 | 0.52 | 0.52 | 0.52 | 0.52 |
| Leu | 0.22 | 0.22 | 0.34 | 0.36 | 0.36 | 0.36 | 0.36 | 0.36 |
| beta-Keto-iso-valeric acid | 0.22 | 0.31 | 0.40 | 0.39 | 0.40 | 0.40 | 0.40 | 0.40 |
| AMP | 0.22 | 0.34 | 0.33 | 0.40 | 0.45 | 0.45 | 0.45 | 0.45 |
| 1-Methyluracil [C5H6N2O2] | 0.21 | 0.29 | 0.30 | 0.39 | 0.39 | 0.40 | 0.40 | 0.40 |
| Glycerol | 0.21 | 0.21 | 0.47 | 0.47 | 0.47 | 0.47 | 0.47 | 0.47 |
| Asp-Asp-Glu [probable] | 0.21 | 0.56 | 0.56 | 0.56 | 0.56 | 0.56 | 0.56 | 0.56 |
| Ethanolamine Phsophate | 0.21 | 0.21 | 0.50 | 0.52 | 0.52 | 0.52 | 0.52 | 0.52 |
| 4-Methylbenzoic acid [C8 H8 O2] | 0.20 | 0.24 | 0.27 | 0.30 | 0.30 | 0.30 | 0.31 | 0.31 |
| gamma-Glu-Lys | 0.20 | 0.23 | 0.40 | 0.40 | 0.40 | 0.40 | 0.40 | 0.40 |
| Indole | 0.19 | 0.29 | 0.28 | 0.28 | 0.28 | 0.29 | 0.29 | 0.29 |
| Dopamine | 0.19 | 0.58 | 0.65 | 0.65 | 0.65 | 0.65 | 0.65 | 0.65 |
| 5-Dehydroquinic acid [C7 H10 O6] | 0.19 | 0.37 | 0.36 | 0.37 | 0.37 | 0.37 | 0.37 | 0.37 |
| trans-Aconiticacid [C6 H6 O6] | 0.18 | 0.71 | 0.70 | 0.70 | 0.69 | 0.69 | 0.69 | 0.69 |
| Pyrimidine | 0.18 | 0.35 | 0.34 | 0.37 | 0.38 | 0.38 | 0.38 | 0.38 |
| N-Acetyl-D-glucosamine | 0.16 | 0.29 | 0.32 | 0.32 | 0.36 | 0.36 | 0.36 | 0.36 |
| N-Acetyl-D-mannosamine | 0.16 | 0.29 | 0.32 | 0.32 | 0.36 | 0.36 | 0.36 | 0.36 |
| Ile.1 | 0.15 | 0.14 | 0.30 | 0.32 | 0.32 | 0.33 | 0.33 | 0.33 |
| 5-Hydroxy-L-tryptophan | 0.15 | 0.14 | 0.14 | 0.24 | 0.25 | 0.25 | 0.26 | 0.26 |
| Glycerol_3-phosphate | 0.14 | 0.40 | 0.41 | 0.43 | 0.44 | 0.44 | 0.44 | 0.44 |
| Cysteine-glutathione Disulfide | 0.14 | 0.38 | 0.38 | 0.54 | 0.54 | 0.54 | 0.54 | 0.54 |
| 2-Deoxy-D-ribose 5-phosphate [C5 H11 O7 P] | 0.14 | 0.14 | 0.29 | 0.29 | 0.32 | 0.33 | 0.33 | 0.33 |
| cis-Aconitate | 0.14 | 0.68 | 0.68 | 0.67 | 0.67 | 0.67 | 0.67 | 0.67 |
| Met | 0.13 | 0.13 | 0.28 | 0.28 | 0.29 | 0.29 | 0.29 | 0.29 |
| N-Acetyl-Asp-Glu | 0.12 | 0.46 | 0.49 | 0.51 | 0.51 | 0.51 | 0.51 | 0.51 |
| Cytidine monophosphate | 0.12 | 0.30 | 0.43 | 0.45 | 0.45 | 0.45 | 0.45 | 0.45 |
| 2-Phosphoglycolate [C2 H5 O6 P] | 0.12 | 0.12 | 0.26 | 0.41 | 0.41 | 0.41 | 0.41 | 0.41 |
| 3,4-Dihydroxymandelic acid [C8 H8 O5] | 0.12 | 0.36 | 0.35 | 0.37 | 0.38 | 0.38 | 0.38 | 0.38 |
| Succinic anhydride [C4 H4 O3] | 0.12 | 0.11 | 0.41 | 0.44 | 0.45 | 0.45 | 0.45 | 0.45 |
| 6-phospho-gluconate | 0.11 | 0.20 | 0.41 | 0.42 | 0.42 | 0.42 | 0.42 | 0.42 |
| Anthranilate | 0.11 | 0.19 | 0.23 | 0.24 | 0.24 | 0.24 | 0.24 | 0.24 |
| Argininosuccinic acid | 0.10 | 0.57 | 0.59 | 0.59 | 0.59 | 0.59 | 0.59 | 0.59 |
| Methionine sulfoxide | 0.10 | 0.19 | 0.26 | 0.26 | 0.27 | 0.27 | 0.27 | 0.27 |
| Phosphoenolpyruvate | 0.09 | 0.09 | 0.37 | 0.40 | 0.40 | 0.40 | 0.40 | 0.40 |
| trans-2-Butenoic acid [C4 H6 O2] | 0.09 | 0.15 | 0.50 | 0.55 | 0.55 | 0.55 | 0.55 | 0.55 |
| Indole-3-butyric acid | 0.08 | 0.22 | 0.30 | 0.34 | 0.35 | 0.35 | 0.35 | 0.35 |
| gamma-Glu-Leu | 0.08 | 0.51 | 0.51 | 0.50 | 0.50 | 0.50 | 0.50 | 0.50 |
| alpha-D-Xylose1-phosphate [C5 H11 O8 P] | 0.07 | 0.07 | 0.08 | 0.15 | 0.19 | 0.19 | 0.19 | 0.19 |
| Inosine | 0.07 | 0.41 | 0.41 | 0.41 | 0.43 | 0.43 | 0.43 | 0.43 |
| Cytidine | 0.06 | 0.12 | 0.22 | 0.33 | 0.33 | 0.33 | 0.33 | 0.33 |
| Gallic acid [C7 H6 O4] | 0.05 | 0.07 | 0.10 | 0.13 | 0.17 | 0.18 | 0.18 | 0.18 |
| Lanthionine | 0.04 | 0.20 | 0.35 | 0.35 | 0.35 | 0.35 | 0.35 | 0.35 |
| 5,6-Dihydrouracil/N-Cyano-L-alanine [C4 H6 N2 O2] | 0.03 | 0.10 | 0.13 | 0.19 | 0.19 | 0.19 | 0.19 | 0.19 |
| Sebacic acid [C10 H18 O4] | 0.02 | 0.27 | 0.30 | 0.34 | 0.34 | 0.34 | 0.34 | 0.34 |
| Cytosine.1 | 0.01 | 0.36 | 0.35 | 0.37 | 0.37 | 0.37 | 0.37 | 0.37 |

|  | Fold change | p-value |
| --- | --- | --- |
| (7S_8S)-DiHODE [C18 H32 O4] | 0.437 | < 0.001 |
| gamma-Glu-Glu | 0.437 | < 0.001 |
| Urea | 0.605 | < 0.001 |
| KKK [C18 H38 N6 O4] | 3.256 | < 0.001 |
| Thymidine | 3.176 | < 0.001 |
| Xanthurenic acid | 0.532 | < 0.001 |
| Kynurenine | 0.590 | < 0.001 |
| Uric_acid | 0.766 | 0.001 |
| n-heptanoic acid [C7 H14 O2] | 3.121 | 0.001 |
| L-Norleucine [C6 H13 N O2] | 1.607 | 0.001 |
| Succinate | 0.701 | 0.001 |
| Alanine | 0.518 | 0.001 |
| 5-Hydroxyindole-3-acetic acid | 0.634 | 0.001 |
| Glucose 1-Phosphate | 0.488 | 0.001 |
| Adenine | 3.629 | 0.002 |
| cAMP | 0.505 | 0.002 |
| Trp | 0.658 | 0.002 |
| His | 0.667 | 0.002 |
| Theobromine [C7 H8 N4 O2] | 0.222 | 0.002 |
| GT [C6 H12 N2 O4] | 0.622 | 0.002 |
| Hydroxyproline | 0.670 | 0.002 |
| Aica ribonucleotide | 0.758 | 0.003 |
| Cytosine | 0.319 | 0.003 |
| Gentisic acid [C7 H6 O4] | 0.186 | 0.003 |
| Paraxanthine [C7 H8 N4 O2] | 0.313 | 0.003 |
| Phe | 0.672 | 0.003 |
| L-Norepinephrine | 2.267 | 0.003 |
| 2,3-Dihydroxybenzoate [C7 H6 O4] | 0.051 | 0.003 |
| Butylbutyrate [C8 H16 O2] | 2.517 | 0.003 |
| Hexadecanamide [C16 H33 N O] | 3.223 | 0.003 |
| Di-hexose | 0.298 | 0.004 |
| gamma-Glu-Ser | 0.757 | 0.004 |
| Val | 0.671 | 0.004 |
| 3-Hydroxy-DL-kynurenine | 0.433 | 0.005 |
| Quinaldic acid | 0.489 | 0.005 |
| Dimethylglycine | 0.714 | 0.006 |
| Creatinine | 0.736 | 0.006 |
| Glycerol 2-phosphate | 0.637 | 0.006 |
| Tyr | 0.740 | 0.007 |
| Stearamide [C18 H37 N O] | 8.214 | 0.007 |
| Cortisol | 1.745 | 0.008 |
| 4-Hydroxyphenyllactic acid [C9 H10 O4] | 0.350 | 0.008 |
| 5α-Dihydrotestosterone [C19 H30 O2] | 1.698 | 0.009 |

**Supplementary table S3. Metabolites significantly different between patients with depression and HCs at the baseline**

Abbreviations: His, histidine; KKK, Lys-Lys-Lys; Phe, phenylalanine; Trp, tryptophan; Tyr, tyrosine; Val, valine.

Amino acids and oligo-peptides are highlighted in red.

**Supplementary table S4: VIP scores for each metabolite calculated for Fig. 2B**

|  | Comp. 1 | Comp. 2 | Comp. 3 | Comp. 4 | Comp. 5 | Comp. 6 | Comp. 7 | Comp. 8 |
| --- | --- | --- | --- | --- | --- | --- | --- | --- |
| 2-Aminobutyric acid | 3.00 | 2.62 | 2.36 | 2.28 | 2.23 | 2.22 | 2.21 | 2.20 |
| L-Norleucine [C6 H13 N O2] | 2.64 | 2.30 | 2.18 | 2.11 | 2.07 | 2.06 | 2.06 | 2.05 |
| gamma-Glu-Ala | 2.57 | 2.27 | 2.04 | 1.95 | 1.93 | 1.92 | 1.92 | 1.91 |
| beta-Hydroxybutyrate | 2.48 | 2.22 | 2.03 | 1.95 | 1.91 | 1.90 | 1.89 | 1.89 |
| Ornithine | 2.48 | 2.17 | 1.99 | 1.91 | 1.87 | 1.86 | 1.86 | 1.85 |
| 2-Oxobutyrate | 2.32 | 2.11 | 1.92 | 1.86 | 1.83 | 1.82 | 1.82 | 1.81 |
| 2-Dehydrogluconate [C6 H10 O7] | 2.29 | 2.03 | 1.83 | 1.78 | 1.75 | 1.74 | 1.74 | 1.73 |
| Ile | 2.28 | 2.01 | 1.91 | 1.85 | 1.82 | 1.81 | 1.80 | 1.80 |
| Hydroxyproline | 2.22 | 1.94 | 1.77 | 1.70 | 1.74 | 1.73 | 1.72 | 1.72 |
| Ile.1 | 2.20 | 2.00 | 1.92 | 1.85 | 1.81 | 1.80 | 1.80 | 1.79 |
| beta-HMB (Hydroxy-methylbutyrate) | 2.16 | 1.92 | 1.81 | 1.76 | 1.74 | 1.73 | 1.72 | 1.72 |
| ADP-ribose 2'-phosphate [C15 H24 N5 O17 P3] | 2.12 | 2.07 | 1.87 | 1.81 | 1.78 | 1.78 | 1.78 | 1.78 |
| 9-Oxo-ODE [C18 H30 O3] | 2.07 | 1.81 | 1.74 | 1.67 | 1.63 | 1.62 | 1.62 | 1.61 |
| Leu | 2.06 | 1.84 | 1.81 | 1.74 | 1.71 | 1.70 | 1.70 | 1.69 |
| Glucose 1-Phosphate | 2.02 | 1.76 | 1.73 | 1.66 | 1.63 | 1.62 | 1.62 | 1.62 |
| 3-Methyladipic acid [C7 H12 O4] | 2.00 | 1.75 | 1.58 | 1.52 | 1.49 | 1.48 | 1.47 | 1.47 |
| Butyrate | 2.00 | 1.82 | 1.70 | 1.64 | 1.60 | 1.60 | 1.59 | 1.59 |
| glyceraldehyde 3-phosphate/Dihidroxyacetophosphate | 1.94 | 1.69 | 1.60 | 1.54 | 1.51 | 1.51 | 1.50 | 1.50 |
| Lys | 1.93 | 1.69 | 1.62 | 1.55 | 1.53 | 1.52 | 1.51 | 1.51 |
| gamma-Glu-Ser | 1.93 | 1.70 | 1.55 | 1.66 | 1.66 | 1.65 | 1.64 | 1.64 |
| keto-isocaproic acid | 1.92 | 1.84 | 1.67 | 1.60 | 1.58 | 1.58 | 1.57 | 1.57 |
| 2-Hydroxyisovaleric acid [C5 H10 O3] | 1.92 | 1.77 | 1.70 | 1.65 | 1.63 | 1.63 | 1.62 | 1.62 |
| 2-Isopropylmalic acid [C7 H12 O5] | 1.84 | 1.61 | 1.45 | 1.40 | 1.38 | 1.37 | 1.37 | 1.36 |
| 12-Oxo phytodienoic acid [C18 H28 O3] | 1.83 | 1.83 | 1.82 | 1.75 | 1.73 | 1.73 | 1.72 | 1.73 |
| Levulinic acid [C5 H8 O3] | 1.83 | 1.74 | 1.56 | 1.50 | 1.48 | 1.48 | 1.47 | 1.47 |
| Phe | 1.81 | 1.58 | 1.47 | 1.42 | 1.39 | 1.39 | 1.39 | 1.39 |
| gamma-Glu-His | 1.80 | 1.69 | 1.52 | 1.46 | 1.44 | 1.43 | 1.43 | 1.43 |
| 2-Hydroxyhippuric acid [C9 H9 N O4] | 1.80 | 1.58 | 1.42 | 1.36 | 1.37 | 1.37 | 1.37 | 1.37 |
| Dihydrouracil | 1.80 | 1.70 | 1.54 | 1.49 | 1.46 | 1.46 | 1.45 | 1.45 |
| Val | 1.75 | 1.53 | 1.45 | 1.45 | 1.42 | 1.42 | 1.42 | 1.42 |
| Phenylpyruvic acid [C9 H8 O3] | 1.70 | 1.55 | 1.53 | 1.47 | 1.44 | 1.44 | 1.43 | 1.43 |
| Succinic anhydride [C4 H4 O3] | 1.70 | 1.50 | 1.41 | 1.35 | 1.34 | 1.34 | 1.34 | 1.34 |
| Cys | 1.68 | 1.47 | 1.33 | 1.28 | 1.26 | 1.25 | 1.25 | 1.25 |
| gamma-Glu-Trp | 1.68 | 1.50 | 1.54 | 1.48 | 1.46 | 1.45 | 1.45 | 1.44 |
| 2-Methylbenzoic acid [C8 H8 O2] | 1.67 | 1.49 | 1.38 | 1.33 | 1.31 | 1.30 | 1.30 | 1.30 |
| 4-Aminobutyric acid | 1.66 | 1.55 | 1.45 | 1.44 | 1.42 | 1.41 | 1.41 | 1.41 |
| Di-hexose | 1.65 | 1.46 | 1.32 | 1.26 | 1.24 | 1.23 | 1.23 | 1.23 |
| gamma-Glu-Abu | 1.64 | 1.65 | 1.62 | 1.56 | 1.53 | 1.54 | 1.53 | 1.53 |
| 2-Keto-4-methylthiobutyric_acid | 1.62 | 1.52 | 1.42 | 1.36 | 1.34 | 1.33 | 1.33 | 1.33 |
| His | 1.61 | 1.43 | 1.36 | 1.31 | 1.28 | 1.28 | 1.28 | 1.28 |
| Acetyl carnitine | 1.59 | 1.52 | 1.45 | 1.39 | 1.38 | 1.37 | 1.37 | 1.37 |
| 3-Methyl-2-oxobutanoate | 1.58 | 1.58 | 1.44 | 1.39 | 1.36 | 1.35 | 1.35 | 1.34 |
| N-Acetyl-L-glutamic_Acid | 1.58 | 1.42 | 1.36 | 1.33 | 1.32 | 1.31 | 1.31 | 1.31 |
| 4-Methyl-2-oxopentanoate | 1.57 | 1.38 | 1.26 | 1.21 | 1.26 | 1.25 | 1.25 | 1.25 |
| 3-Hydroxyphenylacetic acid [C8 H8 O3] | 1.57 | 1.57 | 1.41 | 1.35 | 1.34 | 1.33 | 1.33 | 1.33 |
| Gluconic_acid | 1.53 | 1.38 | 1.30 | 1.26 | 1.24 | 1.23 | 1.23 | 1.22 |
| Hippuric acid | 1.51 | 1.34 | 1.25 | 1.22 | 1.25 | 1.25 | 1.24 | 1.24 |
| Tryp | 1.51 | 1.32 | 1.21 | 1.18 | 1.16 | 1.16 | 1.17 | 1.17 |
| Theobromine [C7 H8 N4 O2] | 1.50 | 1.40 | 1.30 | 1.41 | 1.39 | 1.39 | 1.38 | 1.38 |
| Guanosine | 1.50 | 1.63 | 1.48 | 1.45 | 1.45 | 1.44 | 1.44 | 1.44 |
| Methionine sulfoxide | 1.50 | 1.31 | 1.21 | 1.19 | 1.16 | 1.16 | 1.15 | 1.16 |
| 5-Keto-gluconate [C6 H10 O7] | 1.47 | 1.28 | 1.16 | 1.24 | 1.24 | 1.25 | 1.25 | 1.25 |
| Phthalic acid [C8 H6 O4] | 1.46 | 1.31 | 1.29 | 1.26 | 1.24 | 1.24 | 1.23 | 1.23 |
| N-Acetylneuraminic_acid | 1.43 | 1.29 | 1.16 | 1.20 | 1.19 | 1.19 | 1.19 | 1.19 |
| Anthranilate | 1.41 | 1.41 | 1.48 | 1.42 | 1.43 | 1.43 | 1.43 | 1.43 |
| Acetoacetate | 1.41 | 1.23 | 1.23 | 1.25 | 1.24 | 1.23 | 1.23 | 1.23 |
| Uracil | 1.40 | 1.45 | 1.31 | 1.26 | 1.23 | 1.22 | 1.22 | 1.22 |
| Maltitol/Lactitol [C12 H24 O11] | 1.39 | 1.29 | 1.16 | 1.12 | 1.12 | 1.12 | 1.11 | 1.11 |
| beta-hydroxy-iso-butyrate | 1.37 | 1.20 | 1.21 | 1.25 | 1.26 | 1.25 | 1.25 | 1.25 |
| Paraxanthine [C7 H8 N4 O2] | 1.36 | 1.36 | 1.27 | 1.41 | 1.40 | 1.39 | 1.40 | 1.40 |
| Ferulic acid [C10 H10 O4] | 1.36 | 1.31 | 1.19 | 1.15 | 1.13 | 1.13 | 1.12 | 1.12 |
| Met | 1.35 | 1.19 | 1.27 | 1.23 | 1.21 | 1.20 | 1.20 | 1.20 |
| 6-Hydroxynicotinic acid [C6 H5 N O3] | 1.35 | 1.19 | 1.09 | 1.05 | 1.03 | 1.02 | 1.02 | 1.02 |
| Glutaric acid [C5 H8 O4] | 1.35 | 1.36 | 1.24 | 1.19 | 1.16 | 1.17 | 1.17 | 1.17 |
| cyclic FMN [C17 H19 N4 O8 P] | 1.34 | 1.37 | 1.24 | 1.19 | 1.16 | 1.16 | 1.16 | 1.15 |
| Fatty acid (C6:0) [C6 H12 O2] | 1.32 | 1.23 | 1.12 | 1.26 | 1.26 | 1.25 | 1.25 | 1.25 |
| Methylnicotinamide | 1.29 | 1.19 | 1.08 | 1.15 | 1.14 | 1.13 | 1.13 | 1.13 |
| 3-Methyl-2-oxopentanoate | 1.28 | 1.35 | 1.23 | 1.18 | 1.16 | 1.15 | 1.15 | 1.14 |
| Cysteic_acid | 1.27 | 1.33 | 1.21 | 1.16 | 1.15 | 1.15 | 1.14 | 1.14 |
| beta-Ala | 1.26 | 1.17 | 1.08 | 1.09 | 1.08 | 1.08 | 1.07 | 1.07 |
| Ethyl palmitoleate [C18 H34 O2] | 1.25 | 1.09 | 1.07 | 1.07 | 1.07 | 1.07 | 1.07 | 1.07 |
| Hexose | 1.23 | 1.44 | 1.29 | 1.25 | 1.23 | 1.22 | 1.22 | 1.21 |
| KKK [C18 H38 N6 O4] | 1.22 | 1.21 | 1.11 | 1.07 | 1.04 | 1.05 | 1.05 | 1.05 |
| Dopamine | 1.22 | 1.18 | 1.30 | 1.46 | 1.43 | 1.42 | 1.42 | 1.42 |
| ADP | 1.21 | 1.23 | 1.13 | 1.10 | 1.13 | 1.13 | 1.12 | 1.12 |
| 5-Hydroxy-L-tryptophan | 1.21 | 1.13 | 1.16 | 1.26 | 1.23 | 1.23 | 1.22 | 1.22 |
| 5,6-Dihydrouracil/N-Cyano-L-alanine [C4 H6 N2 O2] | 1.21 | 1.09 | 1.03 | 0.99 | 0.97 | 0.97 | 0.96 | 0.97 |
| gamma-Glu-Taurine | 1.20 | 1.05 | 1.07 | 1.06 | 1.12 | 1.11 | 1.12 | 1.12 |
| Deoxyuridine [C9 H12 N2 O5] | 1.20 | 1.28 | 1.15 | 1.15 | 1.13 | 1.12 | 1.12 | 1.12 |
| Dehydroepiandrosterone (DHEA) [C19 H28 O2] | 1.20 | 1.11 | 1.00 | 1.04 | 1.02 | 1.02 | 1.02 | 1.01 |
| 4-Hydroxybenzoic acid [C7 H6 O3] | 1.18 | 1.20 | 1.16 | 1.15 | 1.13 | 1.12 | 1.12 | 1.11 |
| Ethanolamine Phsophate | 1.17 | 1.26 | 1.14 | 1.10 | 1.11 | 1.10 | 1.09 | 1.09 |
| Pantothenic acid | 1.17 | 1.08 | 0.97 | 0.97 | 0.96 | 0.96 | 0.96 | 0.96 |
| Glucose 6-Phosphate | 1.17 | 1.17 | 1.06 | 1.03 | 1.11 | 1.11 | 1.11 | 1.11 |
| Cytosine | 1.15 | 1.13 | 1.03 | 0.98 | 0.99 | 0.98 | 0.98 | 0.98 |
| 4-Methylbenzoic acid [C8 H8 O2] | 1.15 | 1.12 | 1.17 | 1.12 | 1.11 | 1.12 | 1.12 | 1.12 |
| Adenylsuccinic acid | 1.15 | 1.04 | 0.93 | 0.96 | 0.97 | 0.96 | 0.96 | 0.97 |
| Shikimic acid [C7 H10 O5] | 1.14 | 1.06 | 1.32 | 1.28 | 1.25 | 1.24 | 1.24 | 1.24 |
| gamma-Glu-Gly | 1.14 | 1.14 | 1.02 | 0.98 | 0.98 | 0.98 | 0.98 | 0.98 |
| Dodecanedioic acid [C12 H22 O4] | 1.13 | 0.99 | 1.05 | 1.14 | 1.16 | 1.15 | 1.15 | 1.15 |
| Glycolate | 1.13 | 1.35 | 1.21 | 1.16 | 1.15 | 1.15 | 1.15 | 1.14 |
| tetranor-12(R)-HETE [C16 H26 O3] | 1.13 | 1.18 | 1.27 | 1.22 | 1.22 | 1.21 | 1.21 | 1.21 |
| gamma-Glu-Cys | 1.12 | 1.02 | 1.09 | 1.12 | 1.11 | 1.10 | 1.10 | 1.10 |
| 2-Methyl-3-Hydroxybutyric acid [C5 H10 O3] | 1.12 | 0.98 | 0.88 | 0.85 | 0.83 | 0.85 | 0.86 | 0.86 |
| Cys-Gly | 1.11 | 0.98 | 0.93 | 1.06 | 1.04 | 1.03 | 1.04 | 1.03 |
| Quinaldic acid | 1.10 | 1.07 | 1.13 | 1.12 | 1.09 | 1.10 | 1.09 | 1.09 |
| Carnitine | 1.10 | 1.31 | 1.18 | 1.14 | 1.12 | 1.12 | 1.12 | 1.12 |
| Palmitoleic acid [C16 H30 O2] | 1.09 | 0.98 | 0.97 | 0.96 | 0.96 | 0.96 | 0.97 | 0.97 |
| 2-Succinyl cysteine | 1.09 | 1.09 | 1.02 | 1.02 | 1.00 | 1.00 | 1.01 | 1.00 |
| L-Cysteinesulfinic acid | 1.09 | 1.00 | 1.13 | 1.09 | 1.07 | 1.07 | 1.07 | 1.07 |
| Leucinic acid [C6 H12 O3] | 1.08 | 0.98 | 1.12 | 1.16 | 1.17 | 1.16 | 1.16 | 1.16 |
| 2-Aminonicotinic acid [C6 H6 N2 O2] | 1.08 | 1.17 | 1.49 | 1.44 | 1.52 | 1.52 | 1.51 | 1.51 |
| Valproic acid/2-Ethylhexanoic acid [C8 H16 O2] | 1.08 | 0.95 | 0.86 | 0.85 | 0.85 | 0.85 | 0.85 | 0.85 |
| Pyridoxal-5'-phosphate | 1.08 | 0.94 | 1.08 | 1.10 | 1.10 | 1.09 | 1.09 | 1.09 |
| 3,4-Dihydroxymandelic acid [C8 H8 O5] | 1.07 | 1.00 | 1.19 | 1.16 | 1.13 | 1.13 | 1.13 | 1.13 |
| Terephthalic acid [C8 H6 O4] | 1.07 | 1.33 | 1.34 | 1.44 | 1.42 | 1.42 | 1.42 | 1.42 |
| alpha-Keto-beta-methylvaleric acid | 1.05 | 0.96 | 0.91 | 0.87 | 0.86 | 0.87 | 0.87 | 0.87 |
| Ascorbic acid 2-sulfate [tentative] | 1.04 | 0.95 | 0.86 | 0.84 | 0.86 | 0.86 | 0.88 | 0.88 |
| gamma-Glu-Lys | 1.04 | 0.96 | 0.93 | 0.91 | 0.89 | 0.90 | 0.90 | 0.90 |
| GDP | 1.03 | 0.99 | 0.89 | 0.90 | 0.94 | 0.95 | 0.95 | 0.94 |
| N-α-Acetyl-L-methionine_sulfoxide | 1.02 | 0.92 | 0.84 | 0.87 | 0.87 | 0.88 | 0.88 | 0.88 |
| Gentisic acid [C7 H6 O4] | 1.02 | 1.31 | 1.18 | 1.21 | 1.19 | 1.19 | 1.18 | 1.18 |
| Ribose5-phosphate + Ribulose5-phosphate | 1.00 | 1.06 | 0.95 | 0.91 | 0.97 | 0.96 | 0.97 | 0.97 |
| cAMP | 1.00 | 1.19 | 1.33 | 1.27 | 1.25 | 1.25 | 1.24 | 1.24 |
| 2-Phosphoglycolate [C2 H5 O6 P] | 1.00 | 0.98 | 0.88 | 0.85 | 0.84 | 0.85 | 0.86 | 0.86 |
| 2-Methyl citrate [C7 H10 O7] | 1.00 | 1.00 | 0.94 | 0.94 | 0.97 | 0.96 | 0.96 | 0.96 |
| Pimelic acid [C7 H12 O4] | 0.99 | 0.87 | 1.06 | 1.06 | 1.05 | 1.09 | 1.09 | 1.09 |
| Kynurenic acid | 0.99 | 0.92 | 1.21 | 1.16 | 1.14 | 1.14 | 1.13 | 1.13 |
| Glycerol | 0.97 | 1.32 | 1.19 | 1.15 | 1.12 | 1.12 | 1.11 | 1.11 |
| Sulfite (HSO3) | 0.97 | 0.85 | 0.83 | 0.95 | 1.04 | 1.04 | 1.04 | 1.04 |
| Tartaric acid [C4 H6 O6] | 0.97 | 0.85 | 0.79 | 0.82 | 0.81 | 0.80 | 0.80 | 0.80 |
| Asp-Glu-Ser [probable] | 0.96 | 1.01 | 1.12 | 1.07 | 1.05 | 1.05 | 1.05 | 1.04 |
| Glutathione_reduced form | 0.96 | 1.31 | 1.50 | 1.46 | 1.49 | 1.50 | 1.49 | 1.49 |
| Pyruvate | 0.95 | 1.10 | 0.99 | 0.95 | 0.94 | 0.94 | 0.93 | 0.93 |
| trans-2-Butenoic acid [C4 H6 O2] | 0.94 | 1.01 | 0.91 | 0.87 | 0.93 | 0.93 | 0.92 | 0.92 |
| Proline | 0.94 | 0.99 | 0.90 | 0.88 | 0.86 | 0.87 | 0.87 | 0.87 |
| Methyl-Lys | 0.93 | 0.99 | 0.90 | 1.01 | 1.02 | 1.02 | 1.01 | 1.01 |
| Taurine | 0.92 | 0.89 | 0.94 | 0.90 | 1.00 | 1.00 | 1.00 | 0.99 |
| gamma-Glu-Phe | 0.92 | 1.08 | 1.04 | 1.06 | 1.04 | 1.06 | 1.05 | 1.05 |
| Formyl-Lyr | 0.92 | 1.65 | 1.51 | 1.45 | 1.43 | 1.42 | 1.42 | 1.41 |
| Creatine_phosphate | 0.92 | 0.80 | 0.74 | 0.73 | 0.73 | 0.73 | 0.73 | 0.74 |
| formylkynurenine | 0.91 | 0.83 | 1.14 | 1.09 | 1.08 | 1.08 | 1.07 | 1.07 |
| Cytidine monophosphate | 0.90 | 0.87 | 1.01 | 0.99 | 0.98 | 0.98 | 0.98 | 0.97 |
| Indole-3-acetic acid [C10 H9 N O2] | 0.90 | 0.79 | 0.71 | 0.70 | 0.70 | 0.70 | 0.70 | 0.70 |
| Gallic acid [C7 H6 O4] | 0.90 | 0.78 | 0.82 | 0.84 | 0.83 | 0.84 | 0.84 | 0.84 |
| Asp | 0.89 | 0.80 | 0.72 | 0.76 | 0.74 | 0.74 | 0.75 | 0.75 |
| Fatty acid (C7:0) [C7 H14 O2] | 0.89 | 1.10 | 1.03 | 1.00 | 0.98 | 0.98 | 0.97 | 0.97 |
| cGMP | 0.88 | 0.79 | 0.80 | 0.83 | 0.81 | 0.86 | 0.86 | 0.87 |
| Sebacic acid [C10 H18 O4] | 0.88 | 0.85 | 0.84 | 0.81 | 0.79 | 0.79 | 0.80 | 0.80 |
| Orotic_acid | 0.88 | 0.81 | 0.95 | 0.96 | 0.94 | 0.97 | 0.97 | 0.97 |
| FAD | 0.88 | 0.84 | 0.83 | 0.79 | 0.78 | 0.77 | 0.78 | 0.78 |
| Phosphoenolpyruvate | 0.88 | 0.83 | 0.76 | 0.75 | 0.74 | 0.74 | 0.75 | 0.75 |
| gamma-Glu-Glu | 0.88 | 0.77 | 0.70 | 0.71 | 0.70 | 0.70 | 0.71 | 0.71 |
| Fructose 6-Phosphate | 0.87 | 0.79 | 0.71 | 0.74 | 0.81 | 0.83 | 0.82 | 0.83 |
| Cysteine-glutathione Disulfide | 0.87 | 0.79 | 1.01 | 0.97 | 1.05 | 1.05 | 1.05 | 1.05 |
| Gly-Leu | 0.86 | 0.84 | 0.94 | 0.90 | 0.88 | 0.88 | 0.88 | 0.89 |
| Carbamoyl-DL-aspartic_acid | 0.86 | 1.02 | 0.98 | 0.99 | 1.02 | 1.03 | 1.03 | 1.03 |
| Cortisol | 0.84 | 0.79 | 0.73 | 0.70 | 0.72 | 0.72 | 0.72 | 0.72 |
| 2-Deoxy-D-ribose 5-phosphate [C5 H11 O7 P] | 0.84 | 0.78 | 0.70 | 0.71 | 0.70 | 0.70 | 0.71 | 0.72 |
| (7S_8S)-DiHODE [C18 H32 O4] | 0.83 | 0.79 | 0.72 | 0.97 | 0.95 | 0.94 | 0.95 | 0.95 |
| Cytidine | 0.82 | 0.73 | 0.70 | 0.70 | 0.69 | 0.72 | 0.72 | 0.73 |
| n-heptanoic acid [C7 H14 O2] | 0.82 | 1.14 | 1.08 | 1.11 | 1.09 | 1.09 | 1.08 | 1.09 |
| O-Acetyl-L-serine | 0.82 | 0.74 | 0.70 | 0.87 | 0.86 | 0.86 | 0.85 | 0.86 |
| Ophthalmic Acid | 0.81 | 0.88 | 0.85 | 0.82 | 0.80 | 0.81 | 0.81 | 0.81 |
| Citicoline | 0.80 | 0.87 | 0.84 | 0.81 | 0.85 | 0.87 | 0.87 | 0.87 |
| Asp-Asp [probable] | 0.80 | 0.87 | 0.87 | 0.84 | 0.83 | 0.83 | 0.83 | 0.83 |
| 1-Methyluracil [C5H6N2O2] | 0.79 | 1.01 | 0.93 | 0.93 | 0.91 | 0.92 | 0.92 | 0.92 |
| ADP-ribose | 0.79 | 0.96 | 0.87 | 0.90 | 0.92 | 0.92 | 0.92 | 0.92 |
| gamma-Glu-Met | 0.79 | 0.81 | 0.82 | 0.86 | 0.88 | 0.88 | 0.88 | 0.88 |
| Gly | 0.78 | 0.68 | 0.62 | 0.61 | 0.59 | 0.62 | 0.64 | 0.64 |
| S-Adenosylmethionine | 0.78 | 0.87 | 0.98 | 1.08 | 1.06 | 1.06 | 1.05 | 1.05 |
| Vitamine B2 | 0.78 | 0.79 | 0.71 | 0.69 | 0.67 | 0.70 | 0.71 | 0.70 |
| Dihydroorotate | 0.77 | 0.81 | 0.74 | 0.72 | 0.78 | 0.78 | 0.78 | 0.78 |
| 2-C-Methyl-D-erythritol 4-phosphate [C5H13O7P] | 0.76 | 0.69 | 0.64 | 0.61 | 0.60 | 0.61 | 0.62 | 0.65 |
| p-Toluenesulfonic acid [C7 H8 O3 S] | 0.76 | 0.72 | 0.94 | 0.90 | 0.89 | 0.89 | 0.90 | 0.90 |
| Arg | 0.76 | 0.71 | 0.97 | 0.93 | 0.91 | 0.91 | 0.91 | 0.91 |
| Succinate | 0.75 | 0.76 | 1.03 | 1.15 | 1.13 | 1.13 | 1.13 | 1.13 |
| HPO4 | 0.75 | 0.71 | 0.93 | 0.96 | 0.94 | 0.94 | 0.95 | 0.95 |
| Indoxyl sulfate | 0.75 | 0.72 | 0.79 | 0.78 | 0.79 | 0.81 | 0.80 | 0.81 |
| Urea | 0.74 | 0.85 | 0.77 | 0.74 | 0.73 | 0.73 | 0.75 | 0.75 |
| beta-Keto-iso-valeric acid | 0.74 | 0.75 | 0.67 | 0.65 | 0.63 | 0.63 | 0.64 | 0.64 |
| 3-Hydroxymethylglutaric acid [C6 H10 O5] | 0.74 | 0.87 | 0.97 | 0.93 | 0.93 | 0.92 | 0.92 | 0.92 |
| Pyroglutamic acid | 0.73 | 0.82 | 0.93 | 0.93 | 0.91 | 0.91 | 0.91 | 0.91 |
| Glutamic acid | 0.73 | 0.73 | 0.66 | 0.73 | 0.73 | 0.73 | 0.73 | 0.74 |
| Allantoin | 0.73 | 0.87 | 0.81 | 0.85 | 0.84 | 0.83 | 0.83 | 0.83 |
| 3-Methyl-L-Histidine | 0.73 | 0.77 | 0.76 | 0.90 | 0.89 | 0.88 | 0.88 | 0.88 |
| Picolinic acid | 0.73 | 1.00 | 0.92 | 0.89 | 0.89 | 0.88 | 0.88 | 0.89 |
| L-Norepinephrine | 0.73 | 0.64 | 0.58 | 0.65 | 0.64 | 0.66 | 0.66 | 0.68 |
| Indole-3-lactic acid [C11 H11 N O3] | 0.72 | 1.04 | 0.94 | 1.02 | 1.01 | 1.00 | 1.00 | 1.00 |
| 5-Hydroxyanthranilic acid | 0.72 | 0.99 | 0.95 | 0.91 | 0.90 | 0.89 | 0.89 | 0.89 |
| 5α-Dihydrotestosterone [C19 H30 O2] | 0.72 | 0.77 | 0.70 | 0.76 | 0.78 | 0.78 | 0.78 | 0.79 |
| Nicotinic_Acid | 0.72 | 0.99 | 0.92 | 0.88 | 0.88 | 0.88 | 0.88 | 0.88 |
| gamma-Glu-Leu | 0.71 | 0.62 | 0.99 | 0.96 | 0.95 | 0.95 | 0.96 | 0.96 |
| N-Acetyl-D-glucosamine | 0.71 | 0.64 | 0.87 | 0.86 | 0.84 | 0.86 | 0.86 | 0.86 |
| N-Acetyl-D-mannosamine | 0.71 | 0.64 | 0.87 | 0.86 | 0.84 | 0.86 | 0.86 | 0.86 |
| 2-Hydroxyphenylacetic acid [C8 H8 O3] | 0.71 | 0.74 | 0.68 | 0.66 | 0.67 | 0.66 | 0.66 | 0.67 |
| Benzoic acid [C7 H6 O2] | 0.71 | 0.86 | 0.79 | 0.81 | 0.81 | 0.81 | 0.81 | 0.81 |
| Caffeine | 0.71 | 0.71 | 0.65 | 0.97 | 1.00 | 1.01 | 1.02 | 1.02 |
| 2,6-Pyridinedicarboxylic acid [C7 H5 N O4] | 0.70 | 1.00 | 0.92 | 0.89 | 0.89 | 0.89 | 0.89 | 0.89 |
| Quinolinate | 0.70 | 1.00 | 0.92 | 0.89 | 0.89 | 0.89 | 0.89 | 0.89 |
| Fucose/Rhamnose [C6 H12 O5] | 0.69 | 0.79 | 0.73 | 0.71 | 0.74 | 0.74 | 0.74 | 0.74 |
| Iso-citrate | 0.69 | 0.92 | 0.94 | 0.90 | 0.88 | 0.88 | 0.88 | 0.88 |
| GT [C6 H12 N2 O4] | 0.67 | 0.81 | 0.77 | 0.82 | 0.88 | 0.88 | 0.87 | 0.87 |
| gamma-Glu-Thr | 0.66 | 1.10 | 0.99 | 0.95 | 0.93 | 0.93 | 0.93 | 0.94 |
| LKKK [C24 H49 N7 O5] | 0.66 | 0.69 | 0.79 | 0.83 | 0.83 | 0.84 | 0.83 | 0.83 |
| Mandelic acid [C8 H8 O3] | 0.65 | 0.65 | 0.85 | 0.84 | 0.82 | 0.82 | 0.83 | 0.83 |
| Uric_acid | 0.65 | 0.65 | 0.74 | 0.92 | 0.93 | 0.93 | 0.93 | 0.94 |
| 2-Hydroxyglutaric_Acid | 0.65 | 1.02 | 1.08 | 1.05 | 1.05 | 1.05 | 1.05 | 1.05 |
| Acetylphosphate | 0.65 | 0.79 | 0.77 | 0.90 | 0.91 | 0.91 | 0.91 | 0.91 |
| Dmethyl-Lys | 0.64 | 0.98 | 0.90 | 0.87 | 0.85 | 0.85 | 0.85 | 0.84 |
| 2,3-Dihydroxybenzoate [C7 H6 O4] | 0.64 | 0.59 | 0.56 | 0.89 | 0.96 | 0.96 | 0.95 | 0.95 |
| Adenosine | 0.64 | 0.56 | 0.50 | 0.49 | 0.67 | 0.67 | 0.67 | 0.67 |
| 3-hydroxy-Anthranilic acid | 0.63 | 0.58 | 0.77 | 0.84 | 0.84 | 0.84 | 0.85 | 0.85 |
| alpha-HMB (Hydroxy-methylbutyrate) | 0.63 | 0.63 | 0.90 | 0.90 | 0.91 | 0.91 | 0.91 | 0.93 |
| gamma-Glu-Arg | 0.63 | 1.05 | 1.01 | 1.02 | 1.03 | 1.03 | 1.02 | 1.03 |
| Carnosine | 0.62 | 0.57 | 0.75 | 0.86 | 0.85 | 0.85 | 0.86 | 0.86 |
| Glycerol_3-phosphate | 0.62 | 0.69 | 0.80 | 0.81 | 0.83 | 0.85 | 0.85 | 0.85 |
| 4'-Phosphopantothenate | 0.62 | 0.72 | 0.83 | 0.80 | 0.80 | 0.80 | 0.80 | 0.80 |
| Hexanoylcarnitine [C13 H25 N O4] | 0.61 | 0.72 | 0.90 | 0.90 | 0.93 | 0.97 | 0.96 | 0.96 |
| Cystathionine | 0.60 | 0.53 | 0.89 | 0.87 | 0.85 | 0.85 | 0.85 | 0.85 |
| Thymine | 0.59 | 0.90 | 1.56 | 1.71 | 1.68 | 1.69 | 1.68 | 1.68 |
| Arachidonic acid [C20 H32 O2] | 0.59 | 0.55 | 0.65 | 0.70 | 0.68 | 0.68 | 0.70 | 0.70 |
| Tyr | 0.58 | 0.51 | 0.49 | 0.47 | 0.46 | 0.47 | 0.53 | 0.53 |
| 5-Hydroxyindole-3-acetic acid | 0.58 | 0.87 | 0.88 | 0.86 | 0.85 | 0.84 | 0.85 | 0.85 |
| GTKK [C18 H36 N6 O6] | 0.57 | 0.75 | 0.72 | 0.74 | 0.73 | 0.73 | 0.73 | 0.73 |
| Asp-Asp-Glu [probable] | 0.57 | 0.53 | 0.88 | 0.85 | 0.92 | 0.93 | 0.93 | 0.93 |
| Glutathione_oxidized form | 0.56 | 0.66 | 0.59 | 0.57 | 0.61 | 0.63 | 0.64 | 0.64 |
| Acetyl-glycine [C4 H7 N O3] | 0.56 | 0.99 | 0.90 | 1.00 | 0.98 | 0.97 | 0.98 | 0.97 |
| Gln | 0.56 | 0.64 | 0.89 | 0.93 | 0.92 | 0.92 | 0.91 | 0.91 |
| gamma-Glu-Val | 0.56 | 0.79 | 0.82 | 0.78 | 0.78 | 0.77 | 0.78 | 0.78 |
| Lanthionine | 0.56 | 0.64 | 0.67 | 0.73 | 0.73 | 0.73 | 0.73 | 0.74 |
| Cystine.1 | 0.56 | 0.77 | 0.74 | 0.71 | 0.69 | 0.70 | 0.70 | 0.70 |
| Homocysteic acid | 0.56 | 0.67 | 0.67 | 0.68 | 0.70 | 0.71 | 0.72 | 0.72 |
| Norepinephrine | 0.55 | 0.93 | 0.89 | 0.99 | 0.98 | 0.97 | 0.98 | 0.97 |
| Creatinine | 0.54 | 0.63 | 1.06 | 1.03 | 1.03 | 1.03 | 1.03 | 1.03 |
| Argininosuccinic acid | 0.54 | 0.81 | 0.83 | 0.81 | 0.80 | 0.80 | 0.81 | 0.80 |
| 4-Hydroxyphenylacetic acid [C8 H8 O3] | 0.53 | 0.48 | 1.27 | 1.22 | 1.20 | 1.21 | 1.21 | 1.21 |
| Inosine | 0.53 | 0.71 | 0.64 | 0.63 | 0.73 | 0.73 | 0.73 | 0.72 |
| 3-Indoleacetic acid | 0.52 | 0.54 | 0.61 | 0.62 | 0.61 | 0.61 | 0.61 | 0.61 |
| Diethanolamine [C4 H11 N O2] | 0.51 | 0.77 | 0.94 | 0.91 | 0.90 | 0.91 | 0.91 | 0.91 |
| 5-Dehydroquinic acid [C7 H10 O6] | 0.51 | 1.01 | 1.00 | 0.97 | 0.97 | 0.97 | 0.98 | 0.98 |
| Butylbutyrate [C8 H16 O2] | 0.50 | 0.44 | 0.40 | 0.51 | 0.53 | 0.53 | 0.54 | 0.54 |
| Geranyl diphosphate | 0.50 | 1.04 | 0.95 | 0.92 | 0.90 | 0.89 | 0.90 | 0.89 |
| Isopentenyl pyrophosphate | 0.49 | 0.72 | 0.65 | 0.65 | 0.66 | 0.66 | 0.67 | 0.67 |
| Adenine | 0.49 | 0.83 | 0.81 | 0.82 | 0.81 | 0.81 | 0.81 | 0.81 |
| Ala-Ala | 0.49 | 0.79 | 0.82 | 0.79 | 0.77 | 0.79 | 0.78 | 0.78 |
| Orthophosphate | 0.49 | 0.51 | 0.91 | 0.92 | 0.90 | 0.90 | 0.90 | 0.90 |
| alpha-Keto-iso-valeric acid | 0.49 | 0.77 | 0.74 | 0.78 | 0.77 | 0.79 | 0.80 | 0.80 |
| ATP | 0.49 | 0.44 | 0.42 | 0.47 | 0.61 | 0.64 | 0.64 | 0.64 |
| N6-Me-Adenosine [C11 H15 N5 O4] | 0.48 | 0.79 | 0.74 | 0.74 | 0.72 | 0.72 | 0.75 | 0.74 |
| Azelaic acid [C9 H16 O4] | 0.48 | 0.52 | 0.79 | 0.77 | 0.81 | 0.80 | 0.80 | 0.80 |
| IMP | 0.46 | 0.41 | 0.41 | 0.41 | 0.50 | 0.51 | 0.51 | 0.51 |
| Thiosulfate | 0.46 | 0.51 | 0.80 | 0.77 | 0.75 | 0.75 | 0.77 | 0.77 |
| Nicotinamide_[Negative] | 0.46 | 0.42 | 0.53 | 0.53 | 0.66 | 0.66 | 0.67 | 0.66 |
| gamma-Glu-Tyr | 0.45 | 0.64 | 0.58 | 0.79 | 0.82 | 0.82 | 0.84 | 0.84 |
| 6-phospho-gluconate | 0.45 | 0.69 | 0.75 | 0.72 | 0.70 | 0.70 | 0.71 | 0.71 |
| Cytosine.1 | 0.44 | 0.41 | 0.62 | 0.60 | 0.59 | 0.58 | 0.58 | 0.58 |
| Oxoproline | 0.44 | 0.70 | 0.86 | 0.89 | 0.88 | 0.88 | 0.87 | 0.87 |
| Lactate | 0.44 | 0.66 | 0.63 | 0.69 | 0.68 | 0.68 | 0.68 | 0.69 |
| gamma-Glu-Gln | 0.43 | 0.65 | 0.79 | 0.77 | 0.77 | 0.77 | 0.77 | 0.77 |
| N3,N4-Dimethyl-L-arginine [C8 H18 N4 O2] | 0.43 | 0.81 | 0.73 | 0.71 | 0.77 | 0.77 | 0.79 | 0.79 |
| Oxaloglutarate [C7 H8 O7] | 0.42 | 0.67 | 0.67 | 0.64 | 0.68 | 0.68 | 0.68 | 0.68 |
| Erythose [C4 H8 O4] | 0.41 | 0.52 | 0.62 | 0.59 | 0.65 | 0.65 | 0.67 | 0.67 |
| Protocatechuic acid [C7 H6 O4] | 0.41 | 0.41 | 0.52 | 0.56 | 0.55 | 0.55 | 0.55 | 0.56 |
| Serotonin | 0.41 | 0.36 | 0.44 | 0.50 | 0.67 | 0.68 | 0.68 | 0.68 |
| Eicosapentaenoic acid [C20 H30 O2] | 0.41 | 0.42 | 0.39 | 0.49 | 0.50 | 0.50 | 0.51 | 0.51 |
| Vaniliylmanadelic acid [C9 H10 O5] | 0.41 | 0.36 | 0.62 | 0.72 | 0.73 | 0.75 | 0.75 | 0.75 |
| UDP | 0.40 | 0.38 | 0.41 | 0.47 | 0.63 | 0.65 | 0.65 | 0.65 |
| N-Acetylaspartate | 0.40 | 0.61 | 0.61 | 0.68 | 0.70 | 0.70 | 0.71 | 0.70 |
| Indole-3-propionic acid | 0.39 | 0.41 | 0.89 | 1.02 | 1.01 | 1.03 | 1.03 | 1.03 |
| Creatine | 0.38 | 0.36 | 0.69 | 0.66 | 0.65 | 0.71 | 0.71 | 0.70 |
| 2-oxoglutarate | 0.38 | 0.54 | 0.71 | 0.68 | 0.69 | 0.69 | 0.69 | 0.69 |
| Homovanillic acid | 0.38 | 0.33 | 0.91 | 0.87 | 0.88 | 0.89 | 0.89 | 0.89 |
| D-Glyceric_acid | 0.38 | 0.44 | 0.69 | 1.05 | 1.04 | 1.04 | 1.03 | 1.03 |
| UMP | 0.37 | 0.32 | 0.33 | 0.41 | 0.55 | 0.58 | 0.58 | 0.58 |
| Alanine | 0.37 | 0.34 | 0.82 | 0.79 | 0.78 | 0.78 | 0.79 | 0.79 |
| Pyrimidine | 0.36 | 0.32 | 0.33 | 0.58 | 0.58 | 0.59 | 0.59 | 0.59 |
| Mevalonate-P | 0.35 | 0.59 | 0.59 | 0.57 | 0.58 | 0.59 | 0.59 | 0.59 |
| UTP | 0.35 | 0.31 | 0.37 | 0.43 | 0.61 | 0.63 | 0.63 | 0.63 |
| 4-Hydroxyphenyllactic acid [C9 H10 O4] | 0.35 | 0.41 | 0.44 | 0.42 | 0.42 | 0.42 | 0.43 | 0.43 |
| Malate | 0.34 | 0.66 | 0.66 | 0.63 | 0.62 | 0.63 | 0.63 | 0.63 |
| Glycerol 2-phosphate | 0.34 | 0.31 | 0.48 | 0.47 | 0.54 | 0.53 | 0.55 | 0.55 |
| Choline.1 | 0.33 | 0.29 | 0.34 | 0.63 | 0.67 | 0.66 | 0.66 | 0.68 |
| Itaconic acid | 0.32 | 0.47 | 0.73 | 0.80 | 0.80 | 0.81 | 0.81 | 0.81 |
| trans-Aconiticacid [C6 H6 O6] | 0.32 | 0.93 | 0.84 | 0.89 | 0.88 | 0.87 | 0.88 | 0.88 |
| Gly-Pro | 0.32 | 0.71 | 0.65 | 0.66 | 0.65 | 0.65 | 0.64 | 0.64 |
| H4P3O10 | 0.31 | 0.28 | 0.44 | 0.54 | 0.66 | 0.68 | 0.68 | 0.67 |
| alpha-keto-butyrate | 0.31 | 0.36 | 0.72 | 0.70 | 0.70 | 0.70 | 0.70 | 0.70 |
| Octylphosphonic acid [C8 H19 O3 P] | 0.31 | 0.34 | 0.51 | 0.49 | 0.48 | 0.49 | 0.49 | 0.52 |
| Docosahexaenoic acid ethyl ester [C24 H36 O2] | 0.31 | 0.63 | 0.68 | 0.69 | 0.68 | 0.68 | 0.69 | 0.69 |
| Citrulline | 0.31 | 0.35 | 0.60 | 0.57 | 0.60 | 0.61 | 0.63 | 0.62 |
| Kynurenine | 0.31 | 0.62 | 0.77 | 0.74 | 0.72 | 0.72 | 0.73 | 0.74 |
| Docosapentaenoic acid [C22 H34 O2] | 0.31 | 0.29 | 0.42 | 0.41 | 0.42 | 0.45 | 0.46 | 0.46 |
| alpha-D-Xylose1-phosphate [C5 H11 O8 P] | 0.30 | 0.28 | 0.29 | 0.28 | 0.46 | 0.46 | 0.47 | 0.47 |
| GMP | 0.30 | 0.52 | 0.53 | 0.55 | 0.71 | 0.72 | 0.72 | 0.72 |
| Homocystine | 0.29 | 0.38 | 0.78 | 0.84 | 0.82 | 0.82 | 0.83 | 0.83 |
| O-Phosphoserine [C3 H8 N O6 P] | 0.28 | 0.88 | 0.81 | 0.77 | 0.76 | 0.76 | 0.75 | 0.75 |
| Decanamide [C10 H21 N O] | 0.28 | 0.36 | 0.44 | 0.42 | 0.41 | 0.43 | 0.43 | 0.44 |
| Asymmetric dimethylarginine | 0.27 | 0.57 | 0.73 | 0.70 | 0.70 | 0.70 | 0.71 | 0.71 |
| CTP | 0.27 | 0.27 | 0.36 | 0.43 | 0.62 | 0.64 | 0.64 | 0.64 |
| 1,3-Bisphospho-D-glycerate [C3H8O10P2] | 0.27 | 0.43 | 0.75 | 0.72 | 0.71 | 0.70 | 0.71 | 0.71 |
| Hexadecanamide [C16 H33 N O] | 0.26 | 0.42 | 0.38 | 0.37 | 0.39 | 0.39 | 0.43 | 0.44 |
| Sedoheptulose_7-phosphate | 0.25 | 0.32 | 0.39 | 0.42 | 0.61 | 0.61 | 0.62 | 0.61 |
| Docosahexaenoic acid [C22 H32 O2] | 0.25 | 0.40 | 0.56 | 0.57 | 0.56 | 0.58 | 0.58 | 0.58 |
| N-Acetyl-Asp-Glu | 0.25 | 0.42 | 0.89 | 0.86 | 0.85 | 0.85 | 0.84 | 0.84 |
| Asn | 0.25 | 0.64 | 0.83 | 0.82 | 0.81 | 0.81 | 0.81 | 0.81 |
| Gly-Gly | 0.25 | 0.64 | 0.83 | 0.82 | 0.81 | 0.81 | 0.81 | 0.81 |
| 2-Deoxy-glucose_6-phosphate | 0.25 | 0.29 | 0.49 | 0.47 | 0.46 | 0.48 | 0.48 | 0.48 |
| AMP | 0.25 | 0.44 | 0.47 | 0.51 | 0.66 | 0.67 | 0.67 | 0.67 |
| D-Glucuronate | 0.24 | 0.39 | 0.35 | 0.37 | 0.37 | 0.37 | 0.38 | 0.38 |
| Aica ribonucleotide | 0.23 | 0.74 | 0.70 | 0.88 | 0.89 | 0.89 | 0.89 | 0.90 |
| Homocysteine | 0.23 | 0.45 | 0.49 | 0.55 | 0.57 | 0.56 | 0.56 | 0.57 |
| Oxomalonate [C3 H2 O5] | 0.23 | 0.66 | 0.65 | 0.62 | 0.63 | 0.65 | 0.65 | 0.65 |
| gamma-Glu-Asp | 0.22 | 0.30 | 0.67 | 0.65 | 0.64 | 0.65 | 0.65 | 0.65 |
| L-Carnitine | 0.22 | 0.60 | 0.54 | 0.73 | 0.75 | 0.74 | 0.75 | 0.76 |
| Mevalonate | 0.22 | 0.83 | 0.86 | 0.97 | 0.95 | 0.94 | 0.94 | 0.96 |
| S-Adenosylhomocysteine | 0.21 | 0.40 | 0.67 | 0.73 | 0.73 | 0.73 | 0.73 | 0.73 |
| Citrate | 0.20 | 0.56 | 0.68 | 0.67 | 0.65 | 0.65 | 0.66 | 0.67 |
| Symmetric dimethylarginine | 0.20 | 0.82 | 0.79 | 0.76 | 0.84 | 0.83 | 0.83 | 0.83 |
| 2,3-Diphosphoglycerate | 0.20 | 0.21 | 0.79 | 0.77 | 0.75 | 0.75 | 0.76 | 0.75 |
| 3Phospho-D-glycerate + 2-Phospho-D-glycerate | 0.19 | 0.23 | 0.60 | 0.65 | 0.68 | 0.68 | 0.69 | 0.69 |
| Sinapinic acid [C11 H12 O5] | 0.19 | 0.63 | 0.75 | 0.77 | 0.78 | 0.79 | 0.79 | 0.79 |
| Fumarate | 0.19 | 0.56 | 0.63 | 0.62 | 0.62 | 0.63 | 0.63 | 0.64 |
| Indole-3-butyric acid | 0.19 | 0.16 | 0.65 | 0.62 | 0.66 | 0.67 | 0.68 | 0.69 |
| Epinephrine | 0.19 | 0.16 | 0.31 | 0.42 | 0.61 | 0.65 | 0.65 | 0.66 |
| Urocanic acid | 0.18 | 0.26 | 0.72 | 0.84 | 0.85 | 0.84 | 0.84 | 0.84 |
| Dihydroxyacetone phosphate  +DL-Glyceraldehyde 3-phosphate | 0.17 | 0.17 | 0.35 | 0.45 | 0.49 | 0.52 | 0.53 | 0.53 |
| GTP | 0.17 | 0.15 | 0.27 | 0.37 | 0.56 | 0.58 | 0.58 | 0.58 |
| cis-Aconitate | 0.17 | 0.97 | 0.87 | 0.90 | 0.89 | 0.88 | 0.89 | 0.89 |
| QQFY [C28 H36 N6 O8] | 0.16 | 0.26 | 0.25 | 0.78 | 0.77 | 0.77 | 0.77 | 0.77 |
| Ser | 0.15 | 0.65 | 0.73 | 0.71 | 0.75 | 0.74 | 0.74 | 0.74 |
| Adenosine 3',5'-cyclic monophosphate | 0.14 | 0.19 | 0.86 | 0.86 | 0.84 | 0.84 | 0.83 | 0.84 |
| L-Cysteine_S-sulfate | 0.14 | 0.32 | 0.72 | 0.73 | 0.74 | 0.74 | 0.75 | 0.75 |
| Homoserine | 0.14 | 0.65 | 0.70 | 0.69 | 0.73 | 0.73 | 0.73 | 0.73 |
| Thr | 0.14 | 0.65 | 0.70 | 0.69 | 0.73 | 0.73 | 0.73 | 0.73 |
| L-Ergothioneine [C9 H15 N3 O2 S] | 0.14 | 0.45 | 0.66 | 1.01 | 0.99 | 0.99 | 0.99 | 0.99 |
| Choline | 0.13 | 0.64 | 0.58 | 0.58 | 0.57 | 0.58 | 0.58 | 0.58 |
| Citraconic acid | 0.13 | 0.59 | 0.73 | 0.70 | 0.70 | 0.70 | 0.71 | 0.71 |
| Furoic acid | 0.13 | 0.53 | 0.69 | 0.67 | 0.65 | 0.65 | 0.66 | 0.67 |
| Indole-3-acetyl-L-aspartic acid [C14 H14 N2 O5] | 0.12 | 0.42 | 0.70 | 0.68 | 0.67 | 0.67 | 0.67 | 0.67 |
| Uridine | 0.11 | 0.23 | 0.26 | 0.56 | 0.56 | 0.62 | 0.62 | 0.62 |
| 4-Hydroxyphenylpyruvic acid | 0.10 | 0.89 | 1.01 | 1.07 | 1.05 | 1.05 | 1.05 | 1.05 |
| S-Methyl-L-cysteine | 0.10 | 0.62 | 0.56 | 0.54 | 0.53 | 0.53 | 0.54 | 0.54 |
| Erucamide [C22 H43 N O] | 0.10 | 0.10 | 0.10 | 0.15 | 0.22 | 0.22 | 0.26 | 0.26 |
| Norophthalmic acid | 0.10 | 0.45 | 0.68 | 0.67 | 0.68 | 0.69 | 0.69 | 0.69 |
| Pyridoxine | 0.10 | 0.17 | 0.59 | 0.58 | 0.58 | 0.58 | 0.59 | 0.59 |
| Cystine | 0.09 | 0.17 | 0.20 | 0.36 | 0.35 | 0.35 | 0.36 | 0.36 |
| Docosanamide [C22 H45 N O] | 0.09 | 0.37 | 0.34 | 0.37 | 0.38 | 0.38 | 0.41 | 0.42 |
| D-Glucarate [C6 H10 O8] | 0.07 | 0.57 | 0.62 | 0.60 | 0.60 | 0.60 | 0.60 | 0.60 |
| Thymidine | 0.07 | 0.10 | 0.70 | 0.72 | 0.72 | 0.72 | 0.72 | 0.72 |
| Dimethylglycine | 0.07 | 0.52 | 0.49 | 0.51 | 0.50 | 0.51 | 0.51 | 0.51 |
| Pyridine | 0.07 | 0.55 | 0.62 | 0.60 | 0.59 | 0.59 | 0.59 | 0.59 |
| Riboflavin-5'-phosphate | 0.06 | 0.07 | 0.68 | 0.72 | 0.71 | 0.72 | 0.72 | 0.72 |
| Hypotaurine | 0.06 | 0.78 | 0.71 | 0.79 | 0.82 | 0.82 | 0.82 | 0.81 |
| Sarcosine | 0.06 | 0.66 | 0.65 | 0.73 | 0.72 | 0.72 | 0.71 | 0.72 |
| Gly.1 | 0.05 | 0.63 | 0.75 | 0.73 | 0.73 | 0.73 | 0.72 | 0.72 |
| Formyl-L-methionine [C6 H11 N O3 S] | 0.04 | 0.57 | 0.58 | 0.58 | 0.57 | 0.62 | 0.62 | 0.62 |
| Indole | 0.04 | 0.10 | 0.82 | 0.79 | 0.79 | 0.80 | 0.79 | 0.79 |
| Pyrophosphate | 0.04 | 0.26 | 0.60 | 0.82 | 0.88 | 0.89 | 0.89 | 0.89 |
| 3-Hydroxy-DL-kynurenine | 0.04 | 0.68 | 0.94 | 0.93 | 0.95 | 0.95 | 0.95 | 0.94 |
| Threonic acid | 0.03 | 0.11 | 0.62 | 0.72 | 0.72 | 0.71 | 0.71 | 0.71 |
| Stearamide [C18 H37 N O] | 0.03 | 0.41 | 0.37 | 0.40 | 0.44 | 0.44 | 0.45 | 0.45 |
| Hypoxanthine | 0.02 | 0.59 | 0.58 | 0.56 | 0.69 | 0.69 | 0.69 | 0.69 |
| 5-Phospho-alpha-D-ribose1-diphosphate [C5 H13 O14 P3] | 0.02 | 0.20 | 0.33 | 0.43 | 0.57 | 0.60 | 0.60 | 0.60 |
| D-Glucopyranuronate [C6 H10 O7] | 0.02 | 0.07 | 0.15 | 0.59 | 0.58 | 0.57 | 0.57 | 0.57 |
| 5-phosphoribosyldiphosphate | 0.02 | 0.21 | 0.33 | 0.43 | 0.57 | 0.60 | 0.60 | 0.60 |
| Xanthurenic acid | 0.01 | 0.73 | 0.65 | 0.63 | 0.62 | 0.62 | 0.62 | 0.63 |
| 5-Phospho-α-D-ribose 1-diphosphate [C5 H13 O14 P3] | 0.01 | 0.21 | 0.35 | 0.44 | 0.58 | 0.60 | 0.61 | 0.61 |

**Supplementary table S5. The list of metabolites showed significant positive correlations with MADRS score**

|  | Pearson coefficient | p-value |
| --- | --- | --- |
| Leu | 0.462 | 0.001 |
| Ile | 0.429 | 0.002 |
| Carnosine | 0.407 | 0.004 |
| gamma-Glu-Phe | 0.399 | 0.004 |
| Pyridoxal-5'-phosphate | 0.395 | 0.005 |
| 2-Aminobutyric acid | 0.378 | 0.007 |
| 2-Oxobutyrate | 0.371 | 0.009 |
| 4-Aminobutyric acid | 0.356 | 0.012 |
| Met | 0.349 | 0.014 |
| Vaniliylmanadelic acid [C9 H10 O5] | 0.339 | 0.017 |
| gamma-Glu-Abu | 0.332 | 0.020 |
| Kynurenic acid | 0.330 | 0.020 |
| gamma-Glu-His | 0.314 | 0.028 |
| S-Adenosylmethionine | 0.314 | 0.028 |
| Quinolinate | 0.309 | 0.031 |
| 2,6-Pyridinedicarboxylic acid [C7 H5 N O4] | 0.309 | 0.031 |
| Maltitol/Lactitol [C12 H24 O11] | 0.308 | 0.031 |
| Picolinic acid | 0.307 | 0.032 |
| Asp-Asp-Glu [probable] | 0.307 | 0.032 |
| Nicotinic_Acid | 0.306 | 0.032 |
| Vitamine B2 | 0.301 | 0.036 |
| Asymmetric dimethylarginine | 0.300 | 0.036 |
| Methyl-Lys | 0.299 | 0.036 |
| S-Adenosylhomocysteine | 0.294 | 0.040 |
| Acetyl carnitine | 0.294 | 0.040 |
| Acetoacetate | 0.282 | 0.049 |

Abbreviations: Ile, isoleucine; Leu, leucine; Met, methionine.

Amino acids and oligo-peptides are highlighted in red.

**Supplementary table S6. The list of metabolites showed significant negative correlations with MADRS score**

Abbreviations: Asn, asparagine; Gln, glutamine; Gly, glycine; Ser, serine; Thr, threonine.

Amino acids and oligo-peptides are highlighted in red.

|  | Pearson coefficient | p-value |
| --- | --- | --- |
| Hydroxyproline | -0.588 | 0.000 |
| Gln | -0.487 | 0.000 |
| beta-Ala | -0.465 | 0.001 |
| gamma-Glu-Ser | -0.455 | 0.001 |
| GT [C6 H12 N2 O4] | -0.452 | 0.001 |
| Oxoproline | -0.434 | 0.002 |
| Ophthalmic Acid | -0.399 | 0.004 |
| Ser | -0.397 | 0.005 |
| Homoserine | -0.380 | 0.007 |
| Thr | -0.380 | 0.007 |
| Paraxanthine [C7 H8 N4 O2] | -0.377 | 0.007 |
| Gly | -0.376 | 0.008 |
| Theobromine [C7 H8 N4 O2] | -0.351 | 0.013 |
| Asn | -0.338 | 0.017 |
| Gly-Gly | -0.338 | 0.017 |
| Norophthalmic acid | -0.334 | 0.019 |
| gamma-Glu-Ala | -0.330 | 0.020 |
| Creatine_phosphate | -0.320 | 0.025 |
| 12-Oxo phytodienoic acid [C18 H28 O3] | -0.310 | 0.030 |
| Cystine | -0.300 | 0.036 |
| gamma-Glu-Gln | -0.291 | 0.042 |
| (7S_8S)-DiHODE [C18 H32 O4] | -0.290 | 0.043 |
| Acetyl-glycine [C4 H7 N O3] | -0.282 | 0.050 |

**Supplementary table S7. VIP scores for each metabolite calculated for Fig. 3B**

|  | Comp. 1 | Comp. 2 | Comp. 3 | Comp. 4 | Comp. 5 | Comp. 6 | Comp. 7 | Comp. 8 |
| --- | --- | --- | --- | --- | --- | --- | --- | --- |
| HPO4 | 3.02 | 2.61 | 2.45 | 2.39 | 2.36 | 2.34 | 2.34 | 2.34 |
| Orthophosphate | 2.93 | 2.54 | 2.39 | 2.33 | 2.30 | 2.28 | 2.28 | 2.28 |
| Formyl-Lyr | 2.57 | 2.21 | 2.07 | 2.03 | 2.00 | 1.98 | 1.98 | 1.98 |
| GTKK [C18 H36 N6 O6] | 2.55 | 2.33 | 2.18 | 2.14 | 2.11 | 2.10 | 2.10 | 2.10 |
| ADP-ribose 2'-phosphate [C15 H24 N5 O17 P3] | 2.47 | 2.10 | 2.03 | 2.00 | 1.98 | 1.97 | 1.97 | 1.97 |
| Docosahexaenoic acid [C22 H32 O2] | 2.40 | 2.04 | 1.98 | 1.94 | 1.91 | 1.90 | 1.90 | 1.90 |
| Indole-3-acetyl-L-aspartic acid [C14 H14 N2 O5] | 2.37 | 2.21 | 2.08 | 2.03 | 2.04 | 2.03 | 2.02 | 2.02 |
| gamma-Glu-Tyr | 2.30 | 1.95 | 1.86 | 1.82 | 1.79 | 1.78 | 1.78 | 1.78 |
| cGMP | 2.28 | 2.10 | 1.97 | 1.94 | 1.91 | 1.90 | 1.90 | 1.90 |
| Eicosapentaenoic acid [C20 H30 O2] | 2.24 | 1.91 | 1.82 | 1.78 | 1.77 | 1.76 | 1.76 | 1.76 |
| Arachidonic acid [C20 H32 O2] | 2.21 | 1.90 | 1.89 | 1.87 | 1.85 | 1.83 | 1.83 | 1.83 |
| Mandelic acid [C8 H8 O3] | 2.18 | 1.86 | 1.82 | 1.79 | 1.77 | 1.77 | 1.76 | 1.76 |
| Docosapentaenoic acid [C22 H34 O2] | 2.16 | 1.84 | 1.81 | 1.79 | 1.77 | 1.76 | 1.75 | 1.75 |
| beta-Hydroxybutyrate | 2.16 | 1.84 | 1.74 | 1.70 | 1.71 | 1.70 | 1.70 | 1.70 |
| Creatine_phosphate | 2.13 | 2.02 | 1.92 | 1.88 | 1.86 | 1.85 | 1.84 | 1.84 |
| Indole | 2.09 | 1.85 | 1.76 | 1.73 | 1.71 | 1.71 | 1.71 | 1.71 |
| Oxoproline | 2.05 | 1.78 | 1.77 | 1.73 | 1.71 | 1.70 | 1.70 | 1.70 |
| Ile.1 | 2.03 | 1.72 | 1.74 | 1.70 | 1.68 | 1.67 | 1.67 | 1.67 |
| 3-Indoleacetic acid | 2.01 | 1.71 | 1.72 | 1.70 | 1.67 | 1.67 | 1.66 | 1.66 |
| 5-Hydroxyindole-3-acetic acid | 1.93 | 1.72 | 1.61 | 1.58 | 1.56 | 1.55 | 1.55 | 1.55 |
| 5α-Dihydrotestosterone [C19 H30 O2] | 1.93 | 1.64 | 1.55 | 1.52 | 1.56 | 1.56 | 1.55 | 1.55 |
| gamma-Glu-Trp | 1.90 | 1.85 | 1.73 | 1.70 | 1.68 | 1.67 | 1.67 | 1.67 |
| 4-Aminobutyric acid | 1.89 | 1.61 | 1.56 | 1.55 | 1.52 | 1.52 | 1.52 | 1.52 |
| Glucose 1-Phosphate | 1.89 | 1.66 | 1.56 | 1.55 | 1.55 | 1.55 | 1.55 | 1.55 |
| Succinate | 1.88 | 1.81 | 1.92 | 1.88 | 1.85 | 1.84 | 1.84 | 1.84 |
| Docosahexaenoic acid ethyl ester [C24 H36 O2] | 1.88 | 1.59 | 1.60 | 1.59 | 1.59 | 1.59 | 1.58 | 1.58 |
| Gln | 1.86 | 1.61 | 1.63 | 1.59 | 1.57 | 1.56 | 1.56 | 1.56 |
| 3-hydroxy-Anthranilic acid | 1.84 | 1.73 | 1.74 | 1.70 | 1.69 | 1.68 | 1.68 | 1.68 |
| Cytosine | 1.84 | 1.60 | 1.58 | 1.55 | 1.54 | 1.54 | 1.54 | 1.54 |
| Butylbutyrate [C8 H16 O2] | 1.83 | 1.64 | 1.54 | 1.51 | 1.48 | 1.48 | 1.47 | 1.48 |
| Leu | 1.75 | 1.48 | 1.52 | 1.49 | 1.47 | 1.46 | 1.46 | 1.46 |
| Cys-Gly | 1.71 | 1.50 | 1.46 | 1.44 | 1.41 | 1.41 | 1.40 | 1.41 |
| trans-2-Butenoic acid [C4 H6 O2] | 1.71 | 1.50 | 1.42 | 1.39 | 1.37 | 1.36 | 1.36 | 1.36 |
| Riboflavin-5'-phosphate | 1.70 | 1.46 | 1.48 | 1.46 | 1.44 | 1.43 | 1.44 | 1.44 |
| Cysteic_acid | 1.69 | 1.57 | 1.48 | 1.45 | 1.44 | 1.43 | 1.43 | 1.43 |
| Kynurenine | 1.69 | 1.54 | 1.44 | 1.42 | 1.40 | 1.39 | 1.39 | 1.39 |
| cAMP | 1.69 | 1.51 | 1.42 | 1.39 | 1.37 | 1.37 | 1.37 | 1.37 |
| 2-Hydroxyglutaric_Acid | 1.68 | 1.46 | 1.44 | 1.47 | 1.44 | 1.44 | 1.44 | 1.44 |
| Di-hexose | 1.67 | 1.59 | 1.52 | 1.49 | 1.47 | 1.46 | 1.46 | 1.46 |
| 5-Dehydroquinic acid [C7 H10 O6] | 1.63 | 1.50 | 1.50 | 1.47 | 1.44 | 1.44 | 1.43 | 1.43 |
| Cytidine | 1.62 | 1.38 | 1.44 | 1.41 | 1.39 | 1.38 | 1.38 | 1.38 |
| Palmitoleic acid [C16 H30 O2] | 1.60 | 1.39 | 1.35 | 1.36 | 1.34 | 1.34 | 1.34 | 1.34 |
| Shikimic acid [C7 H10 O5] | 1.60 | 1.50 | 1.41 | 1.38 | 1.36 | 1.35 | 1.35 | 1.35 |
| Homocystine | 1.60 | 1.36 | 1.41 | 1.38 | 1.39 | 1.38 | 1.38 | 1.38 |
| Asymmetric dimethylarginine | 1.59 | 1.43 | 1.35 | 1.35 | 1.33 | 1.33 | 1.33 | 1.32 |
| 3-Hydroxy-DL-kynurenine | 1.57 | 1.33 | 1.26 | 1.25 | 1.24 | 1.24 | 1.24 | 1.24 |
| Cortisol | 1.56 | 1.36 | 1.27 | 1.25 | 1.27 | 1.27 | 1.27 | 1.27 |
| QQFY [C28 H36 N6 O8] | 1.55 | 1.34 | 1.40 | 1.40 | 1.38 | 1.37 | 1.37 | 1.37 |
| Erucamide [C22 H43 N O] | 1.54 | 1.31 | 1.40 | 1.41 | 1.39 | 1.38 | 1.38 | 1.38 |
| Docosanamide [C22 H45 N O] | 1.53 | 1.34 | 1.36 | 1.37 | 1.35 | 1.34 | 1.34 | 1.34 |
| N-α-Acetyl-L-methionine_sulfoxide | 1.51 | 1.30 | 1.29 | 1.27 | 1.25 | 1.25 | 1.25 | 1.25 |
| O-Phosphoserine [C3 H8 N O6 P] | 1.50 | 1.35 | 1.27 | 1.27 | 1.29 | 1.28 | 1.28 | 1.28 |
| Isopentenyl pyrophosphate | 1.47 | 1.35 | 1.27 | 1.28 | 1.29 | 1.28 | 1.28 | 1.28 |
| Indoxyl sulfate | 1.46 | 1.45 | 1.43 | 1.39 | 1.38 | 1.37 | 1.37 | 1.37 |
| gamma-Glu-Met | 1.45 | 1.32 | 1.24 | 1.21 | 1.20 | 1.19 | 1.19 | 1.19 |
| GT [C6 H12 N2 O4] | 1.45 | 1.24 | 1.29 | 1.27 | 1.25 | 1.25 | 1.25 | 1.25 |
| Decanamide [C10 H21 N O] | 1.44 | 1.43 | 1.41 | 1.39 | 1.37 | 1.37 | 1.37 | 1.37 |
| Protocatechuic acid [C7 H6 O4] | 1.41 | 1.50 | 1.40 | 1.37 | 1.35 | 1.34 | 1.34 | 1.34 |
| KKK [C18 H38 N6 O4] | 1.41 | 1.25 | 1.23 | 1.23 | 1.23 | 1.23 | 1.22 | 1.23 |
| Met | 1.38 | 1.20 | 1.12 | 1.13 | 1.11 | 1.11 | 1.11 | 1.11 |
| p-Toluenesulfonic acid [C7 H8 O3 S] | 1.37 | 1.56 | 1.49 | 1.46 | 1.44 | 1.43 | 1.43 | 1.43 |
| Indole-3-acetic acid [C10 H9 N O2] | 1.37 | 1.19 | 1.32 | 1.29 | 1.28 | 1.27 | 1.27 | 1.27 |
| Glycerol 2-phosphate | 1.37 | 1.17 | 1.17 | 1.16 | 1.16 | 1.19 | 1.19 | 1.19 |
| LKKK [C24 H49 N7 O5] | 1.37 | 1.18 | 1.13 | 1.11 | 1.13 | 1.13 | 1.13 | 1.13 |
| Azelaic acid [C9 H16 O4] | 1.34 | 1.26 | 1.18 | 1.17 | 1.15 | 1.15 | 1.15 | 1.15 |
| 3-Hydroxymethylglutaric acid [C6 H10 O5] | 1.33 | 1.30 | 1.23 | 1.20 | 1.19 | 1.20 | 1.19 | 1.19 |
| 2-Succinyl cysteine | 1.31 | 1.34 | 1.26 | 1.24 | 1.23 | 1.23 | 1.22 | 1.22 |
| 2-Oxobutyrate | 1.30 | 1.16 | 1.10 | 1.08 | 1.09 | 1.10 | 1.10 | 1.10 |
| Sebacic acid [C10 H18 O4] | 1.29 | 1.11 | 1.27 | 1.24 | 1.23 | 1.23 | 1.23 | 1.23 |
| formylkynurenine | 1.28 | 1.16 | 1.13 | 1.11 | 1.11 | 1.11 | 1.11 | 1.11 |
| Gallic acid [C7 H6 O4] | 1.28 | 1.14 | 1.07 | 1.08 | 1.06 | 1.06 | 1.05 | 1.05 |
| Hypoxanthine | 1.27 | 1.21 | 1.14 | 1.13 | 1.13 | 1.13 | 1.13 | 1.13 |
| 2-Deoxy-D-ribose 5-phosphate [C5 H11 O7 P] | 1.26 | 1.09 | 1.05 | 1.03 | 1.05 | 1.07 | 1.07 | 1.07 |
| Kynurenic acid | 1.25 | 1.18 | 1.11 | 1.08 | 1.06 | 1.07 | 1.07 | 1.07 |
| Sulfite (HSO3) | 1.25 | 1.06 | 1.03 | 1.05 | 1.06 | 1.06 | 1.05 | 1.05 |
| (7S_8S)-DiHODE [C18 H32 O4] | 1.24 | 1.07 | 1.00 | 0.99 | 1.00 | 0.99 | 1.00 | 1.00 |
| 2-Methyl citrate [C7 H10 O7] | 1.24 | 1.07 | 1.03 | 1.01 | 1.00 | 1.00 | 1.00 | 1.00 |
| L-Cysteine_S-sulfate | 1.24 | 1.06 | 1.05 | 1.03 | 1.02 | 1.03 | 1.03 | 1.03 |
| S-Adenosylhomocysteine | 1.22 | 1.24 | 1.17 | 1.16 | 1.14 | 1.14 | 1.14 | 1.14 |
| L-Norleucine [C6 H13 N O2] | 1.21 | 1.03 | 1.10 | 1.07 | 1.12 | 1.12 | 1.12 | 1.12 |
| Ascorbic acid 2-sulfate [tentative] | 1.20 | 1.13 | 1.09 | 1.07 | 1.05 | 1.06 | 1.06 | 1.06 |
| Threonic acid | 1.18 | 1.13 | 1.22 | 1.19 | 1.19 | 1.18 | 1.18 | 1.18 |
| L-Carnitine | 1.18 | 1.10 | 1.05 | 1.03 | 1.05 | 1.05 | 1.04 | 1.04 |
| gamma-Glu-His | 1.18 | 1.00 | 1.01 | 1.03 | 1.03 | 1.04 | 1.04 | 1.04 |
| Phosphoenolpyruvate | 1.16 | 1.14 | 1.07 | 1.08 | 1.06 | 1.07 | 1.07 | 1.07 |
| gamma-Glu-Abu | 1.15 | 0.98 | 0.95 | 0.99 | 0.98 | 0.97 | 0.97 | 0.97 |
| Geranyl diphosphate | 1.14 | 1.16 | 1.12 | 1.13 | 1.14 | 1.14 | 1.14 | 1.14 |
| Paraxanthine [C7 H8 N4 O2] | 1.14 | 0.98 | 0.92 | 0.94 | 0.93 | 0.93 | 0.93 | 0.93 |
| Erythose [C4 H8 O4] | 1.14 | 1.01 | 1.00 | 0.99 | 0.98 | 0.98 | 0.97 | 0.97 |
| Maltitol/Lactitol [C12 H24 O11] | 1.13 | 0.97 | 0.99 | 0.96 | 0.99 | 0.99 | 0.99 | 0.99 |
| Dihydrouracil | 1.12 | 1.21 | 1.14 | 1.14 | 1.14 | 1.13 | 1.13 | 1.13 |
| Nicotinamide_[Negative] | 1.12 | 0.95 | 0.98 | 0.95 | 0.94 | 0.95 | 0.95 | 0.95 |
| Anthranilate | 1.10 | 0.94 | 0.91 | 0.89 | 0.88 | 0.91 | 0.91 | 0.91 |
| Asn | 1.10 | 1.24 | 1.18 | 1.15 | 1.14 | 1.13 | 1.13 | 1.13 |
| Gly-Gly | 1.10 | 1.24 | 1.18 | 1.15 | 1.14 | 1.13 | 1.13 | 1.13 |
| gamma-Glu-Val | 1.08 | 1.00 | 0.95 | 0.93 | 0.96 | 0.96 | 0.95 | 0.95 |
| beta-HMB (Hydroxy-methylbutyrate) | 1.08 | 1.28 | 1.27 | 1.25 | 1.23 | 1.24 | 1.24 | 1.23 |
| Choline.1 | 1.07 | 0.92 | 1.06 | 1.07 | 1.06 | 1.05 | 1.05 | 1.05 |
| Pyridine | 1.07 | 1.16 | 1.09 | 1.08 | 1.08 | 1.07 | 1.07 | 1.07 |
| GDP | 1.05 | 0.91 | 0.87 | 0.92 | 0.93 | 0.92 | 0.92 | 0.92 |
| Methylnicotinamide | 1.04 | 0.91 | 0.90 | 0.88 | 0.86 | 0.86 | 0.87 | 0.87 |
| Ile | 1.03 | 0.90 | 1.01 | 1.01 | 1.02 | 1.04 | 1.04 | 1.04 |
| 4-Hydroxybenzoic acid [C7 H6 O3] | 1.03 | 1.19 | 1.14 | 1.13 | 1.12 | 1.12 | 1.12 | 1.12 |
| Dimethylglycine | 1.02 | 1.13 | 1.06 | 1.10 | 1.09 | 1.09 | 1.09 | 1.09 |
| alpha-keto-butyrate | 1.01 | 1.40 | 1.32 | 1.29 | 1.28 | 1.27 | 1.27 | 1.27 |
| Gly-Leu | 1.01 | 1.06 | 1.01 | 1.04 | 1.08 | 1.08 | 1.08 | 1.08 |
| Theobromine [C7 H8 N4 O2] | 1.00 | 0.87 | 0.82 | 0.81 | 0.80 | 0.81 | 0.82 | 0.82 |
| Ethyl palmitoleate [C18 H34 O2] | 0.99 | 0.85 | 0.96 | 1.01 | 1.00 | 1.01 | 1.01 | 1.01 |
| L-Cysteinesulfinic acid | 0.98 | 1.14 | 1.07 | 1.10 | 1.10 | 1.09 | 1.09 | 1.09 |
| S-Adenosylmethionine | 0.98 | 1.01 | 0.98 | 0.97 | 0.96 | 0.97 | 0.97 | 0.97 |
| gamma-Glu-Ser | 0.96 | 0.94 | 0.88 | 0.89 | 0.91 | 0.92 | 0.92 | 0.92 |
| 5-Hydroxy-L-tryptophan | 0.96 | 0.85 | 0.83 | 0.92 | 0.91 | 0.91 | 0.91 | 0.91 |
| Acetyl carnitine | 0.95 | 1.05 | 1.03 | 1.01 | 1.04 | 1.04 | 1.04 | 1.04 |
| Choline | 0.95 | 1.06 | 0.99 | 1.03 | 1.02 | 1.02 | 1.02 | 1.02 |
| Fucose/Rhamnose [C6 H12 O5] | 0.94 | 0.88 | 0.83 | 0.81 | 0.86 | 0.86 | 0.86 | 0.86 |
| D-Glucarate [C6 H10 O8] | 0.93 | 0.96 | 1.00 | 0.99 | 0.97 | 0.97 | 0.97 | 0.97 |
| 5-Keto-gluconate [C6 H10 O7] | 0.93 | 0.94 | 0.94 | 0.92 | 0.91 | 0.92 | 0.92 | 0.92 |
| Dehydroepiandrosterone (DHEA) [C19 H28 O2] | 0.92 | 0.84 | 0.80 | 0.80 | 0.79 | 0.79 | 0.79 | 0.79 |
| Argininosuccinic acid | 0.92 | 1.13 | 1.06 | 1.06 | 1.04 | 1.04 | 1.04 | 1.04 |
| Urea | 0.92 | 0.82 | 1.09 | 1.07 | 1.05 | 1.06 | 1.06 | 1.06 |
| Urocanic acid | 0.92 | 1.02 | 1.04 | 1.02 | 1.05 | 1.04 | 1.04 | 1.04 |
| Pantothenic acid | 0.92 | 0.82 | 0.91 | 0.89 | 0.88 | 0.88 | 0.88 | 0.88 |
| Orotic_acid | 0.92 | 0.95 | 0.96 | 0.94 | 0.93 | 0.93 | 0.94 | 0.94 |
| Indole-3-lactic acid [C11 H11 N O3] | 0.91 | 0.78 | 0.83 | 0.85 | 0.86 | 0.86 | 0.86 | 0.86 |
| Hippuric acid | 0.90 | 0.77 | 0.75 | 0.74 | 0.76 | 0.75 | 0.75 | 0.76 |
| Glycolate | 0.89 | 1.16 | 1.08 | 1.07 | 1.05 | 1.05 | 1.04 | 1.04 |
| Dihydroorotate | 0.89 | 0.76 | 0.71 | 0.72 | 0.72 | 0.73 | 0.73 | 0.73 |
| Arg | 0.89 | 0.76 | 0.71 | 0.80 | 0.80 | 0.80 | 0.80 | 0.80 |
| Glucose 6-Phosphate | 0.88 | 0.75 | 0.79 | 0.82 | 0.85 | 0.84 | 0.84 | 0.85 |
| 3-Methyl-2-oxopentanoate | 0.86 | 1.03 | 1.05 | 1.03 | 1.02 | 1.01 | 1.01 | 1.01 |
| Asp | 0.86 | 0.74 | 0.72 | 0.76 | 0.79 | 0.81 | 0.81 | 0.81 |
| Glutamic acid | 0.86 | 0.73 | 0.70 | 0.79 | 0.78 | 0.78 | 0.78 | 0.78 |
| gamma-Glu-Phe | 0.85 | 1.06 | 1.00 | 0.98 | 0.97 | 0.98 | 0.98 | 0.98 |
| Gentisic acid [C7 H6 O4] | 0.85 | 0.92 | 0.88 | 0.86 | 0.85 | 0.87 | 0.87 | 0.87 |
| Valproic acid/2-Ethylhexanoic acid [C8 H16 O2] | 0.85 | 0.93 | 1.01 | 0.99 | 0.99 | 0.99 | 0.99 | 0.99 |
| Adenosine 3',5'-cyclic monophosphate | 0.84 | 0.84 | 1.02 | 1.02 | 1.01 | 1.00 | 1.00 | 1.00 |
| Cysteine-glutathione Disulfide | 0.84 | 0.85 | 1.00 | 1.01 | 0.99 | 0.99 | 0.99 | 0.99 |
| beta-Ala | 0.83 | 0.72 | 0.69 | 0.69 | 0.69 | 0.71 | 0.71 | 0.71 |
| Mevalonate | 0.81 | 0.88 | 0.85 | 0.83 | 0.82 | 0.81 | 0.81 | 0.81 |
| Oxaloglutarate [C7 H8 O7] | 0.81 | 1.22 | 1.14 | 1.12 | 1.11 | 1.11 | 1.11 | 1.11 |
| cis-Aconitate | 0.80 | 1.05 | 0.99 | 0.97 | 0.97 | 0.97 | 0.97 | 0.97 |
| Cystine.1 | 0.80 | 0.92 | 0.92 | 0.90 | 0.92 | 0.91 | 0.91 | 0.91 |
| n-heptanoic acid [C7 H14 O2] | 0.79 | 1.03 | 0.99 | 0.99 | 0.98 | 1.00 | 1.00 | 1.00 |
| trans-Aconiticacid [C6 H6 O6] | 0.79 | 1.01 | 0.95 | 0.94 | 0.94 | 0.94 | 0.94 | 0.94 |
| Carbamoyl-DL-aspartic_acid | 0.79 | 0.82 | 0.87 | 0.85 | 0.84 | 0.83 | 0.83 | 0.83 |
| gamma-Glu-Lys | 0.79 | 0.86 | 0.95 | 0.93 | 0.92 | 0.92 | 0.92 | 0.92 |
| Sarcosine | 0.79 | 0.92 | 0.93 | 0.91 | 0.92 | 0.92 | 0.92 | 0.92 |
| N-Acetyl-Asp-Glu | 0.78 | 1.10 | 1.03 | 1.01 | 0.99 | 0.99 | 0.99 | 0.99 |
| Vitamine B2 | 0.78 | 0.70 | 0.88 | 0.91 | 0.90 | 0.90 | 0.90 | 0.90 |
| Symmetric dimethylarginine | 0.78 | 1.07 | 1.01 | 0.99 | 0.98 | 0.98 | 0.98 | 0.98 |
| N-Acetyl-L-glutamic_Acid | 0.78 | 0.73 | 0.68 | 0.68 | 0.72 | 0.75 | 0.75 | 0.75 |
| Acetyl-glycine [C4 H7 N O3] | 0.78 | 1.13 | 1.06 | 1.04 | 1.03 | 1.03 | 1.03 | 1.03 |
| Ala-Ala | 0.77 | 0.95 | 0.98 | 0.95 | 0.94 | 0.94 | 0.94 | 0.94 |
| Hypotaurine | 0.77 | 0.83 | 0.77 | 0.84 | 0.84 | 0.83 | 0.83 | 0.83 |
| Adenylsuccinic acid | 0.77 | 0.93 | 1.05 | 1.02 | 1.01 | 1.00 | 1.00 | 1.00 |
| Quinaldic acid | 0.74 | 0.81 | 0.89 | 0.88 | 0.87 | 0.87 | 0.87 | 0.87 |
| Cys | 0.74 | 0.63 | 0.59 | 0.58 | 0.62 | 0.62 | 0.63 | 0.63 |
| alpha-Keto-iso-valeric acid | 0.73 | 1.11 | 1.04 | 1.05 | 1.03 | 1.03 | 1.03 | 1.03 |
| S-Methyl-L-cysteine | 0.72 | 0.72 | 0.69 | 0.69 | 0.71 | 0.71 | 0.71 | 0.71 |
| Cystine | 0.72 | 0.62 | 0.63 | 0.69 | 0.72 | 0.72 | 0.73 | 0.73 |
| keto-isocaproic acid | 0.72 | 1.07 | 1.07 | 1.04 | 1.03 | 1.03 | 1.02 | 1.02 |
| Gly.1 | 0.72 | 0.97 | 1.00 | 0.98 | 0.97 | 0.97 | 0.97 | 0.97 |
| Guanosine | 0.71 | 0.70 | 0.69 | 0.81 | 0.80 | 0.79 | 0.79 | 0.79 |
| gamma-Glu-Thr | 0.71 | 0.91 | 0.90 | 0.88 | 0.87 | 0.87 | 0.87 | 0.87 |
| Ophthalmic Acid | 0.71 | 0.76 | 0.96 | 0.94 | 0.93 | 0.92 | 0.92 | 0.92 |
| 2-oxoglutarate | 0.70 | 1.30 | 1.24 | 1.21 | 1.20 | 1.19 | 1.19 | 1.19 |
| 3-Methyl-L-Histidine | 0.70 | 0.65 | 0.62 | 0.63 | 0.65 | 0.66 | 0.66 | 0.66 |
| Formyl-L-methionine [C6 H11 N O3 S] | 0.69 | 1.19 | 1.37 | 1.33 | 1.33 | 1.32 | 1.32 | 1.32 |
| 2-Phosphoglycolate [C2 H5 O6 P] | 0.69 | 0.68 | 0.71 | 0.77 | 0.76 | 0.77 | 0.77 | 0.77 |
| 2,3-Diphosphoglycerate | 0.68 | 0.81 | 0.82 | 0.80 | 0.79 | 0.79 | 0.79 | 0.79 |
| Malate | 0.68 | 1.14 | 1.07 | 1.09 | 1.09 | 1.09 | 1.09 | 1.09 |
| D-Glyceric_acid | 0.68 | 0.81 | 0.91 | 0.92 | 0.92 | 0.92 | 0.92 | 0.92 |
| Levulinic acid [C5 H8 O3] | 0.67 | 1.02 | 0.96 | 0.94 | 0.93 | 0.93 | 0.93 | 0.93 |
| N-Acetylaspartate | 0.66 | 0.95 | 0.98 | 0.96 | 0.95 | 0.96 | 0.96 | 0.96 |
| Adenine | 0.66 | 0.76 | 0.74 | 0.72 | 0.73 | 0.72 | 0.72 | 0.72 |
| 2-Hydroxyhippuric acid [C9 H9 N O4] | 0.66 | 0.56 | 0.54 | 0.54 | 0.53 | 0.53 | 0.53 | 0.53 |
| D-Glucuronate | 0.66 | 0.68 | 0.64 | 0.63 | 0.65 | 0.70 | 0.70 | 0.70 |
| N-Acetyl-D-glucosamine | 0.65 | 0.57 | 0.77 | 0.76 | 0.76 | 0.76 | 0.76 | 0.76 |
| N-Acetyl-D-mannosamine | 0.65 | 0.57 | 0.77 | 0.76 | 0.76 | 0.76 | 0.76 | 0.76 |
| 4'-Phosphopantothenate | 0.65 | 1.05 | 1.00 | 0.98 | 0.99 | 0.99 | 0.99 | 0.99 |
| Uracil | 0.64 | 1.11 | 1.04 | 1.03 | 1.05 | 1.05 | 1.05 | 1.05 |
| Iso-citrate | 0.64 | 1.10 | 1.03 | 1.01 | 1.00 | 1.00 | 1.00 | 1.00 |
| Picolinic acid | 0.64 | 0.95 | 0.96 | 0.94 | 0.94 | 0.94 | 0.94 | 0.94 |
| 9-Oxo-ODE [C18 H30 O3] | 0.64 | 0.69 | 0.67 | 0.68 | 0.79 | 0.78 | 0.79 | 0.79 |
| Methyl-Lys | 0.64 | 0.85 | 0.82 | 0.81 | 0.80 | 0.80 | 0.80 | 0.80 |
| IMP | 0.64 | 0.61 | 0.61 | 0.63 | 0.64 | 0.64 | 0.64 | 0.64 |
| Nicotinic_Acid | 0.63 | 0.95 | 0.96 | 0.94 | 0.94 | 0.94 | 0.94 | 0.94 |
| 2,6-Pyridinedicarboxylic acid [C7 H5 N O4] | 0.62 | 0.97 | 0.97 | 0.96 | 0.95 | 0.95 | 0.95 | 0.95 |
| Quinolinate | 0.62 | 0.97 | 0.97 | 0.96 | 0.95 | 0.95 | 0.95 | 0.95 |
| Fumarate | 0.62 | 1.10 | 1.03 | 1.05 | 1.04 | 1.04 | 1.04 | 1.04 |
| 3-Methyl-2-oxobutanoate | 0.62 | 1.11 | 1.04 | 1.02 | 1.01 | 1.00 | 1.00 | 1.00 |
| Proline | 0.62 | 0.54 | 0.55 | 0.69 | 0.69 | 0.69 | 0.69 | 0.69 |
| gamma-Glu-Gln | 0.61 | 0.91 | 0.91 | 0.89 | 0.89 | 0.89 | 0.88 | 0.88 |
| Vaniliylmanadelic acid [C9 H10 O5] | 0.61 | 0.91 | 1.09 | 1.08 | 1.06 | 1.07 | 1.08 | 1.08 |
| gamma-Glu-Gly | 0.60 | 0.81 | 0.79 | 0.84 | 0.83 | 0.82 | 0.82 | 0.82 |
| Carnitine | 0.60 | 0.81 | 0.76 | 0.82 | 0.81 | 0.81 | 0.81 | 0.81 |
| gamma-Glu-Taurine | 0.59 | 0.70 | 0.79 | 0.81 | 0.80 | 0.81 | 0.81 | 0.81 |
| Epinephrine | 0.59 | 0.54 | 0.59 | 0.59 | 0.59 | 0.62 | 0.62 | 0.62 |
| Dmethyl-Lys | 0.59 | 0.67 | 0.63 | 0.64 | 0.63 | 0.63 | 0.63 | 0.63 |
| gamma-Glu-Arg | 0.59 | 0.89 | 0.91 | 0.90 | 0.89 | 0.90 | 0.90 | 0.90 |
| Xanthurenic acid | 0.58 | 0.56 | 0.74 | 0.75 | 0.73 | 0.75 | 0.75 | 0.75 |
| Norophthalmic acid | 0.58 | 0.79 | 0.81 | 0.79 | 0.80 | 0.80 | 0.80 | 0.80 |
| Deoxyuridine [C9 H12 N2 O5] | 0.58 | 0.97 | 0.91 | 0.89 | 0.90 | 0.89 | 0.89 | 0.89 |
| gamma-Glu-Ala | 0.58 | 0.70 | 0.72 | 0.78 | 0.77 | 0.78 | 0.78 | 0.78 |
| Benzoic acid [C7 H6 O2] | 0.57 | 0.77 | 0.73 | 0.75 | 0.76 | 0.76 | 0.76 | 0.76 |
| Methionine sulfoxide | 0.56 | 0.47 | 0.49 | 0.65 | 0.65 | 0.68 | 0.68 | 0.68 |
| UDP | 0.56 | 0.48 | 0.55 | 0.65 | 0.67 | 0.67 | 0.67 | 0.67 |
| AMP | 0.56 | 0.52 | 0.60 | 0.67 | 0.72 | 0.71 | 0.71 | 0.71 |
| Cytidine monophosphate | 0.55 | 1.04 | 1.00 | 1.01 | 1.01 | 1.00 | 1.00 | 1.00 |
| Oxomalonate [C3 H2 O5] | 0.55 | 0.89 | 0.88 | 0.88 | 0.87 | 0.86 | 0.86 | 0.86 |
| ADP-ribose | 0.55 | 0.70 | 0.67 | 0.82 | 0.81 | 0.80 | 0.80 | 0.80 |
| Aica ribonucleotide | 0.55 | 0.57 | 0.54 | 0.56 | 0.55 | 0.59 | 0.59 | 0.59 |
| Glutathione_reduced form | 0.54 | 0.51 | 0.52 | 0.51 | 0.51 | 0.51 | 0.52 | 0.52 |
| 2-Dehydrogluconate [C6 H10 O7] | 0.53 | 0.83 | 0.90 | 0.88 | 0.90 | 0.90 | 0.90 | 0.90 |
| Sedoheptulose_7-phosphate | 0.53 | 0.45 | 0.49 | 0.54 | 0.65 | 0.65 | 0.65 | 0.65 |
| 6-phospho-gluconate | 0.52 | 0.76 | 0.79 | 0.77 | 0.77 | 0.77 | 0.77 | 0.77 |
| Ornithine | 0.52 | 0.48 | 0.52 | 0.67 | 0.67 | 0.68 | 0.68 | 0.68 |
| Itaconic acid | 0.52 | 0.47 | 0.56 | 0.57 | 0.56 | 0.56 | 0.56 | 0.56 |
| Pyridoxine | 0.52 | 0.50 | 0.67 | 0.66 | 0.68 | 0.68 | 0.68 | 0.68 |
| Homocysteine | 0.51 | 0.45 | 0.50 | 0.54 | 0.55 | 0.55 | 0.55 | 0.55 |
| Glutaric acid [C5 H8 O4] | 0.51 | 0.76 | 0.79 | 0.82 | 0.82 | 0.81 | 0.81 | 0.81 |
| Ethanolamine Phsophate | 0.50 | 0.91 | 0.86 | 0.84 | 0.87 | 0.88 | 0.88 | 0.88 |
| gamma-Glu-Glu | 0.50 | 0.65 | 0.61 | 0.59 | 0.59 | 0.60 | 0.60 | 0.61 |
| 1,3-Bisphospho-D-glycerate [C3H8O10P2] | 0.50 | 0.76 | 0.81 | 0.79 | 0.78 | 0.78 | 0.78 | 0.78 |
| 6-Hydroxynicotinic acid [C6 H5 N O3] | 0.50 | 0.55 | 0.62 | 0.65 | 0.67 | 0.67 | 0.67 | 0.67 |
| Phthalic acid [C8 H6 O4] | 0.49 | 0.51 | 0.50 | 0.59 | 0.61 | 0.61 | 0.62 | 0.62 |
| 2-Methylbenzoic acid [C8 H8 O2] | 0.49 | 0.70 | 0.75 | 0.76 | 0.79 | 0.79 | 0.79 | 0.79 |
| 2-Deoxy-glucose_6-phosphate | 0.48 | 0.53 | 0.62 | 0.61 | 0.61 | 0.60 | 0.60 | 0.60 |
| L-Norepinephrine | 0.48 | 0.82 | 0.80 | 0.79 | 0.78 | 0.77 | 0.77 | 0.77 |
| Gly | 0.48 | 0.41 | 0.46 | 0.60 | 0.62 | 0.62 | 0.62 | 0.62 |
| Asp-Asp [probable] | 0.48 | 0.82 | 0.79 | 0.78 | 0.77 | 0.78 | 0.78 | 0.78 |
| 3-Hydroxyphenylacetic acid [C8 H8 O3] | 0.48 | 0.88 | 0.90 | 0.92 | 0.91 | 0.91 | 0.91 | 0.91 |
| Gluconic_acid | 0.48 | 0.85 | 0.83 | 0.81 | 0.85 | 0.85 | 0.85 | 0.85 |
| Diethanolamine [C4 H11 N O2] | 0.47 | 0.40 | 0.48 | 0.47 | 0.47 | 0.46 | 0.47 | 0.47 |
| GMP | 0.47 | 0.45 | 0.44 | 0.53 | 0.61 | 0.60 | 0.60 | 0.60 |
| glyceraldehyde 3-phosphate/Dihidroxyacetophosphate | 0.46 | 0.68 | 0.66 | 0.69 | 0.71 | 0.71 | 0.71 | 0.71 |
| Homovanillic acid | 0.45 | 0.41 | 0.48 | 0.47 | 0.52 | 0.52 | 0.52 | 0.52 |
| Inosine | 0.45 | 0.52 | 0.56 | 0.62 | 0.63 | 0.64 | 0.65 | 0.65 |
| Lanthionine | 0.45 | 0.59 | 0.64 | 0.66 | 0.69 | 0.70 | 0.70 | 0.70 |
| Pyroglutamic acid | 0.45 | 0.40 | 0.39 | 0.48 | 0.53 | 0.52 | 0.52 | 0.53 |
| H4P3O10 | 0.45 | 0.38 | 0.43 | 0.58 | 0.60 | 0.60 | 0.60 | 0.60 |
| Ser | 0.44 | 0.87 | 0.87 | 0.86 | 0.86 | 0.85 | 0.85 | 0.85 |
| Pyrimidine | 0.44 | 0.38 | 0.38 | 0.37 | 0.43 | 0.48 | 0.48 | 0.48 |
| gamma-Glu-Cys | 0.43 | 1.02 | 0.96 | 0.97 | 0.96 | 0.95 | 0.95 | 0.95 |
| Lactate | 0.43 | 0.41 | 0.42 | 0.42 | 0.55 | 0.56 | 0.55 | 0.55 |
| tetranor-12(R)-HETE [C16 H26 O3] | 0.42 | 0.36 | 0.56 | 0.64 | 0.64 | 0.65 | 0.65 | 0.65 |
| 2-Hydroxyisovaleric acid [C5 H10 O3] | 0.42 | 0.77 | 0.78 | 0.76 | 0.81 | 0.82 | 0.82 | 0.82 |
| Dopamine | 0.42 | 0.37 | 0.43 | 0.52 | 0.55 | 0.56 | 0.56 | 0.57 |
| Adenosine | 0.41 | 0.38 | 0.61 | 0.63 | 0.64 | 0.66 | 0.65 | 0.65 |
| Hexanoylcarnitine [C13 H25 N O4] | 0.41 | 0.37 | 0.42 | 0.46 | 0.46 | 0.46 | 0.46 | 0.46 |
| Lys | 0.41 | 0.35 | 0.34 | 0.53 | 0.54 | 0.55 | 0.55 | 0.55 |
| Uric_acid | 0.41 | 0.40 | 0.43 | 0.43 | 0.42 | 0.47 | 0.47 | 0.48 |
| cyclic FMN [C17 H19 N4 O8 P] | 0.40 | 0.75 | 0.77 | 0.77 | 0.77 | 0.78 | 0.77 | 0.77 |
| Tyr | 0.40 | 0.35 | 0.56 | 0.68 | 0.69 | 0.68 | 0.68 | 0.68 |
| Allantoin | 0.39 | 0.94 | 0.92 | 0.90 | 0.90 | 0.89 | 0.89 | 0.89 |
| 2-C-Methyl-D-erythritol 4-phosphate [C5H13O7P] | 0.39 | 0.34 | 0.63 | 0.65 | 0.66 | 0.68 | 0.68 | 0.68 |
| Taurine | 0.38 | 0.53 | 0.53 | 0.63 | 0.64 | 0.63 | 0.64 | 0.64 |
| Leucinic acid [C6 H12 O3] | 0.37 | 0.48 | 0.86 | 0.89 | 0.90 | 0.90 | 0.90 | 0.90 |
| Carnosine | 0.36 | 0.49 | 0.53 | 0.59 | 0.64 | 0.64 | 0.64 | 0.64 |
| N-Acetylneuraminic_acid | 0.36 | 0.88 | 0.97 | 0.95 | 1.04 | 1.04 | 1.04 | 1.04 |
| CTP | 0.36 | 0.31 | 0.38 | 0.54 | 0.57 | 0.57 | 0.57 | 0.57 |
| 4-Methyl-2-oxopentanoate | 0.36 | 0.49 | 0.95 | 1.01 | 1.00 | 0.99 | 0.99 | 0.99 |
| Pyrophosphate | 0.36 | 0.31 | 0.39 | 0.54 | 0.53 | 0.53 | 0.53 | 0.53 |
| Citrulline | 0.36 | 0.41 | 0.83 | 0.82 | 0.85 | 0.85 | 0.85 | 0.85 |
| Homoserine | 0.35 | 0.85 | 0.93 | 0.92 | 0.91 | 0.91 | 0.91 | 0.91 |
| Homocysteic acid | 0.35 | 0.74 | 0.70 | 0.69 | 0.68 | 0.70 | 0.71 | 0.71 |
| Thr | 0.35 | 0.85 | 0.93 | 0.92 | 0.91 | 0.91 | 0.91 | 0.91 |
| Caffeine | 0.35 | 0.38 | 0.41 | 0.48 | 0.51 | 0.51 | 0.51 | 0.51 |
| 4-Hydroxyphenylacetic acid [C8 H8 O3] | 0.35 | 0.69 | 0.71 | 0.70 | 0.69 | 0.70 | 0.70 | 0.70 |
| His | 0.34 | 0.30 | 0.37 | 0.59 | 0.58 | 0.58 | 0.58 | 0.58 |
| Glutathione_oxidized form | 0.34 | 0.37 | 0.58 | 0.63 | 0.63 | 0.64 | 0.64 | 0.64 |
| Thymidine | 0.34 | 0.44 | 0.78 | 0.77 | 0.80 | 0.80 | 0.80 | 0.80 |
| Furoic acid | 0.34 | 0.83 | 0.81 | 0.79 | 0.79 | 0.81 | 0.81 | 0.81 |
| Citrate | 0.34 | 0.84 | 0.81 | 0.79 | 0.79 | 0.82 | 0.81 | 0.81 |
| Fructose 6-Phosphate | 0.34 | 0.41 | 0.47 | 0.53 | 0.62 | 0.62 | 0.62 | 0.62 |
| Pyridoxal-5'-phosphate | 0.33 | 0.66 | 0.72 | 0.73 | 0.72 | 0.73 | 0.73 | 0.73 |
| Serotonin | 0.33 | 0.29 | 0.34 | 0.52 | 0.56 | 0.55 | 0.55 | 0.55 |
| alpha-HMB (Hydroxy-methylbutyrate) | 0.33 | 0.43 | 0.76 | 0.76 | 0.84 | 0.84 | 0.84 | 0.84 |
| Glycerol | 0.32 | 0.94 | 0.89 | 0.90 | 0.91 | 0.91 | 0.91 | 0.91 |
| Creatine | 0.32 | 0.31 | 0.43 | 0.62 | 0.68 | 0.68 | 0.68 | 0.68 |
| 3,4-Dihydroxymandelic acid [C8 H8 O5] | 0.31 | 0.35 | 0.48 | 0.53 | 0.57 | 0.57 | 0.57 | 0.57 |
| Octylphosphonic acid [C8 H19 O3 P] | 0.31 | 0.75 | 0.73 | 0.71 | 0.70 | 0.70 | 0.70 | 0.70 |
| 4-Methylbenzoic acid [C8 H8 O2] | 0.30 | 0.59 | 0.61 | 0.61 | 0.61 | 0.62 | 0.62 | 0.62 |
| Ferulic acid [C10 H10 O4] | 0.30 | 0.96 | 0.90 | 0.89 | 0.91 | 0.93 | 0.92 | 0.92 |
| Sinapinic acid [C11 H12 O5] | 0.29 | 0.68 | 0.77 | 0.75 | 0.74 | 0.74 | 0.74 | 0.74 |
| Pyruvate | 0.29 | 0.59 | 0.59 | 0.58 | 0.63 | 0.64 | 0.64 | 0.64 |
| 2-Aminobutyric acid | 0.29 | 0.51 | 0.58 | 0.59 | 0.61 | 0.64 | 0.64 | 0.64 |
| Phenylpyruvic acid [C9 H8 O3] | 0.29 | 0.68 | 0.64 | 0.63 | 0.66 | 0.69 | 0.69 | 0.69 |
| gamma-Glu-Asp | 0.29 | 0.66 | 0.71 | 0.70 | 0.69 | 0.69 | 0.69 | 0.69 |
| N3,N4-Dimethyl-L-arginine [C8 H18 N4 O2] | 0.28 | 0.80 | 0.77 | 0.76 | 0.78 | 0.79 | 0.79 | 0.79 |
| 2-Keto-4-methylthiobutyric_acid | 0.28 | 0.92 | 0.93 | 0.92 | 0.91 | 0.91 | 0.91 | 0.91 |
| Succinic anhydride [C4 H4 O3] | 0.27 | 0.71 | 0.67 | 0.66 | 0.67 | 0.67 | 0.67 | 0.67 |
| Tartaric acid [C4 H6 O6] | 0.27 | 0.45 | 0.53 | 0.53 | 0.58 | 0.60 | 0.60 | 0.60 |
| Thiosulfate | 0.27 | 0.42 | 0.41 | 0.41 | 0.53 | 0.59 | 0.60 | 0.60 |
| 3-Methyladipic acid [C7 H12 O4] | 0.26 | 0.80 | 0.97 | 0.95 | 0.97 | 0.96 | 0.96 | 0.96 |
| alpha-Keto-beta-methylvaleric acid | 0.26 | 0.80 | 0.75 | 0.75 | 0.74 | 0.74 | 0.74 | 0.74 |
| O-Acetyl-L-serine | 0.26 | 0.22 | 0.62 | 0.60 | 0.63 | 0.67 | 0.67 | 0.67 |
| GTP | 0.26 | 0.24 | 0.36 | 0.54 | 0.56 | 0.56 | 0.56 | 0.56 |
| Acetylphosphate | 0.25 | 0.49 | 0.56 | 0.58 | 0.65 | 0.65 | 0.65 | 0.65 |
| Cystathionine | 0.25 | 0.38 | 0.42 | 0.54 | 0.58 | 0.59 | 0.59 | 0.59 |
| ADP | 0.24 | 0.82 | 0.77 | 0.87 | 0.85 | 0.85 | 0.85 | 0.85 |
| Indole-3-butyric acid | 0.24 | 0.28 | 0.77 | 0.76 | 0.75 | 0.76 | 0.75 | 0.75 |
| Alanine | 0.23 | 0.27 | 0.38 | 0.50 | 0.54 | 0.55 | 0.55 | 0.55 |
| Hydroxyproline | 0.23 | 0.21 | 0.29 | 0.33 | 0.34 | 0.39 | 0.39 | 0.39 |
| Asp-Glu-Ser [probable] | 0.23 | 0.88 | 0.93 | 0.91 | 0.90 | 0.90 | 0.89 | 0.89 |
| Creatinine | 0.22 | 0.84 | 0.79 | 0.81 | 0.80 | 0.80 | 0.80 | 0.80 |
| Fatty acid (C7:0) [C7 H14 O2] | 0.22 | 0.98 | 0.93 | 0.91 | 0.90 | 0.89 | 0.89 | 0.89 |
| Asp-Asp-Glu [probable] | 0.22 | 0.69 | 0.65 | 0.65 | 0.68 | 0.67 | 0.68 | 0.68 |
| 2-Hydroxyphenylacetic acid [C8 H8 O3] | 0.22 | 0.33 | 0.37 | 0.38 | 0.39 | 0.39 | 0.39 | 0.40 |
| Hexose | 0.22 | 0.99 | 0.93 | 0.92 | 0.93 | 0.92 | 0.92 | 0.92 |
| 4-Hydroxyphenylpyruvic acid | 0.22 | 0.30 | 0.28 | 0.34 | 0.34 | 0.38 | 0.39 | 0.39 |
| beta-Keto-iso-valeric acid | 0.20 | 0.38 | 0.48 | 0.50 | 0.53 | 0.52 | 0.52 | 0.53 |
| Terephthalic acid [C8 H6 O4] | 0.20 | 0.21 | 0.41 | 0.44 | 0.45 | 0.45 | 0.46 | 0.46 |
| ATP | 0.20 | 0.19 | 0.42 | 0.56 | 0.58 | 0.58 | 0.58 | 0.58 |
| 5-Hydroxyanthranilic acid | 0.19 | 0.78 | 0.84 | 0.85 | 0.84 | 0.84 | 0.84 | 0.84 |
| Gly-Pro | 0.19 | 0.45 | 0.59 | 0.60 | 0.59 | 0.59 | 0.59 | 0.60 |
| UTP | 0.18 | 0.17 | 0.34 | 0.52 | 0.54 | 0.54 | 0.54 | 0.54 |
| L-Ergothioneine [C9 H15 N3 O2 S] | 0.17 | 0.26 | 0.36 | 0.39 | 0.39 | 0.43 | 0.44 | 0.44 |
| Mevalonate-P | 0.17 | 0.41 | 0.39 | 0.38 | 0.44 | 0.56 | 0.56 | 0.56 |
| 3Phospho-D-glycerate + 2-Phospho-D-glycerate | 0.17 | 0.66 | 0.62 | 0.60 | 0.61 | 0.60 | 0.61 | 0.61 |
| Stearamide [C18 H37 N O] | 0.15 | 0.17 | 0.22 | 0.43 | 0.43 | 0.43 | 0.43 | 0.43 |
| UMP | 0.14 | 0.16 | 0.44 | 0.58 | 0.58 | 0.59 | 0.59 | 0.59 |
| gamma-Glu-Leu | 0.14 | 0.36 | 0.42 | 0.42 | 0.47 | 0.52 | 0.52 | 0.52 |
| Pimelic acid [C7 H12 O4] | 0.13 | 0.66 | 0.62 | 0.60 | 0.66 | 0.67 | 0.68 | 0.68 |
| 1-Methyluracil [C5H6N2O2] | 0.13 | 0.79 | 0.80 | 0.83 | 0.82 | 0.83 | 0.82 | 0.82 |
| 2-Isopropylmalic acid [C7 H12 O5] | 0.12 | 0.62 | 0.83 | 0.85 | 0.91 | 0.90 | 0.91 | 0.91 |
| Glycerol_3-phosphate | 0.11 | 0.11 | 0.17 | 0.21 | 0.20 | 0.23 | 0.23 | 0.24 |
| Hexadecanamide [C16 H33 N O] | 0.11 | 0.28 | 0.27 | 0.38 | 0.44 | 0.44 | 0.44 | 0.44 |
| Dihydroxyacetone phosphate  +DL-Glyceraldehyde 3-phosphate | 0.11 | 0.31 | 0.67 | 0.69 | 0.68 | 0.68 | 0.68 | 0.68 |
| Val | 0.10 | 0.12 | 0.36 | 0.57 | 0.57 | 0.57 | 0.57 | 0.57 |
| 4-Hydroxyphenyllactic acid [C9 H10 O4] | 0.10 | 0.60 | 0.76 | 0.75 | 0.74 | 0.74 | 0.74 | 0.74 |
| D-Glucopyranuronate [C6 H10 O7] | 0.09 | 0.15 | 0.29 | 0.33 | 0.37 | 0.37 | 0.37 | 0.37 |
| N6-Me-Adenosine [C11 H15 N5 O4] | 0.09 | 0.68 | 0.63 | 0.64 | 0.65 | 0.68 | 0.68 | 0.68 |
| 5,6-Dihydrouracil/N-Cyano-L-alanine [C4 H6 N2 O2] | 0.08 | 0.45 | 0.57 | 0.56 | 0.55 | 0.55 | 0.55 | 0.55 |
| Acetoacetate | 0.08 | 0.15 | 0.29 | 0.29 | 0.52 | 0.53 | 0.54 | 0.54 |
| Thymine | 0.07 | 0.07 | 0.08 | 0.10 | 0.38 | 0.38 | 0.40 | 0.40 |
| 5-phosphoribosyldiphosphate | 0.07 | 0.12 | 0.34 | 0.53 | 0.53 | 0.54 | 0.54 | 0.54 |
| 12-Oxo phytodienoic acid [C18 H28 O3] | 0.07 | 0.07 | 0.30 | 0.43 | 0.67 | 0.67 | 0.67 | 0.67 |
| Phe | 0.06 | 0.06 | 0.27 | 0.53 | 0.54 | 0.54 | 0.54 | 0.54 |
| Tryp | 0.06 | 0.10 | 0.33 | 0.57 | 0.57 | 0.57 | 0.57 | 0.57 |
| 5-Phospho-α-D-ribose 1-diphosphate [C5 H13 O14 P3] | 0.06 | 0.12 | 0.33 | 0.53 | 0.53 | 0.54 | 0.54 | 0.54 |
| Dodecanedioic acid [C12 H22 O4] | 0.06 | 0.18 | 0.79 | 0.82 | 0.83 | 0.84 | 0.84 | 0.84 |
| 5-Phospho-alpha-D-ribose1-diphosphate [C5 H13 O14 P3] | 0.06 | 0.12 | 0.33 | 0.53 | 0.53 | 0.54 | 0.54 | 0.54 |
| Ribose5-phosphate + Ribulose5-phosphate | 0.06 | 0.45 | 0.48 | 0.57 | 0.58 | 0.58 | 0.58 | 0.58 |
| Indole-3-propionic acid | 0.06 | 0.27 | 0.26 | 0.29 | 0.32 | 0.32 | 0.34 | 0.34 |
| Butyrate | 0.05 | 0.96 | 0.90 | 0.89 | 0.88 | 0.89 | 0.89 | 0.88 |
| 2-Aminonicotinic acid [C6 H6 N2 O2] | 0.05 | 0.25 | 0.26 | 0.29 | 0.32 | 0.36 | 0.39 | 0.39 |
| 2-Methyl-3-Hydroxybutyric acid [C5 H10 O3] | 0.04 | 0.77 | 0.80 | 0.78 | 0.79 | 0.81 | 0.81 | 0.81 |
| beta-hydroxy-iso-butyrate | 0.04 | 0.08 | 0.47 | 0.46 | 0.60 | 0.62 | 0.62 | 0.62 |
| Citraconic acid | 0.03 | 0.82 | 0.79 | 0.78 | 0.78 | 0.80 | 0.80 | 0.80 |
| FAD | 0.03 | 0.04 | 0.87 | 0.85 | 0.84 | 0.85 | 0.85 | 0.84 |
| Norepinephrine | 0.02 | 0.56 | 0.68 | 0.67 | 0.66 | 0.66 | 0.67 | 0.67 |
| Cytosine.1 | 0.02 | 0.59 | 0.60 | 0.60 | 0.59 | 0.59 | 0.59 | 0.59 |
| Uridine | 0.02 | 0.36 | 0.94 | 0.93 | 0.92 | 0.93 | 0.93 | 0.93 |
| alpha-D-Xylose1-phosphate [C5 H11 O8 P] | 0.01 | 0.36 | 0.46 | 0.48 | 0.71 | 0.71 | 0.71 | 0.71 |
| Fatty acid (C6:0) [C6 H12 O2] | 0.01 | 0.12 | 0.59 | 0.65 | 0.67 | 0.67 | 0.67 | 0.67 |
| Citicoline | 0.01 | 0.11 | 0.36 | 0.56 | 0.56 | 0.56 | 0.56 | 0.56 |
| 2,3-Dihydroxybenzoate [C7 H6 O4] | 0.00 | 0.33 | 0.33 | 0.36 | 0.46 | 0.46 | 0.46 | 0.46 |

|  | Fold change | p-value |
| --- | --- | --- |
| GT [C6 H12 N2 O4] | 0.558 | 0.000 |
| 5-Dehydroquinic acid [C7 H10 O6] | 0.899 | 0.000 |
| Oxoproline | 0.581 | 0.002 |
| 4-Aminobutyric acid (GABA) | 6.149 | 0.002 |
| Erythose [C4 H8 O4] | 0.775 | 0.002 |
| Allantoin | 0.686 | 0.002 |
| Gln | 0.586 | 0.003 |
| beta-Hydroxybutyrate | 0.617 | 0.003 |
| 5-Hydroxyindole-3-acetic acid | 2.002 | 0.003 |
| Mandelic acid [C8 H8 O3] | 0.863 | 0.005 |
| Cys | 0.634 | 0.006 |
| gamma-Glu-Ser | 0.703 | 0.007 |
| Indole-3-acetyl-L-aspartic acid [C14 H14 N2 O5] | 0.919 | 0.007 |
| Asymmetric dimethylarginine | 1.044 | 0.009 |
| 3-hydroxy-Anthranilic acid | 0.327 | 0.010 |
| Cystine | 0.571 | 0.012 |
| Dimethylglycine | 0.788 | 0.016 |
| Methylnicotinamide | 0.668 | 0.020 |
| cyclic FMN [C17 H19 N4 O8 P] | 0.686 | 0.020 |
| 3-Indoleacetic acid | 1.051 | 0.021 |
| Picolinic acid | 0.774 | 0.022 |
| N3,N4-Dimethyl-L-arginine [C8 H18 N4 O2] | 0.716 | 0.023 |
| Cortisol | 0.495 | 0.023 |
| alpha-keto-butyrate | 0.695 | 0.024 |
| Nicotinic_Acid | 0.772 | 0.024 |
| Choline | 0.714 | 0.027 |
| Isopentenyl pyrophosphate | 1.267 | 0.027 |
| 2-oxoglutarate | 0.715 | 0.029 |
| Pyroglutamic acid | 0.737 | 0.029 |
| gamma-Glu-Trp | 1.154 | 0.031 |
| Succinate | 0.580 | 0.032 |
| ADP-ribose 2'-phosphate [C15 H24 N5 O17 P3] | 0.141 | 0.032 |
| Orthophosphate | 0.820 | 0.035 |
| Dehydroepiandrosterone (DHEA) [C19 H28 O2] | 0.483 | 0.038 |
| alpha-Keto-iso-valeric acid | 0.835 | 0.038 |
| Urea | 0.639 | 0.039 |
| Dmethyl-Lys | 1.708 | 0.040 |
| HPO4 | 0.837 | 0.041 |
| Citraconic acid | 0.747 | 0.045 |
| gamma-Glu-Glu | 0.902 | 0.046 |
| Cytidine monophosphate | 8.233 | 0.046 |
| Dihydroorotate | 1.256 | 0.046 |
| Quinolinate | 0.781 | 0.048 |
| 2,6-Pyridinedicarboxylic acid [C7 H5 N O4] | 0.781 | 0.048 |

**Supplementary table S8. Metabolites showed significant change by the first ECT in the remission group**

Abbreviations: Cys, cysteine; Gln, glutamine.

Amino acids and oligo-peptides are highlighted in red.

**Supplementary Figure S1. PLS-DA cross-validation details for Fig. 1A**


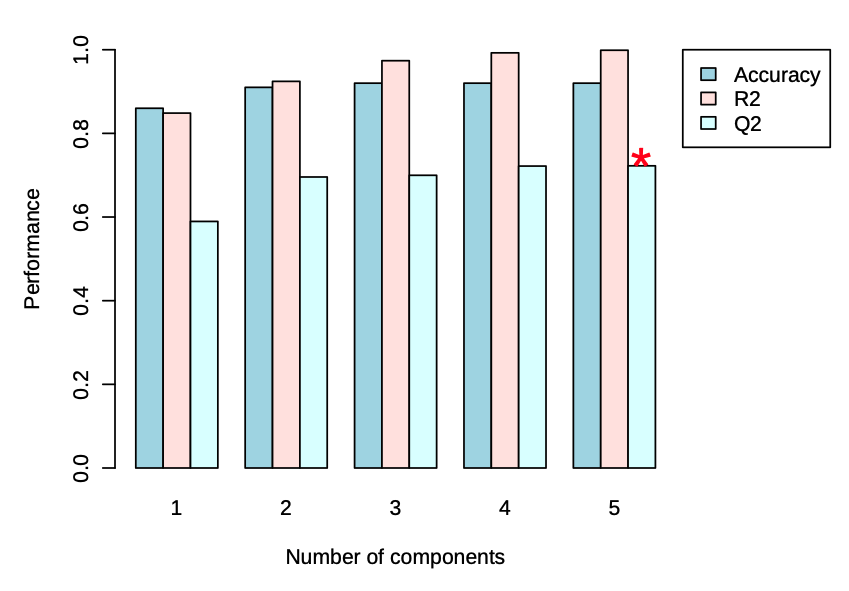


Plots obtained by cross-validation method (leave-one-out cross-validation [LOOCV]) applied on partial least squares-discriminant analysis (PLS-DA) data. The PLS-DA cross-validation data showed cumulative values of R2 = 0.999 and Q2 = 0.722, indicating good clustering, and demonstrated a good distinction between the two groups.

**Supplementary Figure S2. HCA to classify the metabolites with increased or decreased plasma levels in patients with MDD compared with in HCs at baseline**


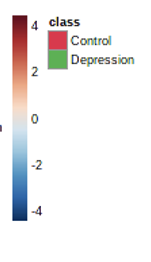


**Cluster-1**

Carnitine derivatives

Purine metabolites

Trp and its IDO catabolites

γGlutamyl-dipeptides

Urea

GABA

**Cluster-2**

Cortisol

Choline

N-acetyl aspartate

Ketone body

Inorganic phosphates

Healthy control

Depression

HCA showing metabolites those levels were higher in patients with MDD in Cluster-1, while those levels were lower in Cluster-2, compared with HCs.

Abbreviations: GABA, gamma-aminobutyric acid; HC, healthy control; IDO, indoleamine 2,3-dioxygenase; MDD, major depressive disorder; Trp, tryptophan.

**Supplementary Figure S3. Change in MADRS or QIDS scores during ECT for each patient**

The severity of depression gradually improved over the course of ECT.

Abbreviations: ECT, electroconvulsive therapy; MADRS, Montgomery–Asberg Depression Rating Scale; QIDS, Quick Inventory of Depressive Symptomatology.

**Supplementary Figure S4. Scatter plots of metabolite showing significant correlations with MADRS and/or QIDS**


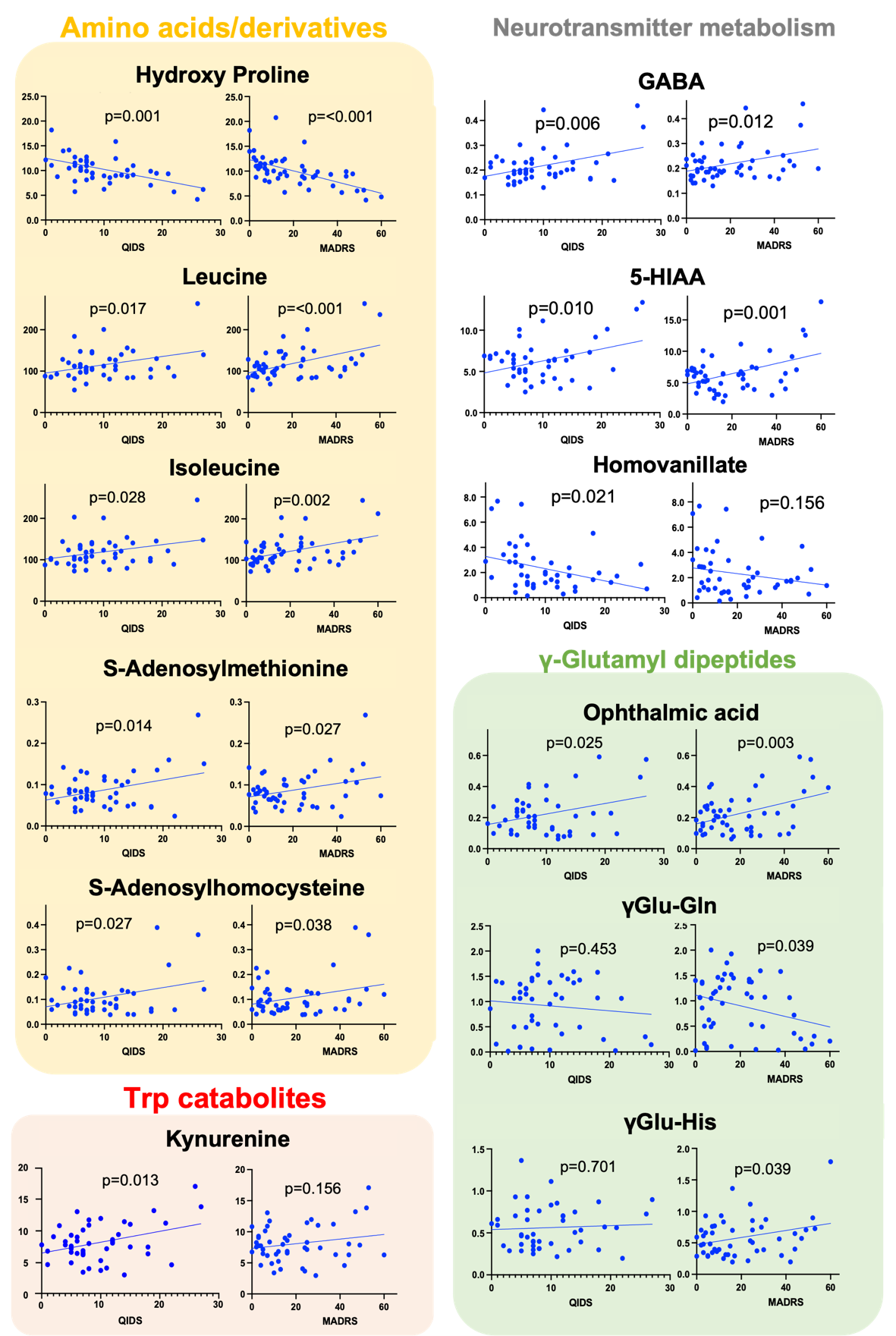


Several amino acids and those derivatives, Trp catabolites, neurotransmitter metabolism, and γ-glutamyl dipeptides showed significant correlations with depression severity during ECT.

Abbreviations: ECT, electroconvulsive therapy; GABA, gamma-aminobutyric acid; MADRS, Montgomery–Asberg Depression Rating Scale; QIDS, Quick Inventory of Depressive Symptomatology; Trp, tryptophan; γGlu-Glu, gamma glutamyl-glutamic acid; γGlu-His, glutamyl histidine; 5-HIAA, 5-hydroxyindoleacetic acid.

**Supplementary Figure S5. The metabolites showed significant changes in their levels only in the remission group**


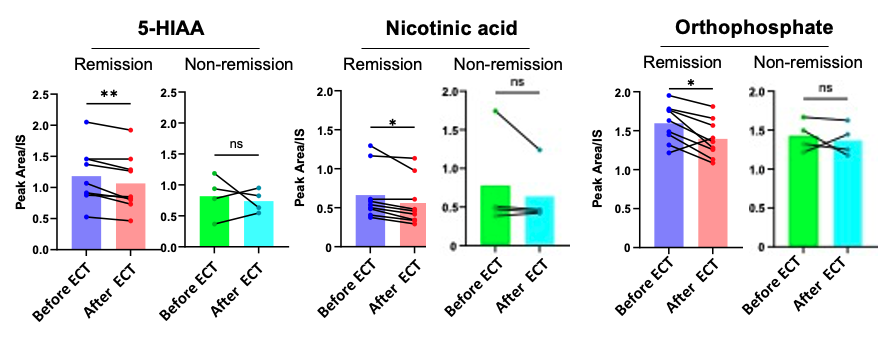


The changes in levels of some metabolites levels significantly changed by the first ECT in the remission group, whereas not in the non-remission group.

Abbreviations: ECT, electroconvulsive therapy; 5-HIAA, 5-hydroxyindoleacetic acid.

**Supplementary Figure S6. Flow of blood sampling and metabolome measurement**


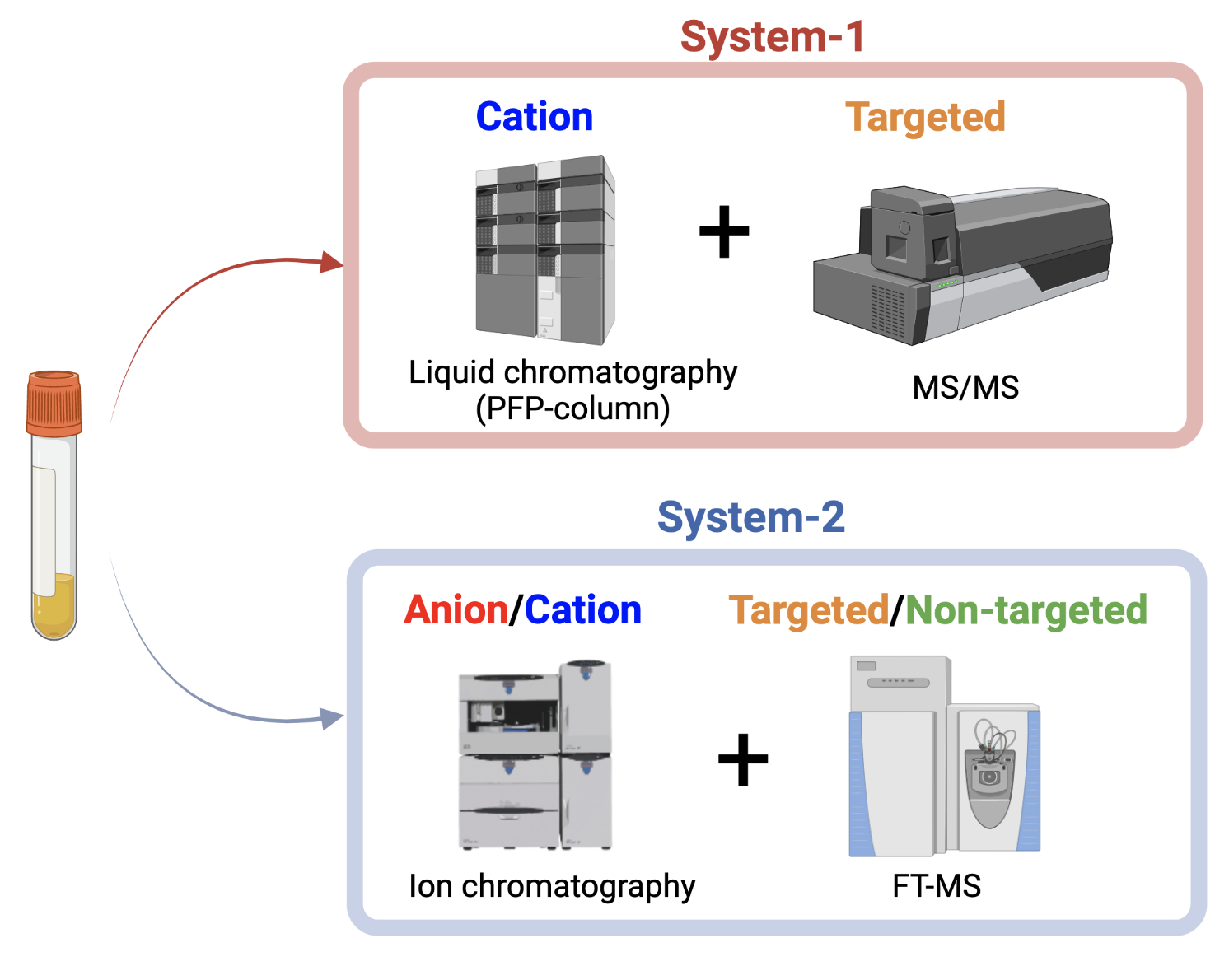


Amino acids

Nucleosides

Oligopeptides

Targeted + Non-targeted

Targeted

Amino acids

Nucleotides

Oligopeptides

Phosphorylated

metabolites

Organic acids

(A)

(C)

(B)

Targeted (inhouse library)

+ Identification validated metabolites

Non-targeted

(Database assigned only)


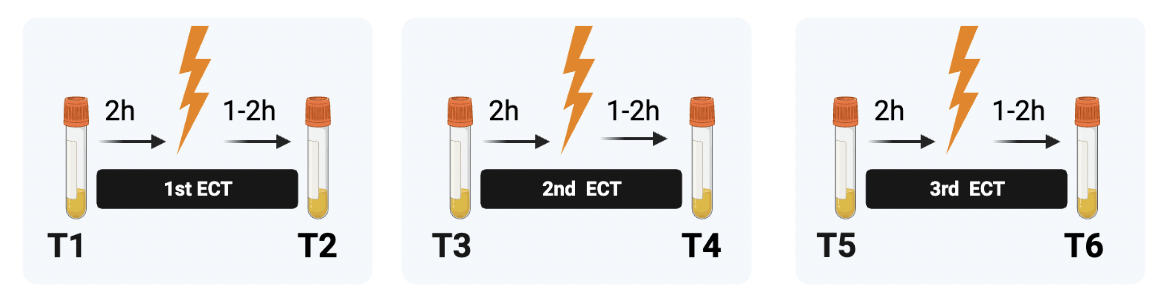

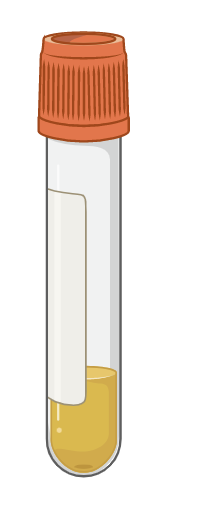


3-7days

**1st ECT**

**3rd ECT**

**last ECT**

**T7**

Venous blood samples were collected before and after the first (T1 and T2, respectively), third (T3 and T4, respectively), and final ECT sessions (T5 and T6, respectively), and 3–7 days after the final ECT session (T7) (A).

Each plasma sample was divided into two portions. One portion was subjected to targeted metabolome measurement by LC-MS/MS specialized for cation measurement (System-1). The other one was used for targeted and non-targeted metabolome analysis by IC-FT-MS, which can measure both anions and cations (System-2) (B).

Targeted and non-targeted metabolomic analysis identified 217 and 140 components in plasma, respectively (C).

Abbreviations: ECT, electroconvulsive therapy; IC-FT-MS, ion chromatography with Fourier transform mass spectrometry; LC-MS/MS, Liquid chromatography with tandem mass spectrometry.
